# Supplementary material for: Transcriptome-guided development of a fibrosis-reversal compound reduces skin scarring and allows regeneration via mitochondrial uncoupling
Source: Cell Rep Med. 2026 May 19;7(5):102821. doi: 10.1016/j.xcrm.2026.102821 (PMC13198262; doi:10.1016/j.xcrm.2026.102821)
Supplement: Document S1. Figures S1–S11, Tables S1–S7, and supplemental references [file mmc1.pdf]

Cell Reports Medicine, Volume 7

Supplemental information

Transcriptome-guided development of a fibrosis-reversal compound reduces skin scarring and allows regeneration via mitochondrial uncoupling

Chun-Ye Chen, Ruilin Xu, Mingguang Mo, Jiahao Wu, Jun Chi, Zhang-Rui Wu, Yi Wang, Xin-Cao Zhong, Xiao-Ying Lin, Yang Liu, Jingdong Wu, Huaan Fang, Hongli Jia, Hongsen Bi, Yong Yang, Wei-Qiang Tan, and Yang Zhao

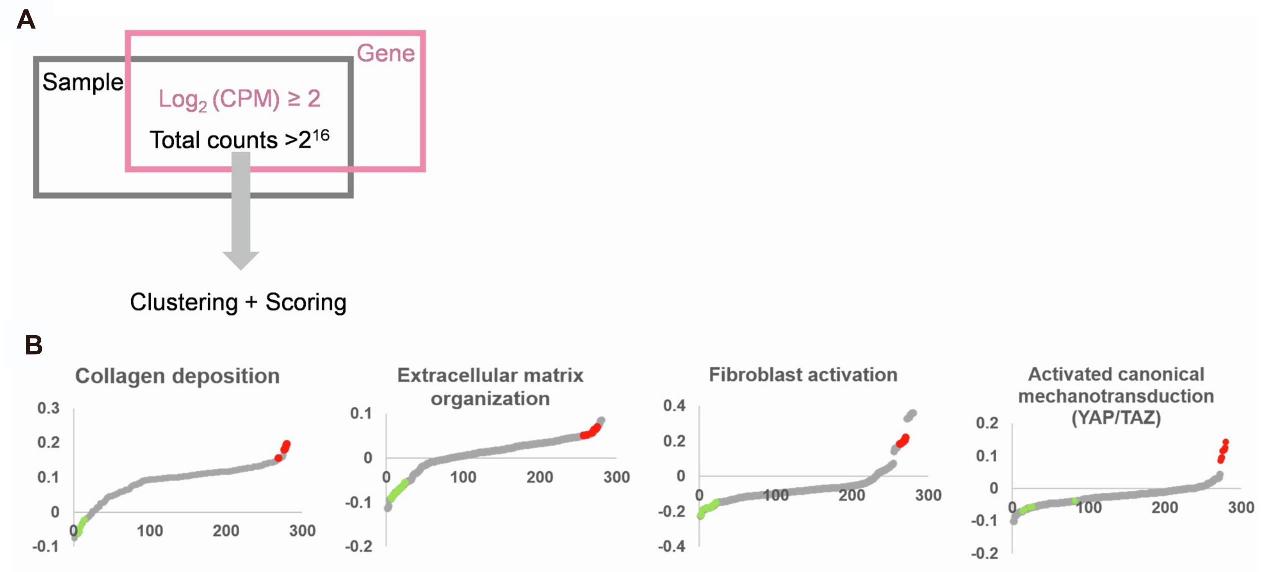

Compound	Rank
Sorafenib	1
L-Ascorbic acid	2
ICG-001	3
Rottlerin	4
bFGF	5

Compound	Rank
Sorafenib	1
L-Ascorbic acid	2
Rottlerin	3
ICG-001	4
SB431542	5

Compound	Rank
Sorafenib	1
Rottlerin	2
L-Ascorbic acid	3
SB431542	4
ICG-001	5

Compound	Rank
Sorafenib	1
SB431542	2
Nintedanib	3
Rottlerin	4
SAR-100842	5

Figure S1. DRUG-seq2 transcriptomic data preprocessing and gene set scoring, related to Figure 1. (A) Specific plan for DRUG-seq2 data preprocessing. (B) Module scores for four fibrosis-associated gene sets were calculated using "addModuleScore" function in Seurat (R package). Dots denote treatment groups: red, TGF-β1; green, Rottlerin. Top five candidates per set identified based on scores.

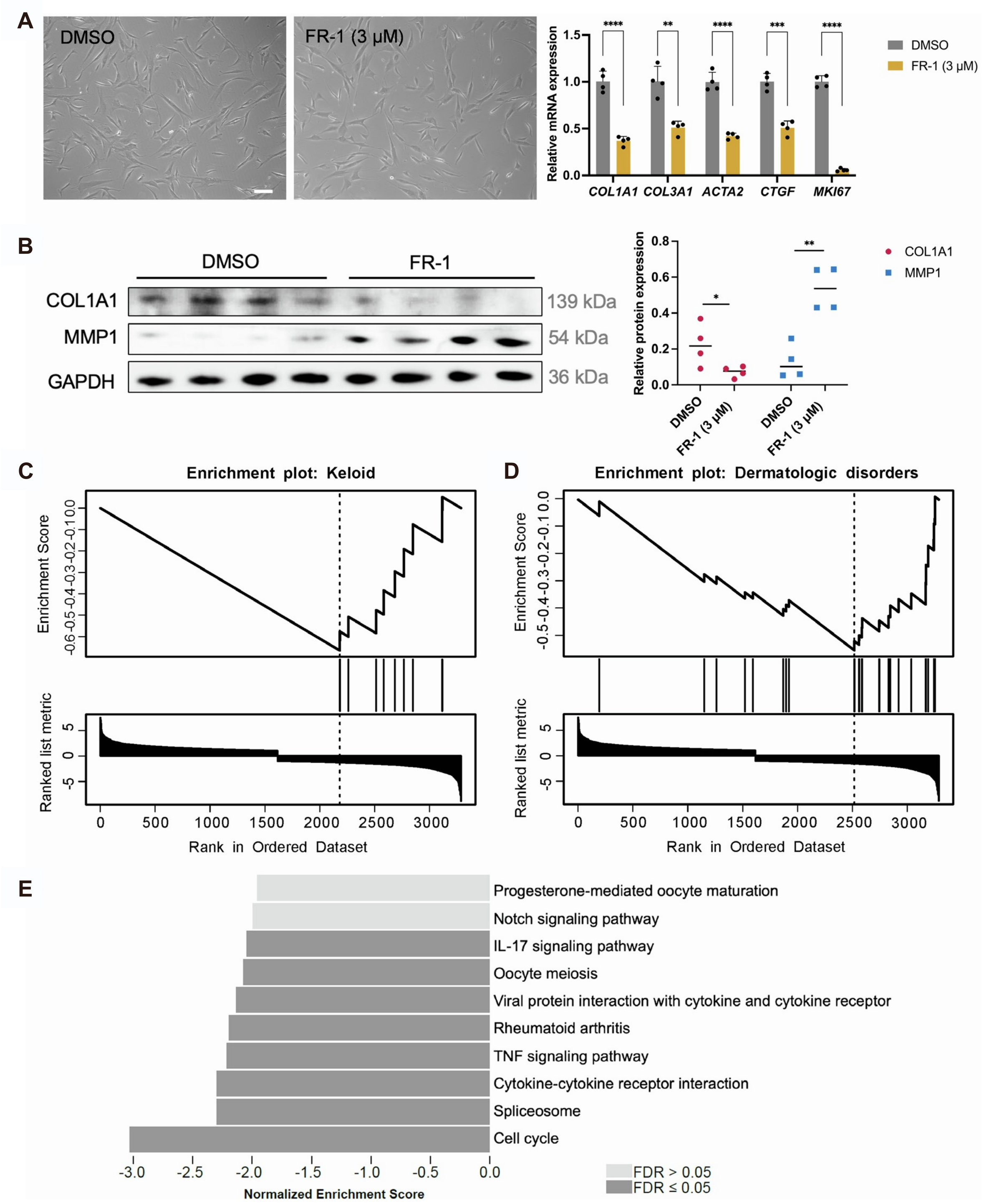

Figure S2. FR-1 reverses fibrotic phenotypes in African-descent keloid fibroblasts and drives superior transcriptomic regulation relative to Rottlerin, related to Figure 2. (A) Representative bright-field images (scale bar: 100 μm) and RT-qPCR analysis of fibrosis-related genes (*COL1A1, COL3A1, ACTA2, CTGF*) and the proliferation marker *MKI67* in ATCC CRL-1762 cells (Black donor) treated with DMSO or 3 μM FR-1. (B) Western blot analysis of COL1A1 and MMP1 protein levels in cells treated as in (A). (C, D) GSEA plots depicting significant downregulation of DisGeNET gene sets (keloid, dermatologic disorders) in FR-1 compared to Rottlerin. Vertical lines represent gene positions in the ranked list; the black curve indicates the enrichment score (ES) trajectory. (E) Top 10 significantly downregulated KEGG pathways in FR-1 versus Rottlerin, ranked by normalized enrichment score. Gray bars denote downregulated pathways; color intensity corresponds to FDR significance. Data are mean ± SD (n=4), n represents the number of independent biological replicates. $*P < 0.05$; $**P < 0.01$; $***P < 0.001$; $****P < 0.0001$ vs. DMSO by Student's t test.

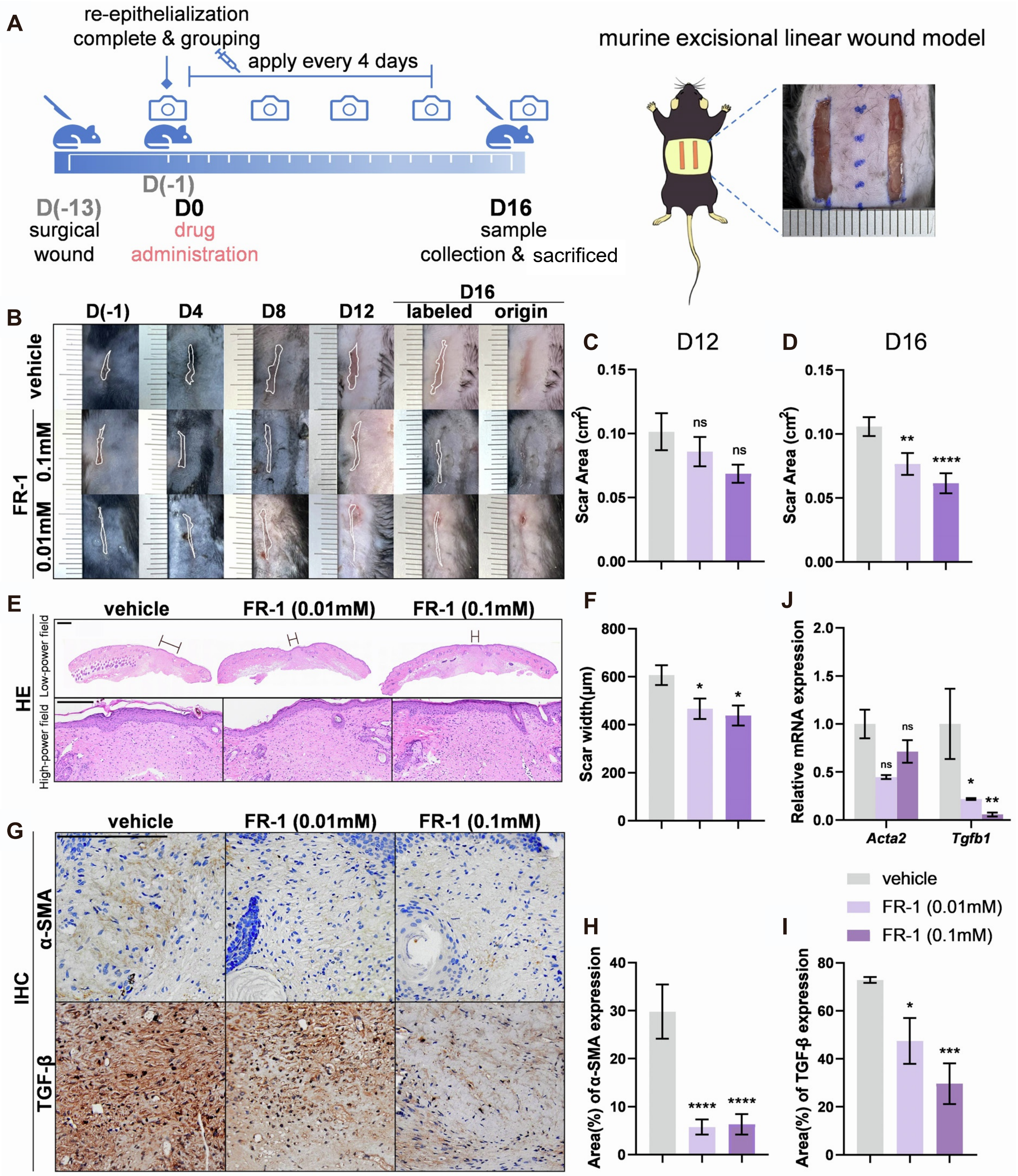

Figure S3. FR-1 injection reduces scar formation in a murine linear excisional wound model, related to Figure 3. (A) Schematic and timeline of the murine linear excisional wound model (1.5 cm × 0.2 cm wounds, 0.4 cm lateral to midline). (B) Representative images of scars in mice treated with vehicle, 0.1 mM FR-1, or 0.01 mM FR-1 at indicated time points (white boxes: scar areas), and quantification of scar area on (C) D12 and (D) D16 (n = 10 scars from 5 mice per group). (E) H&E-stained scar tissues on D16 (black line: scar width; scale bar: 500 μm, high-magnification: 200 μm), and (F) scar width quantification (n=5 mice). (G) Immunohistochemistry for α-SMA and TGF-β1 in D16 scar tissues (scale bar: 200 μm), and quantification of (H) α-SMA and (I) TGF-β1 expression (n = 5 HPFs from 2 mice). (J) RT-qPCR analysis of fibrotic marker mRNA expression (*Acta2, Tgfb1*) in D16 scars (n = 3 mice). Data are mean ± SEM, n represents the number of independent biological replicates. $*P < 0.05$; $**P < 0.01$; $***P < 0.001$; $****P < 0.0001$; ns, not statistically significant vs. vehicle by one-way ANOVA.

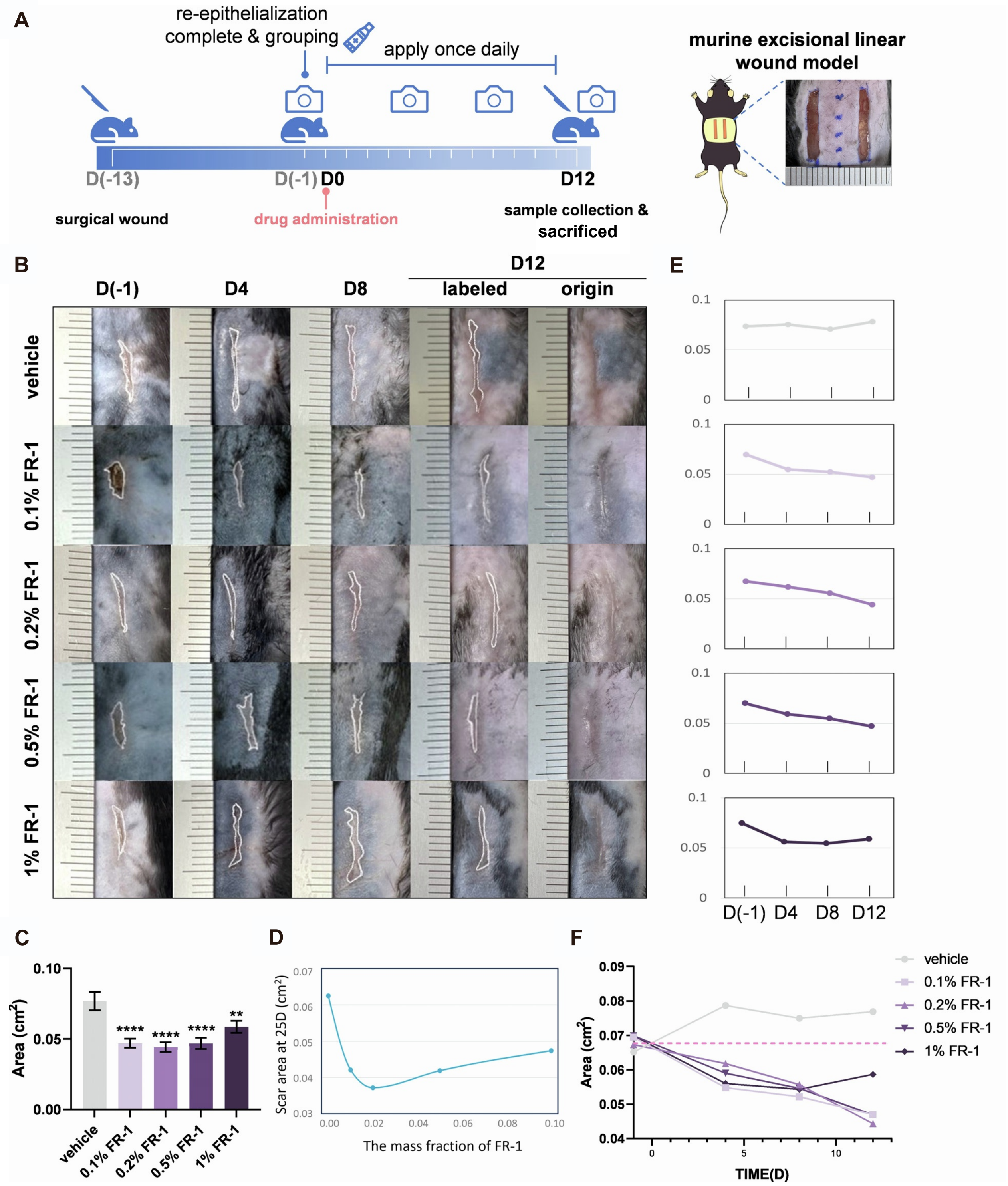

Figure S4. Dose-dependent therapeutic efficacy of FR-1 ointment on scar formation in a murine linear excisional wound model, related to Figure 3. (A) Schematic and timeline of the murine linear excisional wound model (1.5 cm × 0.2 cm wounds, 0.4 cm lateral to midline). (B) Representative images of scar formation in mice treated with vehicle, 0.1%, 0.2%, 0.5% and 1% FR-1 ointment at indicated time points (white boxes: scar areas; n=10 scars from 5 mice per group). (C) Bar graph and (D) line graph showing quantitative data of scar area on D12. (E) Individual-group temporal profiles of scar area. (F) Combined line graph showing temporal changes in scar area for all groups. Data are mean ± SEM, n represents the number of independent biological replicates. $**P < 0.01$; $****P < 0.0001$ vs. vehicle by one-way ANOVA.

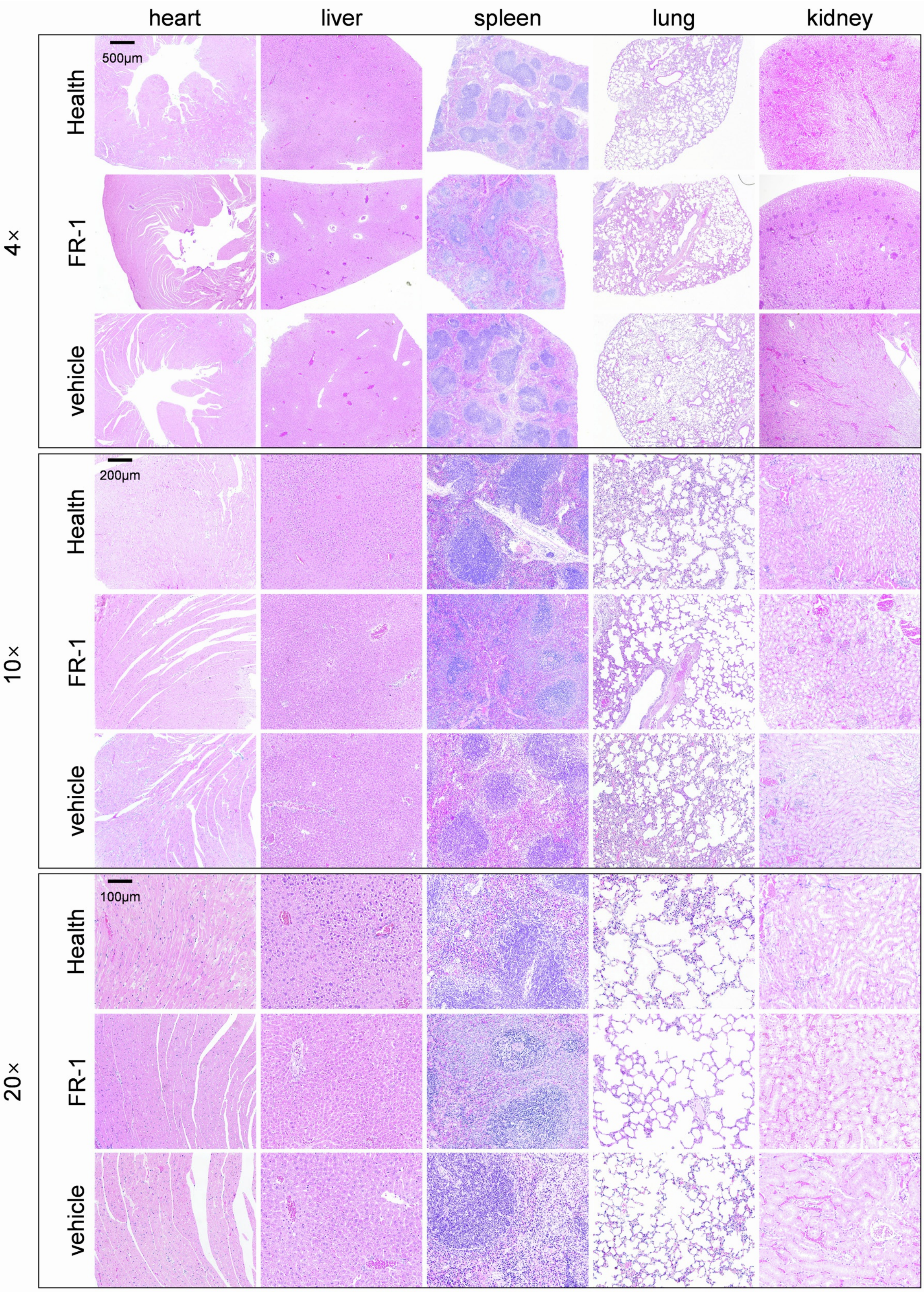

Figure S5. Histopathological analysis of major organs from the murine linear excisional wound model, related to Figure 3. Representative H&E staining images of the heart, liver, spleen, lung, and kidney from healthy mice, topical FR-1-treated mice, and vehicle-treated mice at the experimental endpoint.

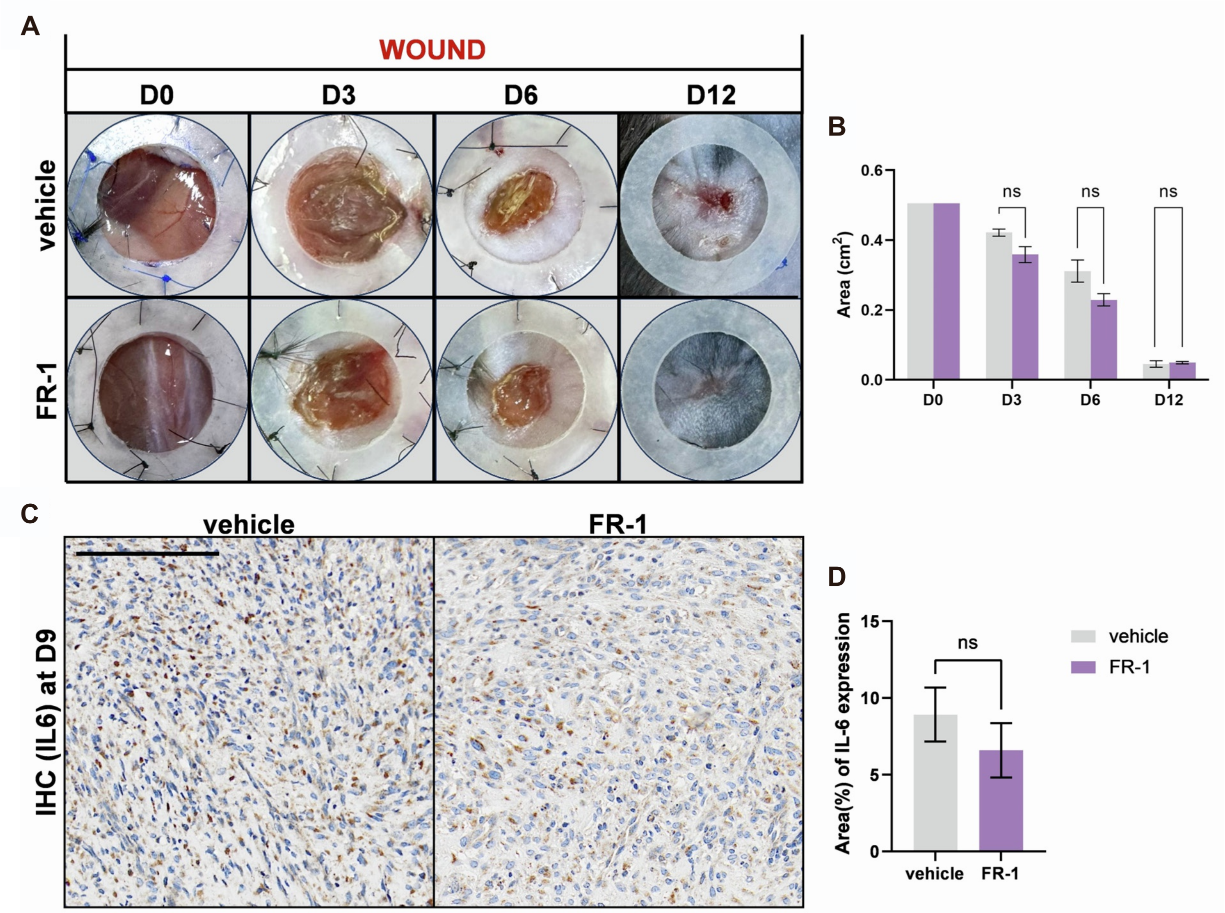

Figure S6. FR-1 did not delay acute wound closure, related to Figure 4. (A) Representative images of wound healing progression (D0-D12) with vehicle or FR-1 treatment. (B) Quantification of wound areas over time (n = 8–10 scars from 4–5 mice per group). (C) Immunohistochemistry for IL-6 in D9 wound tissues (scale bar: 200 μm), and quantification of (D) IL-6 expression (n = 4 HPFs from 2 mice). Data are represented as mean ± SEM, n represents the number of independent biological replicates, ns, not statistically significant by Student’s t test.

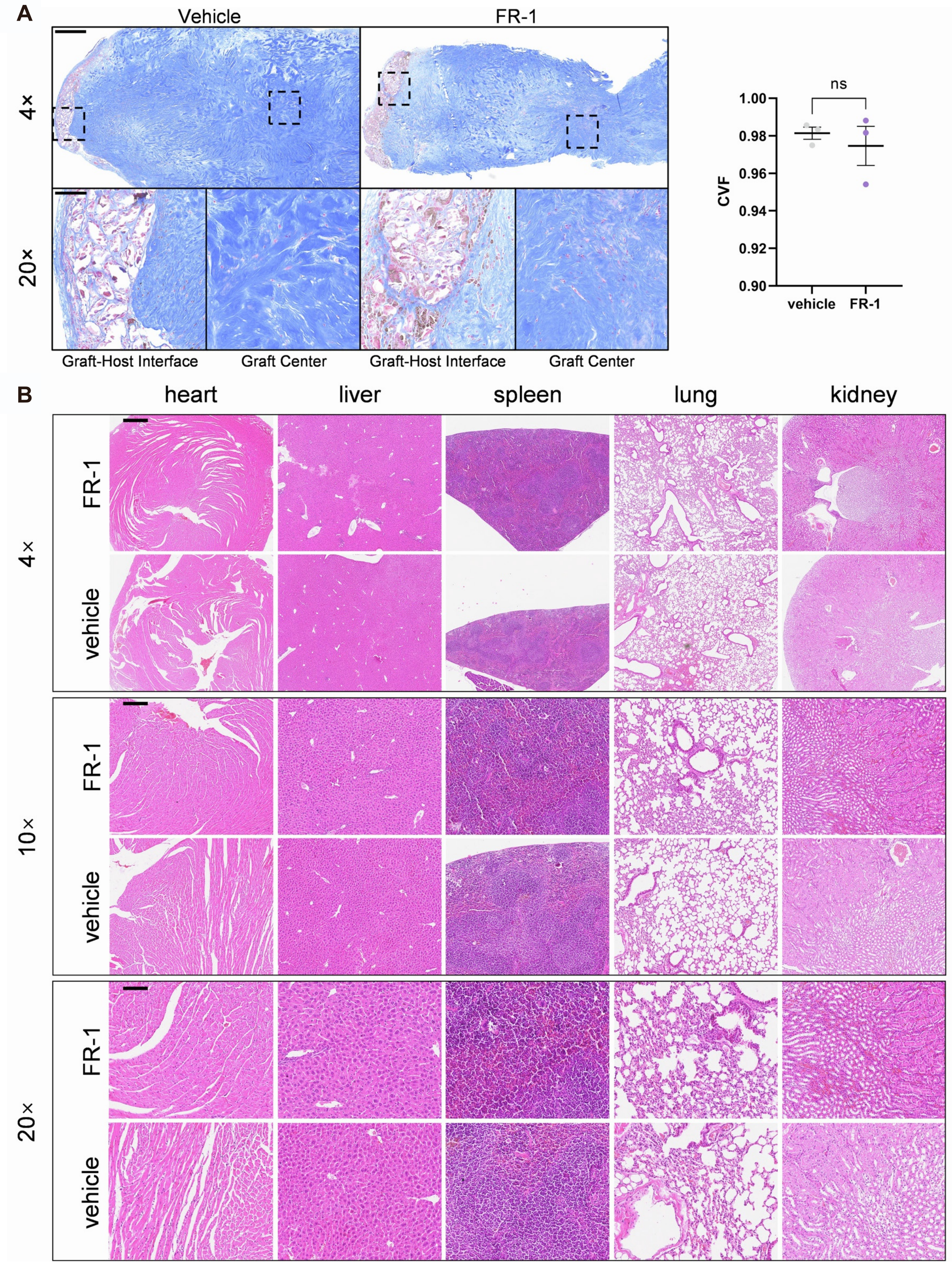

Figure S7. Histopathological analysis of the grafts and major organs from the human keloid patient-derived xenograft (PDX) model, related to Figure 5. (A) Representative Masson's trichrome staining images and corresponding quantification of collagen volume fraction (CVF) of the viable keloid grafts from the vehicle and FR-1 treatment groups at the experimental endpoint (n = 3). Scale bars: 500 μm (4×) and 100 μm (20×). (B) Representative H&E staining images of the heart, liver, spleen, lung, and kidney from healthy mice, topical FR-1-treated mice, and vehicle-treated mice at the experimental endpoint. Scale bars: 500 μm (4×), 100 μm (10×), and 100 μm (20×). Data are represented as mean ± SEM, n represents the number of independent biological replicates, ns, not statistically significant by Student's t test.

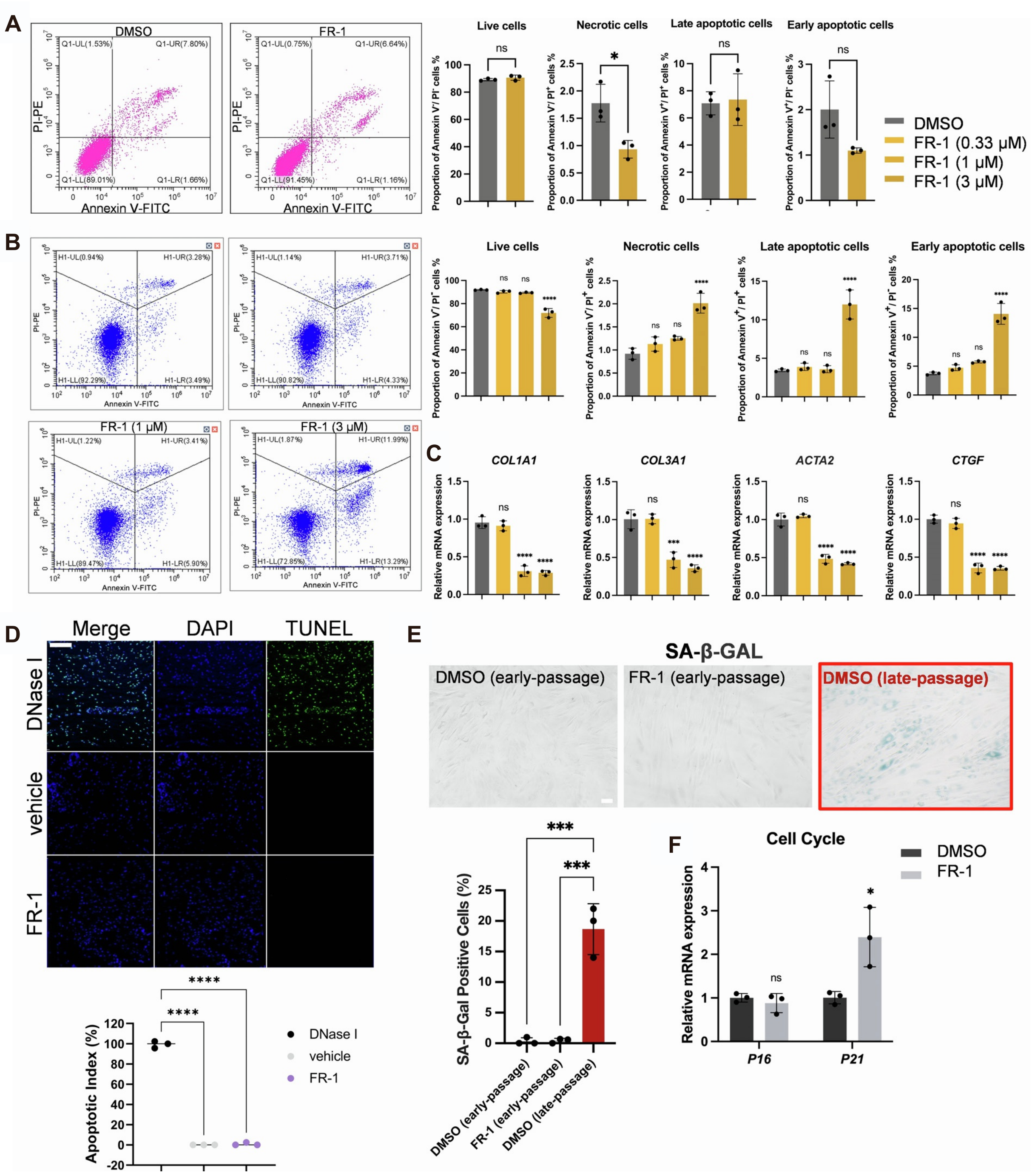

Figure S8. FR-1 reverses fibrosis independent of apoptosis or cellular senescence, related to Figure 6. (A and B) Flow cytometric analysis of apoptosis (Annexin V/PI staining) in keloid fibroblasts treated with DMSO or FR-1 at (A) 3 μM for 48 hours and (B) the indicated concentrations (0.33, 1, and 3 μM) for 120 hours. Representative plots (left) and quantification of apoptotic rates (right) are shown. (C) RT-qPCR quantification of fibrosis-related genes (*COL1A1*, *COL3A1, ACTA2, CTGF*) following 5-day FR-1 treatment. (D) Representative TUNEL staining of scar tissues harvested from the murine linear excision model at the experimental endpoint, following treatment with FR-1 ointment or vehicle. DNase I-treated sections served as a positive control. Scale bar: 100 μm. The apoptotic index is quantified below the representative images. (E) Representative images and quantification of senescence-associated β-galactosidase (SA-β-Gal) staining. Scale bar: 200 μm. (F) RT-qPCR of cell senescence markers (*P16, P21*) in FR-1 and DMSO-treated cells. Data are mean ± SD (n=3), n represents the number of independent biological replicates. $*P < 0.05$; $**P < 0.01$; $***P < 0.001$; $****P < 0.0001$; ns, not statistically significant vs. DMSO by (A, F) Student's t test and (B-E) one-way ANOVA.

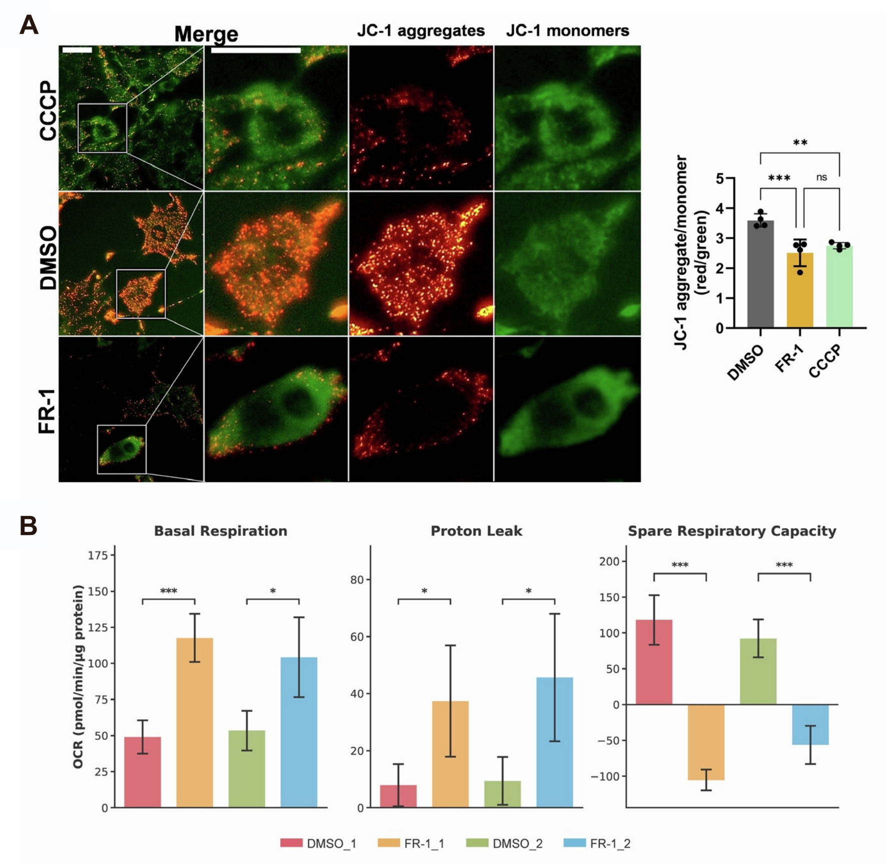

Figure S9. Validation of FR-1 uncoupling activity, related to Figure 6. (A) Representative JC-1 fluorescence images (left) and quantitative analysis of the red/green fluorescence intensity ratio (right) in 3T3-L1 fibroblasts treated with DMSO, FR-1 (1.5 μM), or CCCP (positive control) (n=4). A decrease in the red/green ratio indicates mitochondrial depolarization. Scale bar: 50 μm.
(B) Quantification of proton leak derived from the Seahorse OCR analysis in FR-1- or DMSO-treated fibroblasts (n=4). Data are mean ± SD, n represents the number of independent biological replicates. $**P < 0.01$; $***P < 0.001$; ns, not statistically significant vs. DMSO by one-way ANOVA.

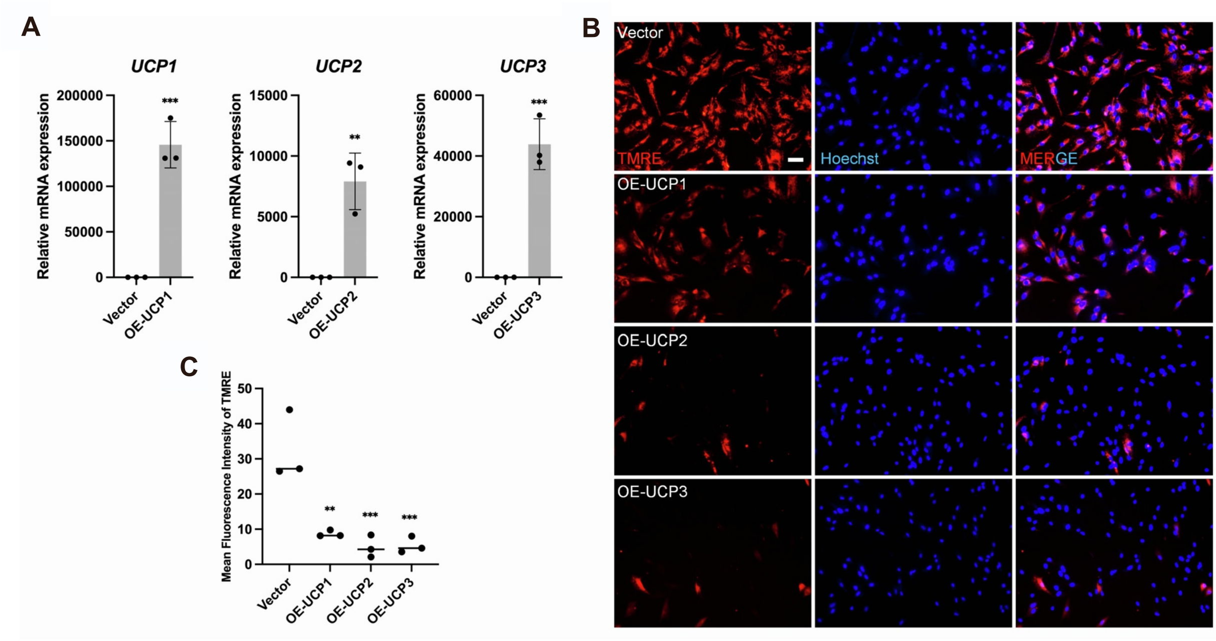

Figure S10. Validation of inducible UCP overexpression and functional mitochondrial depolarization, related to Figure 6. (A) Analysis of *UCP*s overexpression at the mRNA level. (B) Representative image of mitochondrial membrane potential (ΔΨm) changes following UCP overexpression compared to vector control. Scale bar: 100 μm. (C) Quantification of ΔΨm (measured by TMRE) in UCP-overexpressing versus vector control groups. Data are mean ± SD (n=3), n represents the number of independent biological replicates. ∗P < 0.05; ∗∗P < 0.01; ∗∗∗P < 0.001; ∗∗∗∗P < 0.000; ns, not statistically significant vs. vector by one-way ANOVA.

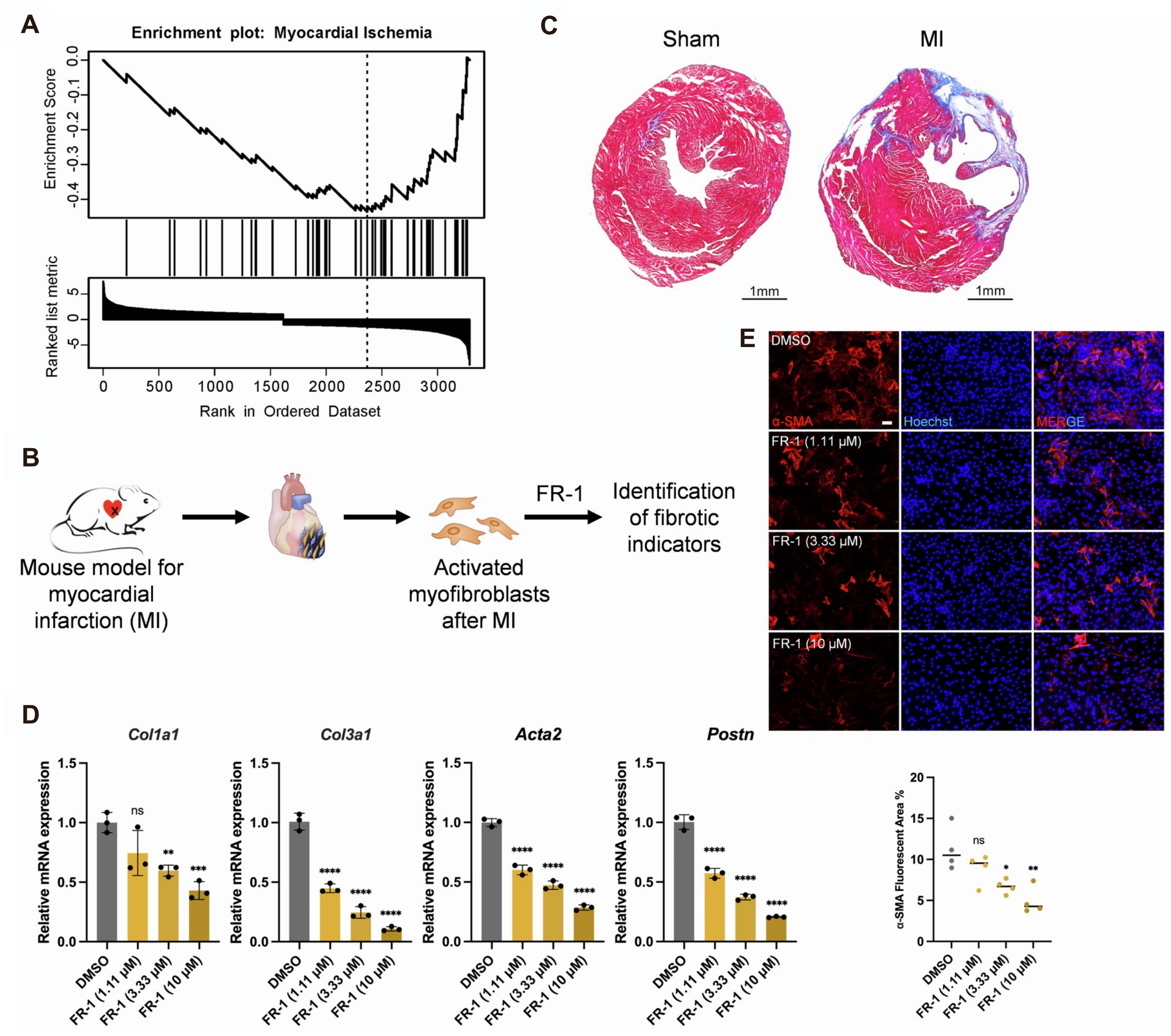

Figure S11. FR-1 attenuates myocardial fibrosis following myocardial infarction (MI), related to Figure 7. (A) GSEA plot depicting downregulation of the DisGeNET "Myocardial Ischemia" gene set in FR-1-treated samples compared to Rottlerin. (B) Schematic illustration of the experimental design. (C) Representative Masson's trichrome staining of cardiac tissue sections from Sham and MI groups, confirming model establishment. Scale bar: 1 mm. (D) RT-qPCR quantification of fibrosis-associated markers in cardiac myofibroblasts treated with FR-1 (n=3). (E) Representative immunofluorescence images (scale bar, 100 μm) and quantification of α-SMA positive area in FR-1-treated myofibroblasts (n=4). Data are mean ± SD, n represents the number of independent biological replicates. $*P < 0.05$; $**P < 0.01$; $***P < 0.001$; $****P < 0.0001$ vs. DMSO by one-way ANOVA.

Table S1. Small molecule list used in chemical screening, related to Figure 1.

Number	Name	Function	Reference
1	Sorafenib	Anti-fibrosis	Hong F et al., 2013[1]
2	Vitamin A	Anti-fibrosis	Murakami K et al., 2011[2]
3	Protocatechuic acid	Anti-fibrosis	Cui B et al., 2021[3]
4	SB431542	Anti-fibrosis	Koh RY et al., 2015[4]
5	Rosiglitazone	Anti-fibrosis	Yu W et al., 2017[5]
6	Nintedanib	Anti-fibrosis	Wuyts WA et al., 2025[6]
7	L-ascorbic acid (Vitamin C)	Anti-fibrosis	Rodrigues da Silva M et al., 2018[7]
8	DB1976	Anti-fibrosis	Wohlfahrt T et al., 2019[8]
9	ICG-001	Anti-fibrosis	Hirakawa T et al., 2019[9]
10	Bosentan	Anti-fibrosis	Clozel M et al., 2005[10]
11	GW788388	Anti-fibrosis	Gellibert F et al., 2006[11]
12	JQ1	Anti-fibrosis	Ijaz et al., 2017[12]
13	Repsox	Anti-fibrosis	Ide M et al., 2017[13]
14	Rottlerin	Anti-fibrosis	Jimenez SA et al., 2001[14]
15	Triamcinolone	Anti-fibrosis	Carroll LA et al., 2002[15]
16	bFGF	Anti-fibrosis	Eto et al., 2012[16]
17	Imatinib	Anti-fibrosis	Distler JH et al., 2007[17]
18	CAY10585	Anti-fibrosis	Kang Y et al., 2020[18]
19	5-FU	Anti-fibrosis	Wendling J et al., 2003[19]
20	SAR-100842	Anti-fibrosis	Ledein L et al., 2020[20]
21	AD*	Adipogenesis	Plikus MV et al., 2017[21]
22	AD*+MSC2530818	Adipogenesis	Li J et al., 2023[22]
23	AD*+Repsox	Adipogenesis	Li J et al., 2023[22]

AD*: Adipocyte Differentiation (AD) medium (DMEM/high glucose, 1% ITS, 0.5mM isobutylmethylxanthine, 0.1 μM cortisol, 1 μM dexamethasone, 0.2 nM triiodothyronine, and 1 μM rosiglitazone)

Table S2. Patient baseline characteristics, related to Figure 1 and 2.

Patient ID	Gender	Age/year	Scar location	Pathological properties
1	male	70	Shoulder	Keloid
2	male	43	Thorax	Keloid
3	female	25	Dorsum	Keloid
4	male	30	Nucha	Hypertrophic scar
5	male	8	Shoulder	Hypertrophic scar
6	female	42	Axilla	Hypertrophic scar
7	male	24	Dorsum	Keloid
8	male	44	Dorsum	Keloid
9	male	47	Dorsum	Keloid
10	male	23	Thorax	Keloid

Table S3. Physicochemical properties and *in vitro* ADME profiles of Rottlerin and FR-1, related to Figure 2.

Pharmaceutical properties		Rottlerin	FR-1
Log*P*	(pH=7.4)	2.11	2.24
Solubility	PBS (pH 7.4, μM)	0.006	1.79
Solubility	Thermodynamic (EtOH, μM)		1973
Caco-2	AB; BA (*1E-6 cm/s); ER	<0.002; 0.03; >20.21	<0.001; 0.02; >24.97
PPB	(m/r/d/c/h bound%)	NA	NA
CYP450 inhibition	3A4–M; 3A4–T; 2C9; 1A2; 2C19; 2D6 (μM)	13.56; 11.06; 2.18; >30; 11.39; >30	6.76; 4.56; 4.22; >30; 9.82; >30
Hepatocyte Cl_{int}	(m/r/d/c/h, μL/min/10e6 cell)	-/-/-/-/101	-/-/-/137/94
Liver microsome stability	(m/r/d/c/h, μL/min/mg protein)	12/<10/<10/<10/<10	66/14/<10/<10/<10

Table S4. List of genes in the IL-17 and TNF signaling pathways significantly downregulated by FR-1 compared to Rottlerin, related to Figure 2.

	gene	baseMean	log2FoldChange	lfcSE	stat	pvalue	padj
IL-17 signaling pathway	S100A7	44.5087239	-7.5862814	1.5548636	-4.8790655	1.07E-06	6.61E-06
	CXCL10	20.9571212	-4.8822574	1.17256935	-4.163726	3.13E-05	0.00014341
	FOSB	633.677965	-4.7532318	0.27664956	-17.181418	3.66E-66	2.41E-63
	CCL20	22.2659731	-4.5358265	1.07069883	-4.2363234	2.27E-05	0.00010718
	FOS	253.288203	-3.5330001	0.3334544	-10.595152	3.14E-26	2.04E-24
	NFKBIA	6072.94225	-3.4625823	0.18491155	-18.725614	3.06E-78	4.03E-75
	IL17RE	61.1220693	-2.4614057	0.56488108	-4.3573875	1.32E-05	6.57E-05
	CXCL3	1377.06352	-1.9858448	0.19380948	-10.246376	1.23E-24	6.85E-23
	CEBPB	12364.5634	-1.718274	0.18412157	-9.3322799	1.04E-20	3.92E-19
	TNFAIP3	2443.40998	-1.7142342	0.18516531	-9.2578581	2.09E-20	7.60E-19
	PTGS2	2852.24613	-1.6127782	0.17094951	-9.4342371	3.94E-21	1.57E-19
	IL1B	107.609993	-1.5725625	0.43873138	-3.5843401	0.00033793	0.00123919
	TRAF4	1048.84018	-1.4927844	0.19901842	-7.5007347	6.35E-14	1.15E-12
	CASP3	3512.12866	-1.3924707	0.16820997	-8.27817	1.25E-16	3.06E-15
	CXCL1	703.631099	-1.2347801	0.23185365	-5.3256877	1.01E-07	7.54E-07
	CXCL5	232.946341	-1.1526656	0.29354807	-3.9266671	8.61E-05	0.00036145
	JUND	4037.33619	-1.1490991	0.20747818	-5.5384092	3.05E-08	2.49E-07
	CXCL2	1711	-1.1199794	0.21335134	-5.24946	1.53E-07	1.11E-06
	NFKB1	2179.80823	-1.1041012	0.18603403	-5.9349419	2.94E-09	2.80E-08
	MMP3	1698.76776	-1.094812	0.23048137	-4.7501106	2.03E-06	1.19E-05
TNF signaling pathway	CXCL10	20.9571212	-4.8822574	1.17256935	-4.163726	3.13E-05	0.00014341
	CCL20	22.2659731	-4.5358265	1.07069883	-4.2363234	2.27E-05	0.00010718
	EDN1	128.624042	-4.194698	0.44802208	-9.3627038	7.77E-21	2.97E-19
	FOS	253.288203	-3.5330001	0.3334544	-10.595152	3.14E-26	2.04E-24
	JUNB	3432.33032	-3.4635734	0.19390125	-17.862564	2.31E-71	1.82E-68
	NFKBIA	6072.94225	-3.4625823	0.18491155	-18.725614	3.06E-78	4.03E-75
	MAP3K8	469.506697	-3.3913276	0.26126905	-12.980212	1.58E-38	2.58E-36
	JAG1	1164.50326	-2.6995474	0.21663598	-12.461214	1.22E-35	1.65E-33
	LIF	621.524807	-2.5766297	0.24118745	-10.6831	1.22E-26	8.38E-25
	NOD2	69.4776428	-2.3287552	0.51244122	-4.5444339	5.51E-06	2.97E-05
	SOCS3	892.957563	-2.231285	0.21093914	-10.577861	3.77E-26	2.44E-24
	ICAM1	1846.37085	-2.2040752	0.1898566	-11.609157	3.70E-31	3.52E-29
	MAP2K3	4384.72628	-2.0354893	0.16926399	-12.025531	2.61E-33	3.05E-31
	CXCL3	1377.06352	-1.9858448	0.19380948	-10.246376	1.23E-24	6.85E-23
	CEBPB	12364.5634	-1.718274	0.18412157	-9.3322799	1.04E-20	3.92E-19
	TNFAIP3	2443.40998	-1.7142342	0.18516531	-9.2578581	2.09E-20	7.60E-19
	BCL3	611.346371	-1.7034416	0.22676915	-7.511787	5.83E-14	1.07E-12
	DAB2IP	1613.26437	-1.6270082	0.2220823	-7.3261498	2.37E-13	3.98E-12
	PTGS2	2852.24613	-1.6127782	0.17094951	-9.4342371	3.94E-21	1.57E-19
	BIRC3	1516.32705	-1.579756	0.20057545	-7.8761183	3.38E-15	7.23E-14
	IL1B	107.609993	-1.5725625	0.43873138	-3.5843401	0.00033793	0.00123919
	PIK3R3	350.413681	-1.567037	0.2987538	-5.2452453	1.56E-07	1.13E-06

IRF1	3903.84613	-1.4963101	0.17043036	-8.7795983	1.64E-18	4.95E-17
RHBDF2	352.498134	-1.4194575	0.27703578	-5.1237335	3.00E-07	2.06E-06
CASP3	3512.12866	-1.3924707	0.16820997	-8.27817	1.25E-16	3.06E-15
CXCL1	703.631099	-1.2347801	0.23185365	-5.3256877	1.01E-07	7.54E-07
MAP2K1	3713.39438	-1.2217254	0.1690846	-7.2255278	4.99E-13	8.15E-12
CXCL5	232.946341	-1.1526656	0.29354807	-3.9266671	8.61E-05	0.00036145
CREB3L1	4410.53481	-1.1458371	0.1715795	-6.6781702	2.42E-11	3.13E-10
CXCL2	1711	-1.1199794	0.21335134	-5.24946	1.53E-07	1.11E-06
NFKB1	2179.80823	-1.1041012	0.18603403	-5.9349419	2.94E-09	2.80E-08
MMP3	1698.76776	-1.094812	0.23048137	-4.7501106	2.03E-06	1.19E-05
MLKL	196.794846	-1.0930032	0.31203793	-3.5027896	0.00046041	0.00163512
MMP14	94307.5748	-1.0929128	0.17930697	-6.0952055	1.09E-09	1.10E-08

Table S5. Mass balance and cutaneous biodistribution of FR-1, related to Figure 3.

Compartment	Drug Content (μg) [Mean ± SD]	Percentage of Dose (%)
Surface Residue	3126.5 ± 142.3	98.4 ± 0.5
Stratum Corneum	1.02 ± 0.33	0.03 ± 0.01
Epidermis	1.30 ± 0.49	0.04 ± 0.01
Dermis	1.81 ± 0.13*	0.06 ± 0.01
Receptor Fluid	N.D.	0
Total Recovery	3130.6 ± 145.2	101.4 ± 2.1

Notes:
Data are presented as mean ± SD (n=3), n represents the number of independent skin donors (biological replicates).
N.D.: Not Detected.
*The tissue concentration in the dermis was calculated to be 2.88 ± 0.32 μg/g.

Table S6. Inhibitory activities of PKC inhibitors, related to Figure 6.

Small Molecules	Protein Kinase C Family	IC50	Reference
Rottlerin	PKCα	30-42 μM	Gschwendt M et al., 1994[23]. María Rosa López-Huertas et al., 2011[24].
	PKCβ	30-42 μM	
	PKCγ	30-42 μM	
	PKCδ	3-6 μM	
	PKCη	80-100 μM	
	PKCε	80-100 μM	
	PKCζ	80-100 μM	
GO6983	PKCα	7 nM	Gschwendt M et al., 1996[25].
	PKCβ	7 nM	
	PKCγ	6 nM	
	PKCδ	10 nM	
	PKCη	/	
	PKCε	/	
	PKCζ	60 nM	
Sotrastaurin	PKCα	0.95 nM	Evenou JP et al., 2009[26].
	PKCβ	0.64 nM	
	PKCγ	/	
	PKCδ	2.1 nM	
	PKCη	1.8 nM	
	PKCε	3.2 nM	
	PKCζ	/	

Table S7. Specific arrangement of small molecules and corresponding barcodes, related to METHOD DETAILS.

Plate	1	2	3	4	5	6	7	8	9	10	11	12
A	DMSO	DB1976	Imatinib	Nintedanib	DMSO	DB1976	Imatinib	Nintedanib	DMSO	DB1976	Imatinib	Nintedanib
B	DMSO	JQ1	Rosiglitazone	TGFβ1	DMSO	JQ1	Rosiglitazone	TGFβ1	DMSO	JQ1	Rosiglitazone	TGFβ1
C	DMSO	Repsox	CAY10585	AD	DMSO	Repsox	CAY10585	AD	DMSO	Repsox	CAY10585	AD
D	DMSO	SB431542	ICG-001	AD+MSC2530818	DMSO	SB431542	ICG-001	AD+MSC2530818	DMSO	SB431542	ICG-001	AD+MSC2530818
E	DMSO	Rottlerin	5-FU	AD+Repsox	DMSO	Rottlerin	5-FU	AD+Repsox	DMSO	Rottlerin	5-FU	AD+Repsox
F	DMSO	Sorafenib	SAR-100842	L-ascorbic acid	DMSO	Sorafenib	SAR-100842	L-ascorbic acid	DMSO	Sorafenib	SAR-100842	L-ascorbic acid
G	DMSO	Triamcinolone	Bosentan	Vitamin A (retinol)	DMSO	Triamcinolone	Bosentan	Vitamin A (retinol)	DMSO	Triamcinolone	Bosentan	Vitamin A (retinol)
H	DMSO	bFGF	GW788388	Protocatechuic acid	DMSO	bFGF	GW788388	Protocatechuic acid	DMSO	bFGF	GW788388	Protocatechuic acid

well_position	Barcode	well_position	Barcode	well_position	Barcode
A1	AACAAGGTAC	C9	AATCCTACCA	F5	ACGCCTTCGT
A2	AACAATCAGG	C10	AATCGTCCGC	F6	ACGCTGGATA
A3	AACATGGAGA	C11	AATGAGAGCA	F7	ACGTGCTGAT
A4	AACATTACCG	C12	AATGTCAGTG	F8	ACTCCAAGCC
A5	AACCGCGACT	D1	ACAACAGTCG	F9	ACTTAACTGC
A6	AACCGGAAGG	D2	ACAACCATAC	F10	ACTTCATCAC
A7	AACCTCATAG	D3	ACACAATCTC	F11	ACTTGAGGAA
A8	AACGTAAGCT	D4	ACACAGTGAA	F12	ACTTGTAAGG
A9	AAGACGGATT	D5	ACACGGTCCT	G1	AGACCGTTAT
A10	AAGATCGGCG	D6	ACACTTGCTG	G2	AGACTAGCAT
A11	AAGCGATGTT	D7	ACCAGGACCA	G3	AGAGTGTAAC
A12	AAGCGTTCAG	D8	ACCATAACAC	G4	AGAGTTCTGC
B1	AAGGTCTGGA	D9	ACCGGTACAG	G5	AGCATGTCAT
B2	AAGTTAGCGC	D10	ACCGTACTTC	G6	AGCCACTAGC
B3	AATAGCCACA	D11	ACCTGTCCGA	G7	AGCGATAACG

B4	AATCACGCGA	D12	ACCTTATGTG	G8	AGCGTACAAT
B5	AACACCTAGT	E1	AATGAACACG	G9	ACGGTCCGTT
B6	AACAGGCAAT	E2	AATGACCTTC	G10	ACGTAGGCAC
B7	AACCAGCCAG	E3	AATTAGGCCG	G11	ACTGGCGCAT
B8	AACCAGTTGA	E4	AATTGCGATG	G12	ACTGGCTTCC
B9	AACCGGCGTA	E5	ACAACGGAGC	H1	ACTTCGTTGA
B10	AACCTAGTCC	E6	ACAAGCGCGA	H2	ACTTCTCCTG
B11	AACTCTACAC	E7	ACACCGAATT	H3	AGAACCACGG
B12	AACTGTGTCA	E8	ACACGCAGTA	H4	AGAAGCAATC
C1	AAGATGTCCA	E9	ACAGTGCCAA	H5	AGAGATGCAG
C2	AAGCATATGG	E10	ACATGTGTGC	H6	AGAGCTTACA
C3	AAGCTCACCT	E11	ACCGAACCGT	H7	AGATAGTGCT
C4	AAGGCATGCG	E12	ACCGAGAGTC	H8	AGCAATGCGC
C5	AAGTTCCTTG	F1	ACCTCCGACA	H9	AGCCAGAATA
C6	AATACCGGTA	F2	ACCTCTCTCC	H10	AGCCAGCTCT
C7	AATCCATCTG	F3	ACGAATGACA	H11	AGCTATTCCA
C8	AATCCGCTCC	F4	ACGCCTCAAC	H12	AGCTCCTCAG

SUPPLEMENTAL REFERENCES

1. Hong, F., Chou, H., Fiel, M.I., and Friedman, S.L. (2013). Antifibrotic activity of sorafenib in experimental hepatic fibrosis: refinement of inhibitory targets, dosing, and window of efficacy in vivo. Dig Dis Sci *58*, 257-264. 10.1007/s10620-012-2325-y.
2. Murakami, K., Kaji, T., Shimono, R., Hayashida, Y., Matsufuji, H., Tsuyama, S., Maezono, R., Kosai, K., and Takamatsu, H. (2011). Therapeutic effects of vitamin A on experimental cholestatic rats with hepatic fibrosis. Pediatr Surg Int *27*, 863-870. 10.1007/s00383-011-2853-0.
3. Cui, B., Yang, Z., Wang, S., Guo, M., Li, Q., Zhang, Q., and Bi, X. (2021). The protective role of protocatechuic acid against chemically induced liver fibrosis in vitro and in vivo. Pharmazie *76*, 232-238. 10.1691/ph.2021.0909.
4. Koh, R.Y., Lim, C.L., Uhal, B.D., Abdullah, M., Vidyadaran, S., Ho, C.C., and Seow, H.F. (2015). Inhibition of transforming growth factor-β via the activin receptor-like kinase-5 inhibitor attenuates pulmonary fibrosis. Mol Med Rep *11*, 3808-3813. 10.3892/mmr.2015.3193.
5. Yu, W., Mi, L., and Long, T. (2017). Efficacies of rosiglitazone and retinoin on bleomycin-induced pulmonary fibrosis in rats. Exp Ther Med *14*, 609-615. 10.3892/etm.2017.4555.
6. Wuyts, W.A., Bonella, F., Chaudhuri, N., Varone, F., Antin-Ozerkis, D., Song, J.W., Miede, C., Dumistracel, M., Coeck, C., and Cottin, V. (2025). Continued Treatment with Nintedanib in Patients with Progressive Pulmonary Fibrosis: Data from INBUILD-ON. Lung *203*, 25. 10.1007/s00408-024-00778-z.
7. Rodrigues da Silva, M., Schapochnik, A., Peres Leal, M., Esteves, J., Bichels Hebeda, C., Sandri, S., Pavani, C., Ratto Tempestini Horliana, A.C., Farsky, S.H.P., and Lino-Dos-Santos-Franco, A. (2018). Beneficial effects of ascorbic acid to treat lung fibrosis induced by paraquat. PLoS One *13*, e0205535. 10.1371/journal.pone.0205535.
8. Wohlfahrt, T., Rauber, S., Uebe, S., Luber, M., Soare, A., Ekici, A., Weber, S., Matei, A.E., Chen, C.W., Maier, C., et al. (2019). PU.1 controls fibroblast polarization and tissue fibrosis. Nature *566*, 344-349. 10.1038/s41586-019-0896-x.
9. Hirakawa, T., Nasu, K., Miyabe, S., Kouji, H., Katoh, A., Uemura, N., and Narahara, H. (2019). β-catenin signaling inhibitors ICG-001 and C-82 improve fibrosis in preclinical models of endometriosis. Sci Rep *9*, 20056. 10.1038/s41598-019-56302-4.
10. Clozel, M., and Salloukh, H. (2005). Role of endothelin in fibrosis and anti-fibrotic potential of bosentan. Ann Med *37*, 2-12. 10.1080/07853890410018925.
11. Gellibert, F., de Gouville, A.C., Woolven, J., Mathews, N., Nguyen, V.L., Bertho-Ruault, C., Patikis, A., Grygielko, E.T., Laping, N.J., and Huet, S. (2006). Discovery of 4-{4-[3-(pyridin-2-yl)-1H-pyrazol-4-yl]pyridin-2-yl}-N-(tetrahydro-2H- pyran-4-yl)benzamide (GW788388): a potent, selective, and orally active transforming growth factor-beta type I receptor inhibitor. J Med Chem *49*, 2210-2221. 10.1021/jm0509905.
12. Ijaz, T., Jamaluddin, M., Zhao, Y., Zhang, Y., Jay, J., Finnerty, C.C., Herndon, D.N., Tilton, R.G., and Brasier, A.R. (2017). Coordinate activities of BRD4 and CDK9 in the transcriptional elongation complex are required for TGFbeta-induced Nox4 expression and myofibroblast transdifferentiation. Cell Death Dis *8*, e2606. 10.1038/cddis.2016.434.
13. Ide, M., Jinnin, M., Tomizawa, Y., Wang, Z., Kajihara, I., Fukushima, S., Hashizume, Y., Asano, Y., and Ihn, H. (2017). Transforming growth factor β-inhibitor Repsox downregulates collagen expression of scleroderma dermal fibroblasts and prevents bleomycin-induced mice skin fibrosis. Exp Dermatol *26*, 1139-1143. 10.1111/exd.13366.
14. Jimenez, S.A., Gaidarova, S., Saitta, B., Sandorfi, N., Herrich, D.J., Rosenbloom, J.C., Kucich, U., Abrams, W.R., and Rosenbloom, J. (2001). Role of protein kinase C-delta in the regulation of collagen gene expression in scleroderma fibroblasts. J Clin Invest *108*, 1395-1403. 10.1172/jci12347.
15. Carroll, L.A., Hanasono, M.M., Mikulec, A.A., Kita, M., and Koch, R.J. (2002). Triamcinolone stimulates bFGF production and inhibits TGF-beta1 production by human dermal fibroblasts. Dermatol Surg *28*, 704-709. 10.1046/j.1524-4725.2002.02012.x.
16. Eto, H., Suga, H., Aoi, N., Kato, H., Doi, K., Kuno, S., Tabata, Y., and Yoshimura, K. (2012). Therapeutic potential of fibroblast growth factor-2 for hypertrophic scars: upregulation of MMP-1 and HGF expression. Lab Invest *92*, 214-223. 10.1038/labinvest.2011.127.
17. Distler, J.H., Jüngel, A., Huber, L.C., Schulze-Horsel, U., Zwerina, J., Gay, R.E., Michel, B.A., Hauser, T., Schett, G., Gay, S., and Distler, O. (2007). Imatinib mesylate reduces production of extracellular matrix and prevents development of experimental dermal fibrosis. Arthritis Rheum *56*, 311-322. 10.1002/art.22314.

18. Kang, Y., Roh, M.R., Rajadurai, S., Rajadurai, A., Kumar, R., Njauw, C.N., Zheng, Z., and Tsao, H. (2020). Hypoxia and HIF-1α Regulate Collagen Production in Keloids. J Invest Dermatol *140*, 2157-2165. 10.1016/j.jid.2020.01.036.
19. Wendling, J., Marchand, A., Mauviel, A., and Verrecchia, F. (2003). 5-fluorouracil blocks transforming growth factor-beta-induced alpha 2 type I collagen gene (COL1A2) expression in human fibroblasts via c-Jun NH2-terminal kinase/activator protein-1 activation. Mol Pharmacol *64*, 707-713. 10.1124/mol.64.3.707.
20. Ledein, L., Léger, B., Dees, C., Beyer, C., Distler, A., Vettori, S., Boukaiba, R., Bidouard, J.P., Schaefer, M., Pernerstorfer, J., et al. (2020). Translational engagement of lysophosphatidic acid receptor 1 in skin fibrosis: from dermal fibroblasts of patients with scleroderma to tight skin 1 mouse. Br J Pharmacol *177*, 4296-4309. 10.1111/bph.15190.
21. Plikus, M.V., Guerrero-Juarez, C.F., Ito, M., Li, Y.R., Dedhia, P.H., Zheng, Y., Shao, M., Gay, D.L., Ramos, R., Hsi, T.C., et al. (2017). Regeneration of fat cells from myofibroblasts during wound healing. Science *355*, 748-752. 10.1126/science.aai8792.
22. Li, J., Bai, Y., Liu, Y., Song, Z., Yang, Y., and Zhao, Y. (2023). Transcriptome-based chemical screens identify CDK8 as a common barrier in multiple cell reprogramming systems. Cell Rep *42*, 112566. 10.1016/j.celrep.2023.112566.
23. Gschwendt, M., Müller, H.J., Kielbassa, K., Zang, R., Kittstein, W., Rincke, G., and Marks, F. (1994). Rottlerin, a novel protein kinase inhibitor. Biochem Biophys Res Commun *199*, 93-98. 10.1006/bbrc.1994.1199.
24. López-Huertas, M.R., Mateos, E., Díaz-Gil, G., Gómez-Esquer, F., Sánchez del Cojo, M., Alcamí, J., and Coiras, M. (2011). Protein kinase Ctheta is a specific target for inhibition of the HIV type 1 replication in CD4+ T lymphocytes. J Biol Chem *286*, 27363-27377. 10.1074/jbc.M110.210443.
25. Gschwendt, M., Dieterich, S., Rennecke, J., Kittstein, W., Mueller, H.J., and Johannes, F.J. (1996). Inhibition of protein kinase C mu by various inhibitors. Differentiation from protein kinase c isoenzymes. FEBS Lett *392*, 77-80. 10.1016/0014-5793(96)00785-5.
26. Evenou, J.P., Wagner, J., Zenke, G., Brinkmann, V., Wagner, K., Kovarik, J., Welzenbach, K.A., Weitz-Schmidt, G., Guntermann, C., Towbin, H., et al. (2009). The potent protein kinase C-selective inhibitor AEB071 (sotrastaurin) represents a new class of immunosuppressive agents affecting early T-cell activation. J Pharmacol Exp Ther *330*, 792-801. 10.1124/jpet.109.153205.
