# Supplementary material for: Transcriptome-guided development of a fibrosis-reversal compound reduces skin scarring and allows regeneration via mitochondrial uncoupling
Source: Cell Rep Med. 2026 May 19;7(5):102821. doi: 10.1016/j.xcrm.2026.102821 (PMC13198262; doi:10.1016/j.xcrm.2026.102821)
Supplement: Document S2. Article plus supplemental information [file mmc2.pdf]

Cell Reports
Medicine

Article

Transcriptome-guided development of a fibrosis-reversal compound reduces skin scarring and allows regeneration via mitochondrial uncoupling

Graphical abstract

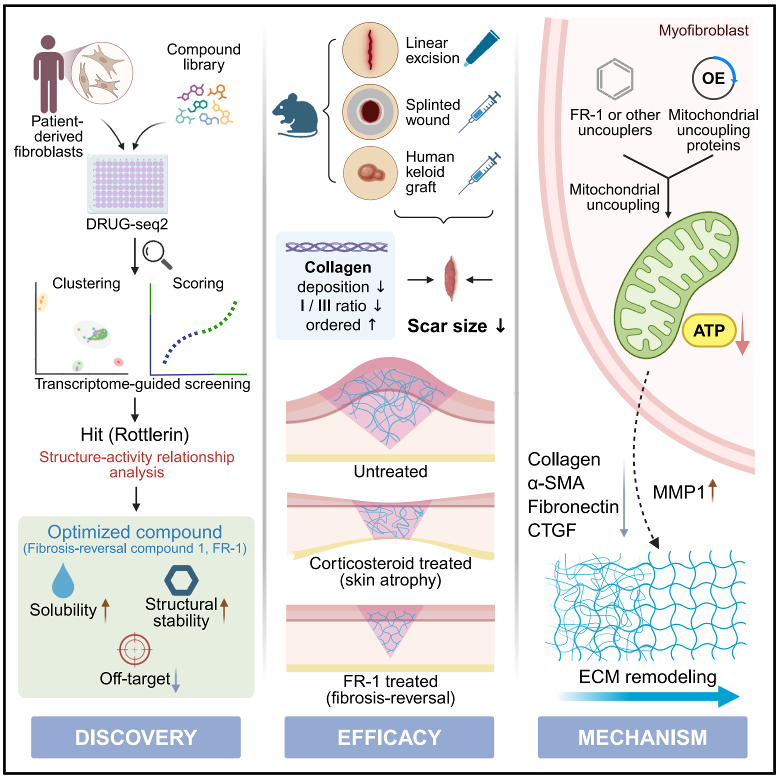

Highlights

- Transcriptome-guided screening and optimization yield a fibrosis-reversal compound
- The compound reverses the pathological fibrotic phenotype *in vitro*
- The compound reduces scars topically and promotes wound regeneration *in vivo*
- Mitochondrial uncoupling drives the fibrosis-reversal activity of the compound

Authors

Chun-Ye Chen, Ruilin Xu, Mingguang Mo, ..., Yong Yang, Wei-Qiang Tan, Yang Zhao

Correspondence

bihongsen@bjmu.edu.cn (H.B.), yyang@pumcderm.cams.cn (Y.Y.), tanweixxxx@zju.edu.cn (W.-Q.T.), yangzhao@pku.edu.cn (Y.Z.)

In brief

Given the lack of effective treatments for pathological scarring, Chen et al. present a fibrosis-reversal compound (FR-1) discovered through transcriptome-guided drug discovery. By mitochondrial uncoupling, this compound effectively reverses the fibrotic phenotype in both cellular and animal models, offering a promising therapeutic strategy, especially for topical attenuation skin scarring.

Chen et al., 2026, Cell Reports Medicine *7*, 102821
May 19, 2026 © 2026 The Authors. Published by Elsevier Inc.
https://doi.org/10.1016/j.xcrm.2026.102821

CellPress

Cell Reports Medicine

CellPress
OPEN ACCESS

Article

Transcriptome-guided development of a fibrosis-reversal compound reduces skin scarring and allows regeneration via mitochondrial uncoupling

Chun-Ye Chen,[1,8] Ruilin Xu,[2,8] Mingguang Mo,[3] Jiahao Wu,[3] Jun Chi,[3] Zhang-Rui Wu,[1] Yi Wang,[1] Xin-Cao Zhong,[1] Xiao-Ying Lin,[1] Yang Liu,[3] Jingdong Wu,[2] Huaan Fang,[4] Hongli Jia,[5] Hongsen Bi,[4,*] Yong Yang,[6,*] Wei-Qiang Tan,[1,*] and Yang Zhao[2,7,9,*]

[1]Department of Plastic Surgery, Sir Run Run Shaw Hospital, Zhejiang University School of Medicine, Hangzhou 310016, China
[2]Peking-Tsinghua Center for Life Sciences, Academy for Advanced Interdisciplinary Studies, State Key Laboratory of Natural and Biomimetic Drugs, Ministry of Education Key Laboratory of Cell Proliferation and Differentiation, Beijing Advanced Center of Cellular Homeostasis and Aging-Related Diseases, Institute of Advanced Clinical Medicine, Peking University, Beijing 100871, China
[3]Plastech Pharmaceutical Technology Co., Ltd, Nanjing 210043, China
[4]Department of Plastic Surgery, Peking University Third Hospital, Beijing 100191, China
[5]State Key Laboratory of Natural and Biomimetic Drugs, School of Pharmaceutical Sciences, Peking University, Beijing 100191, China
[6]Jiangsu Key Laboratory of Molecular Biology for Skin Diseases and STIs, Institute of Dermatology, Chinese Academy of Medical Sciences and Peking Union Medical College, Nanjing 210042, China
[7]Senior author
[8]These authors contributed equally
[9]Lead contact
*Correspondence: bihongsen@bjmu.edu.cn (H.B.), yyang@pumcderm.cams.cn (Y.Y.), tanweixxxx@zju.edu.cn (W.-Q.T.), yangzhao@pku.edu.cn (Y.Z.)
https://doi.org/10.1016/j.xcrm.2026.102821

SUMMARY

Skin scarring impairs function and aesthetics. Current therapies show limited efficacy and cause iatrogenic dermal disruption (e.g., triamcinolone acetonide [TA], a first-line corticosteroid for keloids), with topical medications demonstrating inferior outcomes. Through transcriptome-guided evaluation, we develop FR-1 (fibrosis-reversal compound 1), a small molecule that reverses fibrosis *in vitro* by inhibiting fibroblast proliferation, suppressing α-smooth muscle actin (α-SMA), and remodeling the extracellular matrix (ECM) via collagen downregulation and matrix metalloproteinase-1 (MMP1) induction. In a murine linear excisional wound model, topical FR-1 application reduces scar area. Notably, unlike TA, FR-1 avoids skin atrophy and hair follicle damage. Comprehensive safety evaluations, druggability and skin permeation assessments, and studies utilizing patient-derived keloid *ex vivo* explants and *in vivo* xenografts demonstrate its translational potential. Mechanistically, FR-1 induces mitochondrial uncoupling, lowering ATP levels in profibrotic myofibroblasts. Other uncouplers similarly attenuate fibrosis. This work identifies a topical small molecule that attenuates scarring with translational potential, highlighting the therapeutic potential of mitochondrial uncouplers in resolving fibrosis.

INTRODUCTION

Fibrosis, a disordered tissue repair process following injury, affects vital organs, including the skin, heart, lungs, and kidneys, and is statistically responsible for 45% of deaths in industrialized countries.[1] For the largest organ in the human body, the skin, wounds usually heal by forming fibrotic scars. This results from rapid fibrous tissue growth that outpaces normal skin regeneration, which can quickly seal wounds. While this rapid "welding" process prevents microbial infection and fatal bleeding, it compromises the skin's structure and function. Unlike normal skin, scar tissue lacks skin appendages such as hair follicles and sweat glands, as well as biomechanical resilience.[1–5] Pathological scarring, such as hypertrophic scars and keloid lesions, exacerbates this burden, causing functional deficits, aesthetic damage, and psychological distress, culminating in substantial healthcare and economic burdens globally.[5]

Current scar therapies—such as surgery, corticosteroids, laser, radiation, and pressure therapy—are limited by adverse effects and recurrence risks.[5–7] For example, intralesional injection of triamcinolone acetonide (TA), a first-line corticosteroid medication for keloids and hypertrophic scars, reduces keloid size but induces skin atrophy, telangiectasias, and steroid dermatitis.[8,9] Even topical TA retains safety risks with diminished efficacy. This necessitates the development of promising agents that achieve: (1) suppression of pathological fibroblast progression, (2) amelioration of dysregulated extracellular matrix (ECM)

© 2026 The Authors. Published by Elsevier Inc.
This is an open access article under the CC BY license (http://creativecommons.org/licenses/by/4.0/).

deposition, and (3) enhanced clinical translatability with minimal off-target effects.

Pathological scarring arises from sustained fibroblast activation and dysregulated ECM remodeling, a self-perpetuating process that disrupts tissue homeostasis.[1,3,5] Human dermal fibroblasts are the primary effector cells in cutaneous fibrosis, driving excessive ECM deposition and contraction. *In vitro* cultures of scar-derived fibroblasts retain key pathological features of fibrotic niches, including elevated collagen synthesis, α-smooth muscle actin (α-SMA, encoded by *ACTA2*) expression, and transforming growth factor β (TGF-β) responsiveness, making them a clinically relevant model for screening anti-fibrotic compounds.[10,11] Other critical pathways involved in fibrosis include the vascular endothelial growth factor receptor (VEGFR) family, platelet-derived growth factor receptor beta (PDGFRβ), fibroblast growth factor receptor (FGFR), and wingless-type MMTV integration site family (Wnt)/β-catenin signaling. Mechanosensing (e.g., Yes-associated protein/transcriptional coactivator with PSD-95/Dlg/ZO-1 [PDZ]-binding motif [YAP/TAZ] signaling) and unresolved inflammation also drive fibroblast activation and ECM deposition.[12,13] Targeting these pathways attenuates fibrosis in preclinical models.[1,5] However, current therapeutic strategies only partially disrupt fibrotic signaling and fail to address the persistent pathological activation state of fibroblasts themselves.[14]

Cellular phenotypes exhibit inherent plasticity and can be modulated using transcription factors or small molecules, suggesting that the persistent pathological activation of fibroblasts is amenable to therapeutic reversal.[1,15–17] Recent breakthroughs demonstrate that global transcriptional networks can be rewired to restore tissue homeostasis without erasing cellular identity, highlighting the potential of non-genetic modulation in resolving fibrosis.[18–23] Building on this concept of phenotypic plasticity, we propose a pharmacological approach to reverse the fibrotic phenotype in pathological scar-forming fibroblasts, with the ultimate goal of achieving scar-free wound regeneration in keloids and hypertrophic scars.[4,24,25]

Here, leveraging DRUG-seq2[26]—an efficient 3′-end barcoding transcriptomic profiling method optimized from the Digital RNA with pertUrbation of Genes (DRUG-seq) technology—we evaluated the fibrosis-reversal potential of a library of putative anti-fibrotic small molecules. Our screen identified Rottlerin as a potent fibrosis-reversal agent that effectively attenuates collagen and α-SMA expression. Through structural optimization, we developed FR-1 (fibrosis-reversal compound 1), a derivative with enhanced solubility and druggability that retains robust fibrosis-reversal efficacy. Topical application of FR-1 significantly reduced scar area and preserved hair follicles in a murine wound model, outperforming the first-line therapy, TA. Crucially, these therapeutic effects were validated in patient-derived keloid *ex vivo* explants and an *in vivo* keloid xenograft model. Mechanistically, FR-1 induces mitochondrial uncoupling in profibrotic myofibroblasts to halt fibrosis progression. Ultimately, this work presents a promising small-molecule therapy targeting mitochondrial uncoupling to resolve fibrotic scars, demonstrating potent efficacy in skin regeneration and broad translational potential for multisystemic fibrosis.

RESULTS

DRUG-seq2 identifies chemical compounds for reversing skin fibrosis phenotypes in human dermal fibroblasts *in vitro*

To identify therapeutic agents with fibrosis-reversal properties, we employed a primary cell-based screening platform utilizing patient-derived dermal fibroblasts isolated from keloid lesions. Notably, TGF-β1 functioned as a profibrotic control, exerting its established profibrotic effects through the activation of small mothers against decapentaplegic (SMAD)2/3 .[27] Dimethyl sulfoxide (DMSO) served as the vehicle control. A library of 23 bioactive agents was selected based on anti-fibrotic annotations (Table S1), including representative inhibitors such as TGF-β type I receptor antagonists, VEGFR/FGFR inhibitors, and antioxidants reported to suppress myofibroblast activation.[28–30] The library further incorporated adipogenic reprogramming regulators to induce myofibroblast transdifferentiation into adipocytes,[24] targeting enhanced fat regeneration and reduced scar formation. DRUG-seq2 transcriptome profiling systematically categorized these screening agents into functional clusters with distinct features as follows: (1) profibrotic cluster: exemplified by TGF-β1, this group induced canonical fibrogenic signatures including *CTGF, MKI67*, and *CSRP2*; (2) fibrosis-reversal cluster: represented by sorafenib, a multi-kinase inhibitor showing potent anti-keloid efficacy through dual antagonism of TGF-β/SMAD and mitogen-activated protein kinase (MAPK)/ERK signaling pathways[31]; (3) adipogenic transdifferentiation cluster: headlined by MSC2530818 in adipocyte differentiation (AD) medium, this group activated the core regulatory factors for AD, such as *LEP*, *FABP4*, and *CIDEC* (Figures 1A, 1B, and S1A).

We next conducted gene set scoring to evaluate compound-mediated modulation of fibrosis-associated gene signatures. The gene sets comprised collagen fibril organization (GO: 0030199), ECM assembly (GO: 0085029), and fibroblast activation (GO: 0072537) from MSigDB, along with YAP/TAZ-upregulated genes obtained from the published data.[12] Only Rottlerin and sorafenib showed an obvious decrease in all four entries (Figures 1C and S1B). Sorafenib's clinical utility as a systemic antitumor agent is limited by its adverse effects, including hand-foot skin reaction (HFSR).[32] Topical delivery risks local drug accumulation and exacerbated cutaneous toxicity, while complicating precise dosing. Furthermore, sorafenib's poor transdermal permeability limits stratum corneum penetration via conventional vehicles, resulting in sub-therapeutic bioavailability. Therefore, we focused on Rottlerin, a natural compound isolated from the Asian tree *Mallotus phillippinensis*. Its precursor is traditionally used in Southeast Asian medicine for its antiparasitic and antibacterial properties.[33] Rottlerin exhibits a favorable safety profile in murine models[34] and suppresses pancreatic cancer growth by concurrently inhibiting Akt, Notch, and Shh signaling pathways—key drivers constitutively active in this malignancy.[35]

To further clarify the fibrosis-reversal properties of Rottlerin, we performed a dose-response study (0–3 μM) in patient-derived keloid fibroblasts. Bright-field imaging and quantitative cell counting confirmed a dose-dependent reduction in cell number, demonstrating potent inhibition of fibroblast proliferation.

CellPress
OPEN ACCESS

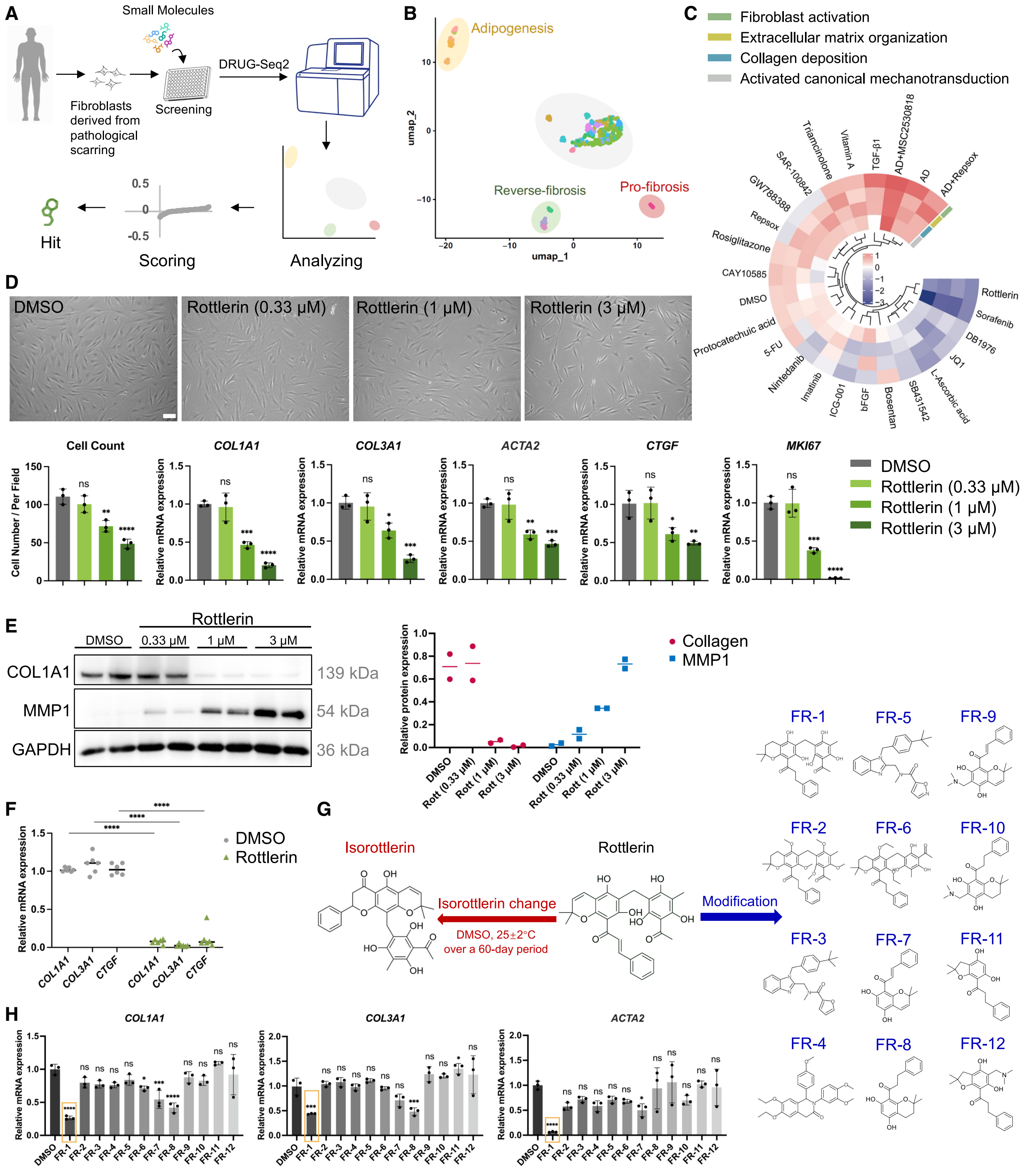

Figure 1. Transcriptome screening platform DRUG-seq2 identified chemical compounds for reversing skin fibrosis

(A) Schematic of the drug screening process: fibroblasts isolated from pathological scar tissue of patients underwent chemical library treatment, followed by DRUG-seq2 transcriptional profiling. Bioinformatics-driven prioritization yielded lead compounds, with phenotype-reversing hits undergoing further functional validation.

(B) Uniform manifold approximation and projection (UMAP) of DRUG-seq2 data from keloid fibroblasts treated with different compounds.

(C) Circular heatmap depicting ssGSEA enrichment scores for compound-mediated modulation of fibrosis-associated gene signatures.

(legend continued on next page)

Furthermore, quantitative reverse-transcription PCR (RT-qPCR) revealed a dose-dependent suppression of profibrotic myofibroblast markers: *collagen type I alpha 1 chain (COL1A1)* and *collagen type III alpha 1 chain* (*COL3A1*) mRNA levels were downregulated by >5-fold at 3 μM compared to controls (Figure 1D). Western blot analysis demonstrated that Rottlerin treatment drastically downregulated COL1A1 protein expression (decreasing by up to 98.24% at 3 μM) while concurrently upregulating matrix metalloproteinase-1 (MMP1) (increasing by up to 28-fold at 3 μM), a collagenase known for its proteolytic activity against various collagen subtypes (Figure 1E). These coordinated molecular alterations suggest that Rottlerin may effectively attenuate excessive collagen accumulation. To assess the broad therapeutic potential of Rottlerin across heterogeneous clinical populations, we evaluated its effects in scar-derived fibroblasts derived from 6 patients, encompassing variations in sex, age, scar location, and pathological subtype (keloid or hypertrophic scar) (detailed in Table S2, patient ID: 1–6). Following Rottlerin treatment, RT-qPCR analysis demonstrated a significant downregulation of fibrosis-associated markers, including *COL1A1*, *COL3A1*, and *CTGF*, compared with DMSO-treated controls (Figure 1F).

Modified compound FR-1 retains fibrosis-reversal efficacy with enhanced druggability

Previous studies demonstrate that Rottlerin undergoes time-dependent degradation in DMSO (25 ± 2°C, 60 days), with nuclear magnetic resonance (NMR) analysis identifying isorottlerin as the primary degradation product.[36] Furthermore, physicochemical characterization demonstrated that Rottlerin possesses limited aqueous solubility (≪0.01 mg/mL in phosphate-buffered saline [PBS], Table S3), severely restricting its bioavailability and formulation stability—critical parameters for pharmaceutical development. To address these limitations, we initiated a systematic structure-activity relationship (SAR) analysis and carried out targeted structural modifications to optimize solubility parameters while preserving its biological activity. Twelve Rottlerin derivatives (FR-1–FR-12) were synthesized and evaluated for their fibrosis-reversal potential (Figure 1G). RT-qPCR analysis demonstrated downregulation of *COL1A1*, *COL3A1*, and *ACTA2* mRNA levels in three derivatives (FR-1, FR-7, FR-8), with FR-1 exhibiting the strongest potency (Figure 1H).

Dose-response analysis (0–3 μM) of FR-1 revealed concentration-dependent suppression of fibrotic markers. The mRNA expression of *COL1A1* and *COL3A1* decreased by more than 5-fold at 3 μM (Figure 2A). Western blot analysis demonstrated that FR-1 treatment markedly downregulated COL1A1 protein expression (decreasing by up to 94.89% at 3 μM), coupled with upregulation of MMP1 (increasing by up to 4.5-fold at 3 μM), indicating that FR-1 could effectively attenuate excessive collagen accumulation (Figure 2B). To assess the broad therapeutic potential of FR-1 across heterogeneous clinical populations, we evaluated its effects in scar-derived fibroblasts from 6 patients, encompassing variations in gender, age, scar location, and pathological subtype (keloid or hypertrophic scar) (detailed in Table S2, patient ID: 5–10). Importantly, these therapeutic effects were recapitulated in a commercial keloid fibroblast line of African descent (Figures S2A and S2B). Following FR-1 treatment, RT-qPCR analysis demonstrated a significant downregulation of fibrosis-associated markers (*COL1A1*, *COL3A1*, *CTGF*) in all five scar-derived fibroblasts relative to the DMSO control (Figure 2C).

To compare the similarities and differences between Rottlerin and FR-1, we performed bulk RNA sequencing (RNA-seq). The results revealed a noticeable downregulation of fibrosis markers in the transcriptome compared with the DMSO control (Figure 2D). Gene ontology (GO) analysis indicated that both Rottlerin and FR-1 downregulated various collagen-related ECM components and actin filament organization (Figures 2E and 2F). Relative to Rottlerin, FR-1 demonstrated significant downregulation of cell division/mitosis-associated GO terms (Figure 2G), suggesting enhanced anti-proliferative potential. Gene set enrichment analysis (GSEA) of DisGeNET datasets revealed FR-1's stronger downregulation of keloid and dermatological disorders signatures compared with Rottlerin (Figures S2C and S2D). Furthermore, FR-1 exhibited superior suppression of pro-inflammatory pathways, such as IL-17 and TNF signaling cascades (Figure S2E; Table S4).

Next, we evaluated the druggability of FR-1 to demonstrate the translational potential of the modified derivative relative to its parent compound. Unlike Rottlerin, which contains an α,β--unsaturated ketone rendering it highly susceptible to spontaneous degradation via intramolecular Michael addition, FR-1 lacks this reactive moiety and exhibits robust chemical stability. With this enhanced stability established, comprehensive physicochemical and *in vitro* ADME (absorption, distribution, metabolism, and excretion) profiling was performed. The most significant improvement achieved by structural modification was a nearly 300-fold enhancement in aqueous solubility (1.79 μM for FR-1 vs. 0.006 μM for Rottlerin in PBS, Table S3). Additionally, FR-1 demonstrated acceptable *in vitro* metabolic stability across species and a moderate cytochrome P450 inhibition profile, which remains within a manageable range for topical applications (Table S3). Broad-spectrum kinase profiling (419 kinases) revealed that FR-1 possesses significantly weaker inhibitory potency than Rottlerin. When ranked by inhibition potency, FR-1 demonstrated <50% maximal inhibition across all targets (highest: 43.64% at 1 μM), whereas Rottlerin inhibited 14 of its top 20

(D) Bright-field microscopy of keloid fibroblasts treated with DMSO or Rottlerin (0.33, 1, and 3 μM; scale bars, 100 μm), with quantification of cell count. Relative mRNA expression of profibrotic markers (*COL1A1, COL3A1, ACTA2, CTGF*) and the cell proliferation marker *MKI67* was determined by RT-qPCR ($n = 3$).
(E) Western blot analysis of COL1A1 and MMP1 protein expression in keloid fibroblasts exposed to graded concentrations of Rottlerin (0.33, 1, and 3 μM) ($n = 2$).
(F) RT-qPCR analysis of *COL1A1, COL3A1*, and *CTGF* in keloid fibroblasts from six patients after treatment with Rottlerin at 3 μM ($n = 6$).
(G) SAR-guided structural optimization of Rottlerin yielding derivatives FR-1 to FR-12.
(H) Screening of fibrosis-reversal efficacy among twelve distinct modified compounds (FR-1 to FR-12) at a uniform concentration of 2 μM via RT-qPCR quantification of *COL1A1, COL3A1,* and *ACTA2* ($n = 3$). Data are mean ± SD; *n* represents the number of independent biological replicates. *$p < 0.05$; **$p < 0.01$; ***$p < 0.001$; ****$p < 0.0001$; ns, not statistically significant vs. DMSO by one-way ANOVA.

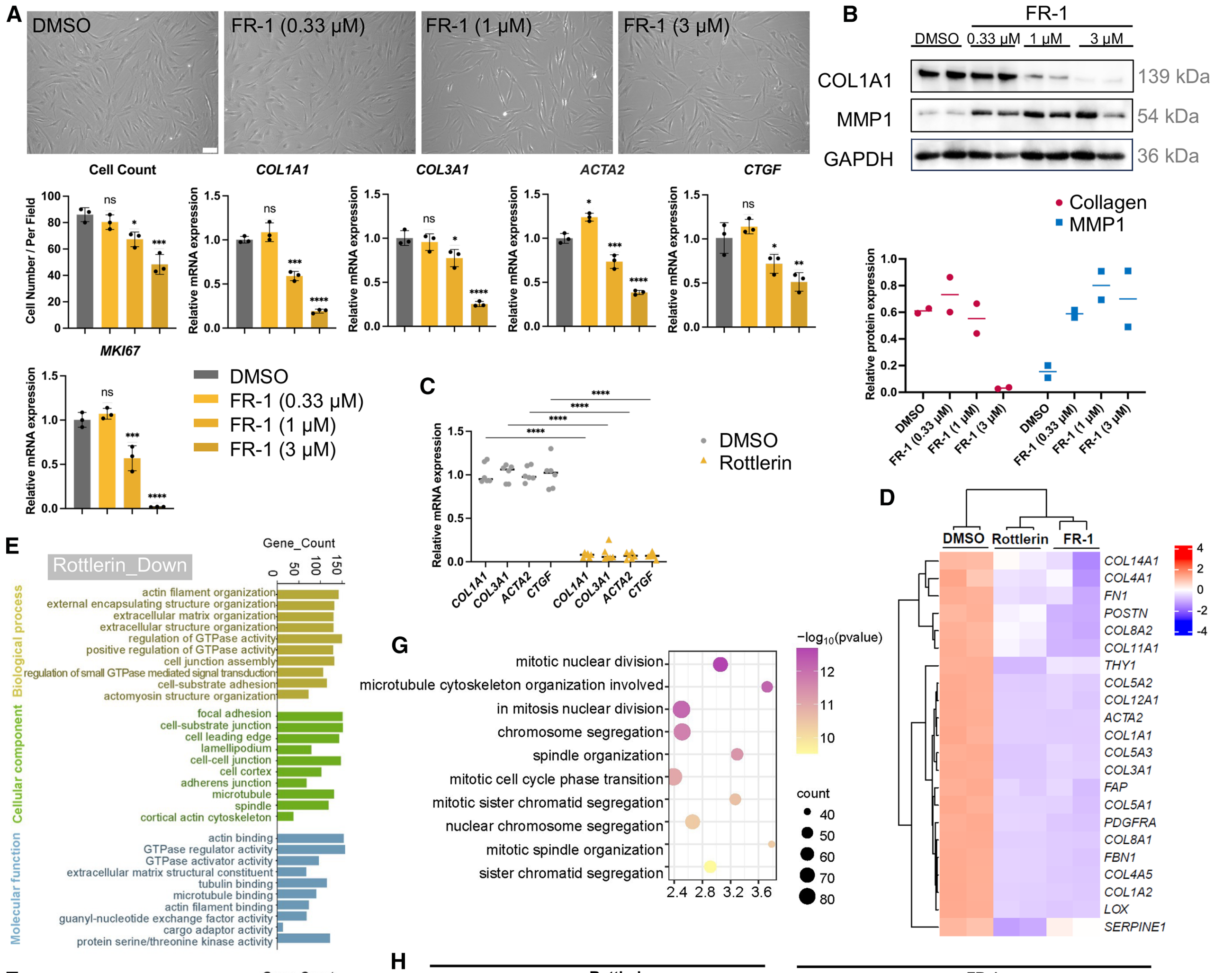

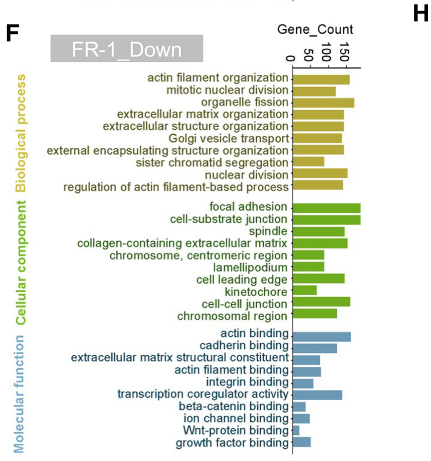

H

Kinase	Rottlerin (%inhibition) 1 μM
TTK	71.35321348
DDR1	62.82317425
DGKA	62.44162775
FLT3 ITD	61.74855171
RET V804M	57.43803217
FER	56.45356328
BRK	55.02599204
BLK	54.65759973
PDGFRα	54.04381987
FES	53.99771534
TRKA G595R	53.86904932
LYNb	51.91107737
TEC	51.38692207
KIT V559D	51.36991422
AXL	49.34697883
TYRO3	48.65367495
MUSK	48.11620674
TXK	47.27563724
RET V804E	46.79458406
PDGFRβ	46.21821985

Kinase	FR-1 (%inhibition) 1 μM
DDR1	43.64364281
BRK	42.89991980
RET V804E	37.17930781
CK2α1/β	36.21764889
LYNb	35.68915065
EGFR C797S L858R	34.41747667
FAK	34.38032951
TRKA G595R	34.36146166
FER	34.11967009
FLT3	33.36810396
BLK	32.74765531
MRCKα	32.59491761
PLK2	31.98624248
FLT3 ITD	31.92938369
TRKC G696A	31.22519164
WNK2	29.59434542
EPHB3	29.51737149
EPHA6	28.19767546
TYRO3	28.02903359
FLT1	27.71726824

Figure 2. Modified compound FR-1 retains fibrosis-reversal efficacy

(A) Bright-field microscopy of keloid fibroblasts treated with DMSO or FR-1 (0.33, 1, and 3 μM; scale bars, 100 μm), with quantification of cell count. Relative mRNA expression of profibrotic markers (*COL1A1, COL3A1, ACTA2, CTGF*) and the cell proliferation marker *MKI67* was determined by RT-qPCR ($n = 3$).

(B) Western blot analysis of COL1A1 and MMP1 protein expression in keloid fibroblasts exposed to graded concentrations of FR-1 (0.33, 1, and 3 μM) ($n = 2$).

(C) RT-qPCR analysis of *COL1A1, COL3A1, ACTA2*, and *CTGF* in keloid fibroblasts from six patients after treatment with FR-1 at 3 μM ($n = 6$).

(D) Clustered heatmap analysis of fibrosis-related genes based on bulk RNA-seq.

(legend continued on next page)

kinases by more than 50% under identical assay conditions (Figure 2H). This attenuated kinase engagement profile correlates with diminished off-target effects, indicating a reduced propensity for adverse pharmacological responses.

Intralesional FR-1 injection attenuates scar formation in a murine linear excisional wound model

Following our *in vitro* findings, we further investigated the therapeutic potential of FR-1 to attenuate scarring *in vivo*. Specifically, using a mouse *(Mus musculus)* linear excisional wound model, we administered FR-1 to treat established scars during the active remodeling phase. In this model, bilateral wound contraction generates lateral mechanical tension, producing visibly widened scars that maximize phenotypical resemblance to human skin scarring.[37] This experimental model, with FR-1 administration during the fibrotic phase, establishes a platform to assess the capacity for scar attenuation. In the preliminary experiments, FR-1 was administered via intralesional injection during the post-epithelialization (13 days post-injury, designated day 0, D0), with subsequent doses delivered every 4 days (D4, D8, D12) at 100 μL per side (Figure S3A). Both concentrations tested (0.1 and 0.01 mM) reduced scar size at D16 (Figures S3B–S3D). Scar areas measured by gross morphology were reduced to 0.076 ± 0.009 cm^2 ($p < 0.01$) and 0.065 ± 0.010 cm^2 ($p < 0.001$) in the 0.01 and 0.1 mM groups, respectively, versus vehicle (0.106 ± 0.007 cm^2) (Figure S3D). Histopathological assessment of hematoxylin and eosin (H&E)-stained sections obtained from the center of the linear scar confirmed decreased scar width (Figures S3E and S3F). Immunohistochemical analysis revealed marked reductions in α-SMA and TGF-β expression (Figures S3G–S3I). These findings were corroborated by RT-qPCR, showing downregulation of *Acta2* and *Tgfb1* transcripts, with *Acta2* exhibiting >2-fold reduction at 0.01 mM and *Tgfb1* exhibiting >17-fold reduction at 0.1 mM (Figure S3J).

Topical FR-1 exhibits macroscopic scar suppression comparable to the first-line medication TA

The efficacy of intralesional FR-1 injections prompted the development of a topical FR-1 ointment to further enhance clinical translatability. This non-invasive formulation leverages several inherent advantages, including ease of application, localized biodistribution, minimized systemic exposure, and improved patient adherence. To evaluate efficacy in this modality, we employed the murine linear excisional wound model, initiating daily ointment application during the active remodeling phase (13 days post-injury, designated D0; Figure 3A). Preliminary dose-response screening identified 0.2% FR-1 as a viable concentration for significant scar reduction without inducing observable skin irritation (Figure S4). Using this regimen, we assessed topical efficacy in the murine linear excisional wound model with daily application across four groups: vehicle, blank control, topical FR-1 (0.2%), and TA (a first-line clinical medication for keloids and hypertrophic scars) ointment groups. In the curve chart depicting changes in scar area, it can be observed that scars in mice from the vehicle and blank groups displayed a propensity to expand due to contraction of the surrounding skin. In contrast, scars in mice from the FR-1 and TA groups exhibited a trend of reduction (Figures 3B and 3C). By D12, gross observation and scar area quantification revealed significant scar reduction with FR-1 (0.056 ± 0.005 cm^2; $p < 0.05$ vs. vehicle), achieving efficacy comparable to TA with no statistical difference between treatments (Figure 3D). Notably, this significant therapeutic efficacy appeared earlier than in the injection model (which reached significance at D16, Figures S3B–S3D). Consistent with these macroscopic findings, histopathological analysis of H&E-stained central scar sections at D12 confirmed significant scar width reduction in both FR-1 and TA groups versus vehicle controls (Figures 3E and 3F).

FR-1 drives near-native dermal remodeling while avoiding TA-associated adverse effects

Despite achieving comparable scar reduction, TA administration induced hallmark adverse effects, including epidermal thinning (46% reduction vs. normal skin, $p < 0.05$), decreased cellularity (27% reduction vs. normal skin, $p < 0.05$), loss of subcutaneous architecture, and collagen disorganization (Figures 3G–3I), consistent with reported limitations of this first-line clinical therapy.[5,8,9,38] Conversely, FR-1 preserved near-native epidermal thickness and physiological cellularity (both showing no significant difference vs. normal skin). Furthermore, while no group fully restored hair follicle density to normal skin levels (11–12 follicles per high-power field [HPF]), the FR-1 group exhibited a significantly higher number of hair follicles compared to the other groups, whereas the TA group showed negligible follicles (Figure 3J). Additionally, immunofluorescence α-SMA/β-catenin co-staining demonstrated attenuated hair follicle destruction in FR-1-treated scars versus vehicle and TA groups (Figure 3K). This co-staining strategy builds on established roles of α-SMA in scarring and β-catenin in hair follicle development, allowing integrated analysis of fibrosis and remodeling.[39] FR-1 induced co-localization (Pearson's R = 0.678 ± 0.057; overlap R = 0.764 ± 0.046) that markedly exceeded vehicle and TA effects and was comparable to normal skin (Figure 3L), with both FR-1 and normal skin groups exceeding established thresholds (Pearson's R > 0.5, overlap R > 0.6).[40]

FR-1 minimized scarring while avoiding TA-associated adverse effects, achieving histological parameters approaching near-native skin. Under polarized light, Sirius red staining demonstrated significantly reduced collagen deposition in the FR-1 group, with preferential reduction in thicker collagen I fibers (Figure 3M). This correlated with a significantly lower collagen I/III ratio versus TA and vehicle groups ($p < 0.01$, Figure 3N)—a critical shift given that excessive collagen I drives fibrosis, while a

(E and F) Top 10 significantly downregulated GO terms in (E) Rottlerin- and (F) FR-1-treated groups relative to DMSO controls.
(G) Enriched biological processes comparing FR-1 versus Rottlerin treatment.
(H) Kinase inhibition profile of Rottlerin and FR-1. Data are mean ± SD, *n* represents the number of independent biological replicates. *$p < 0.05$; **$p < 0.01$; ***$p < 0.001$; ****$p < 0.0001$; ns, not statistically significant vs. DMSO by one-way ANOVA.

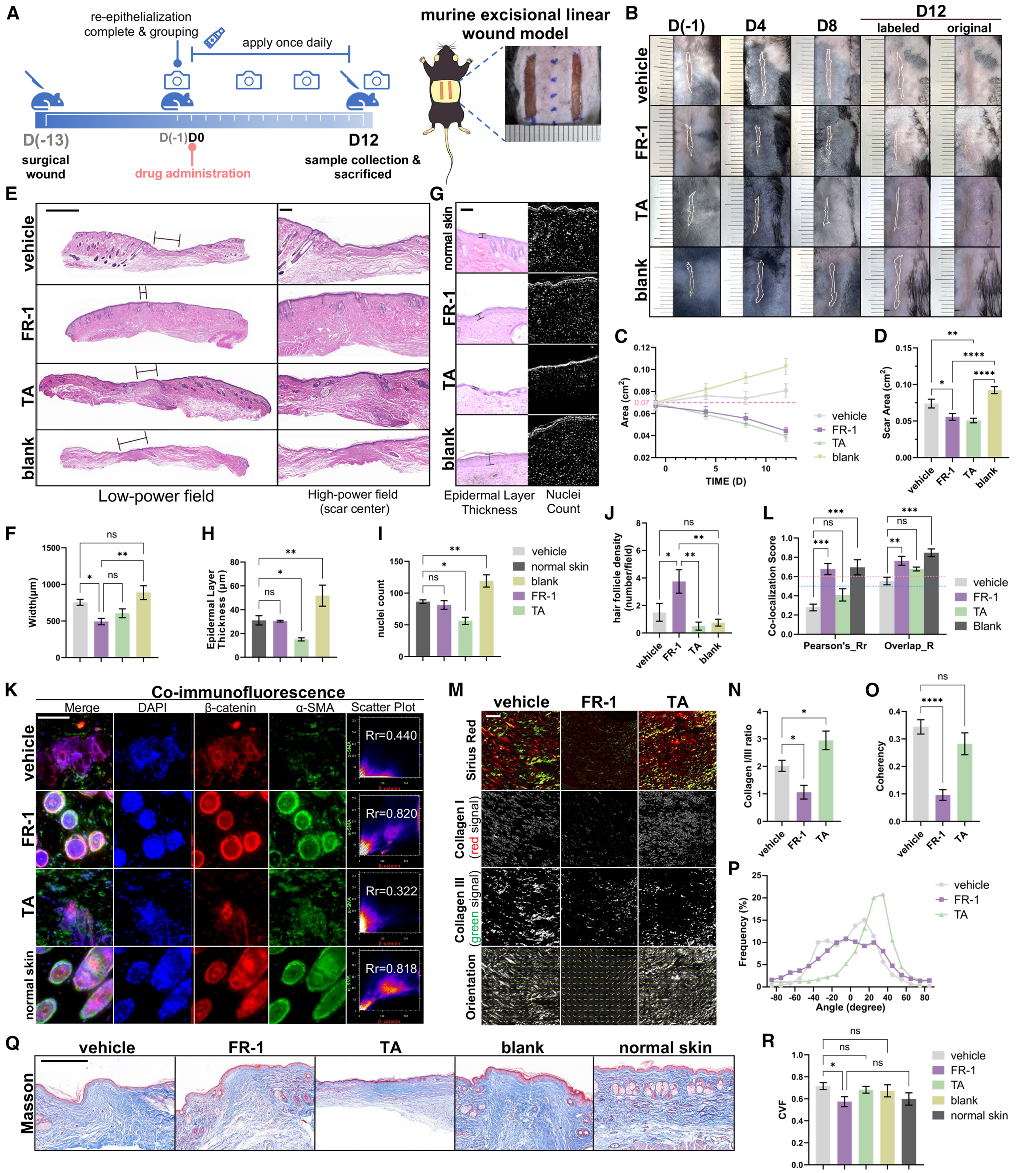

Figure 3. FR-1 attenuates established scars and preserves hair follicles in a murine linear excisional wound model

(A) Schematic and timeline of the murine linear excisional wound model (1.5 × 0.2 cm wounds, 0.4 cm lateral to the midline).

(B) Representative images of scar progression in vehicle-, FR-1-, TA-, and blank (surgery only)-treated groups at indicated time points (white boxes: scar areas; *n* = 10 scars from 5 mice per group). Quantification of (C) scar dynamic changes (line plots) and (D) scar area at day 12 (bar graph).

(E) H&E-stained scar sections (scale bars, 1000 μm in low-power field, 200 μm in high-power field), (F) scar width quantification (*n* = 5 mice).

(legend continued on next page)

higher collagen III proportion (characteristic of fetal scarless healing) favors regenerative remodeling.[41,42] Quantitative topological analysis further revealed that FR-1 treatment significantly decreased the coherency index (Figure 3O), indicating a restoration of physiological multidirectional fiber alignment. This structural normalization was further corroborated by the fiber angle distribution curve, which shifted from a sharp, narrow peak typical of parallel scarring (vehicle) to a broad, flattened profile (FR-1) (Figure 3P).[43,44] Masson's trichrome staining demonstrated reduced collagen deposition in the FR-1 group ($p < 0.01$ vs. vehicle), with levels closely resembling those in normal skin (not statistically significant), compared with other groups, as confirmed by collagen volume fraction (CVF) analysis (Figures 3Q and 3R). Unlike TA, which induced pathological skin alterations, FR-1 treatment promoted near-native wound regeneration, representing a significant step toward scarless healing. In conclusion, FR-1 reduces excessive collagen accumulation while maintaining physiological architectural stability, ensuring that fibrosis attenuation does not compromise the biomechanical integrity of the healed skin.

Given the significantly higher permeability of murine skin compared to human skin,[45] we further assessed human-relevant biodistribution via *in vitro* permeation testing (IVPT) on full-thickness porcine (*Sus scrofa domesticus*) skin. Even under maximal exposure conditions (1% ointment), FR-1 formed a localized depot in the dermis (2.88 ± 0.32 μg/g) while remaining undetectable in the receptor fluid (Table S5). Consistent with this minimal systemic absorption, pathological examination of major organs (heart, liver, spleen, lung, and kidney) in FR-1-treated mice at the experimental endpoint following continuous daily administration revealed no adverse pathological changes (Figure S5).

FR-1 prevents fibrosis progression and preserves hair follicles during acute wound healing *in vivo*

To assess prophylactic efficacy in nascent fibrosis, we employed a murine splinted excisional wound model with a single local injection of 0.1 mM FR-1 (100 μL/side) at the wound base immediately post-surgery (acute-phase intervention, designated D0; Figures 4A and 4B), without subsequent treatment. In this model, silicone rings prevent wound contraction to mimic human-like healing via re-epithelialization and granulation.[46] Longitudinal photographic analysis revealed that the FR-1 group significantly reduced scar areas at day 60 (0.011 ± 0.005 vs. 0.023 ± 0.011 cm^2, $p < 0.01$) compared with the vehicle group, as quantified by ImageJ-based morphometric assessment (Figures 4C and 4D). Notably, FR-1 did not delay wound closure (Figure S6). Although the irregular boundaries of late-stage scars in the splinted model limit the reliability of absolute width quantification, histopathological evaluation (H&E, D60) qualitatively demonstrated a consistent trend wherein FR-1-treated scars were visibly narrower than those in the vehicle group (Figure 4E). Additionally, Masson's trichrome staining with quantitative image analysis demonstrated significantly reduced CVF ($p < 0.05$) in the FR-1-treated group (Figures 4F and 4H). At day 60, FR-1-treated wounds exhibited significantly more hair follicles than vehicle controls (Figures 4E and 4G), also evidenced by α-SMA/β-catenin co-localization (Pearson's R = 0.553 ± 0.045; overlap R = 0.794 ± 0.033, Figures 4I and 4J). Crucially, combining this acute-phase intervention regimen with our remodeling-phase intervention regimen demonstrates FR-1's consistent efficacy across divergent stages of fibrotic attenuation.

FR-1 mitigates local fibroblast activity in patient-derived keloid *ex vivo* explant and xenograft models

To complement these *in vivo* murine findings and address potential species-specific differences, we utilized an *ex vivo* keloid explant model with TA as a clinical positive control (Figure 5A). This platform preserves the native 3D architecture, ECM, and complex signaling of diseased tissue, overcoming 2D monoculture limitations. Over 7 days, vehicle-treated (DMSO) explants exhibited robust, continuous fibroblast outgrowth. While 1 μM TA attenuated this expansion, the cell population maintained a steady growth trajectory. In stark contrast, 1 μM FR-1 exhibited profound inhibition: after minimal initial migration by day 2, it completely arrested further outgrowth, maintaining flat cell counts through day 7 (Figure 5B). These quantitative findings demonstrate that FR-1 potently disrupts keloid cell migration and proliferation, significantly outperforming the clinical standard TA (Figure 5C).

To further evaluate the translational potential of FR-1 in a human-relevant context, we established a human keloid patient-derived xenograft (PDX) model in nude mice (Figures 5D and 5E). Macroscopic evaluation (Figure 5F) revealed that although FR-1-treated grafts exhibited a smaller mean volume, this downward trend did not reach statistical significance ($p > 0.05$) due to PDX heterogeneity (Figure 5G). Scatterplots revealed different patterns: vehicle grafts exhibited high variability, while the FR-1 group clustered tightly around a lower mean volume. Furthermore, overlapping body weight trajectories confirmed that repeated FR-1 injections were well tolerated without overt toxicity (Figure 5H). Histologically, both H&E and Masson's

(G) Representative images of epidermal architecture in treated vs. normal skin (H&E; scale bars, 100 μm). Quantification of (H) epidermal thickness and (I) nuclear density ($n = 5$ mice).
(J) Hair follicle density ($n = 4$ randomly selected representative high-power fields [HPFs] from 3 to 4 mice).
(K) α-SMA/β-catenin co-staining (scale bars, 100 μm). Split channels (β-catenin, red; α-SMA, green) highlight follicular structures, and (L) correlation coefficients ($n = 4$ HPFs from 2 to 3 mice).
(M) Representative polarized light images of Sirius Red staining; separated type I (red) and type III (green) collagen signals are shown, along with corresponding vector field maps visualizing fiber orientation (scale bars, 50 μm). Quantification includes (N) type I/III collagen ratio ($n = 3$ mice), (O) coherency index ($n = 6$ HPFs), and (P) fiber angle distribution.
(Q) Masson's trichrome staining (scale bars, 500 μm) and (R) CVF analysis ($n = 5$ mice). Note: "Blank" represents the baseline for untreated pathological scarring, "Vehicle" indicates the ointment base control, serving as the strict negative control to isolate pharmacological effects from baseline healing variation. Data are mean ± SEM; n represents the number of independent biological replicates. $^{*}p < 0.05$; $^{**}p < 0.01$; $^{***}p < 0.001$; $^{****}p < 0.0001$; ns, not statistically significant by one-way ANOVA.

CellPress
OPEN ACCESS

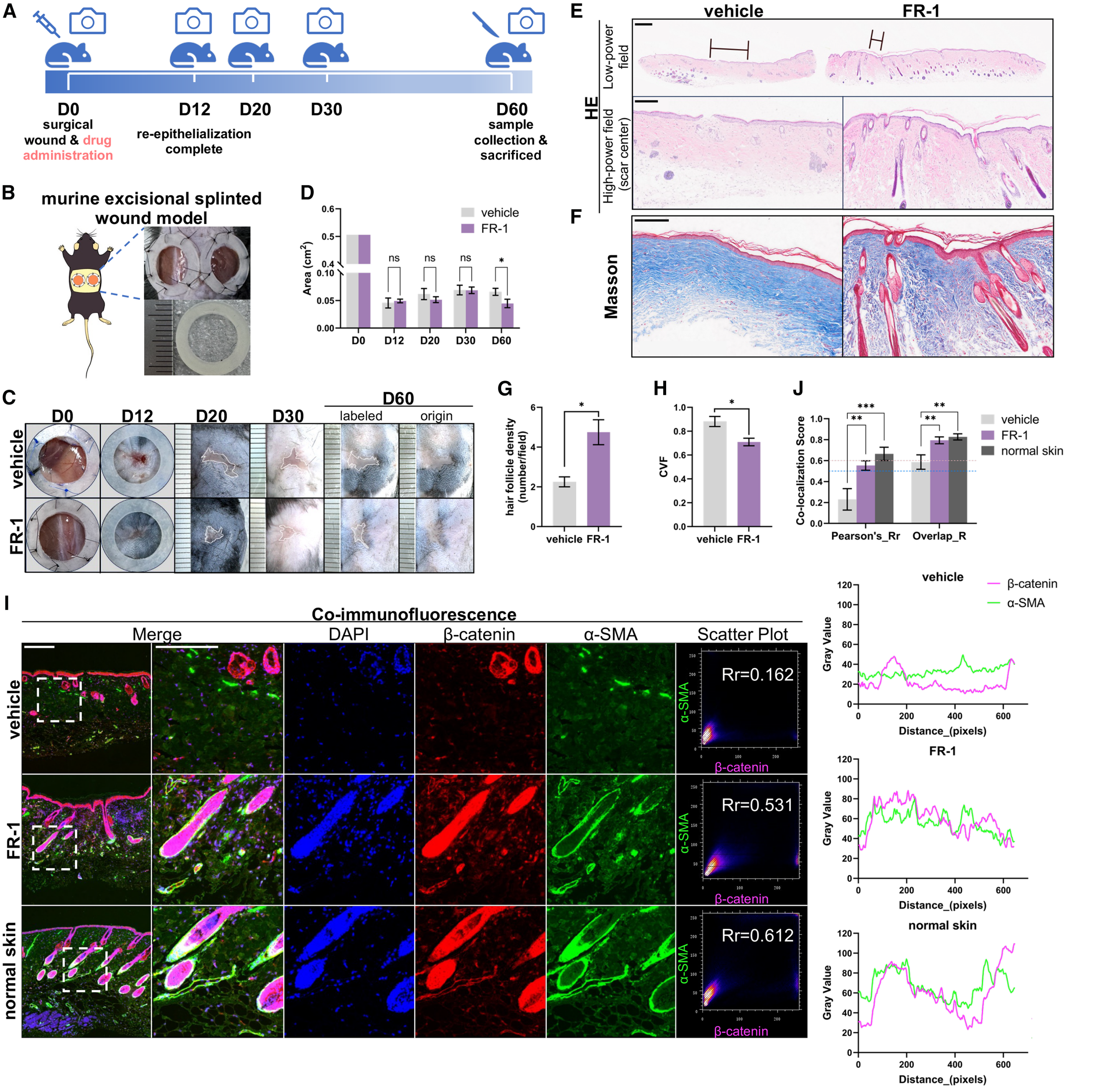

Figure 4. FR-1 attenuates fibrotic scarring and preserves hair follicles in a murine splinted excisional wound model

(A and B) Schematic and timeline (A) of the murine splinted excisional wound model (silicone ring: 8/15 mm inner/outer diameter) (B).

(C) Representative images of wound healing and scar formation progression (D0–D60) with vehicle or FR-1 treatment.

(D) Quantification of wound/scar areas over time (n = 6–10 scars from 3 to 5 mice per group). Representative (E) H&E (black lines mark scar width) and (F) Masson's trichrome staining (scale bars, 500 μm; high-magnification H&E: 200 μm).

(G) Quantification of hair follicle density (n = 4 HPFs from 2 to 3 mice) and (H) CVF (n = 3 mice).

(I) β-catenin/α-SMA colocalization in scar tissue (day 60). Left: Representative IF images with split channels (β-catenin, red; α-SMA, green) (scale bars, 200 μm). Right: Scatterplots (diagonal distribution) and fluorescence intensity curves showing colocalization.

(J) Pearson's Rr and Overlap R coefficients (n = 4 HPFs from 2 mice; Rr = 0.5–1.0, overlap R = 0.6–1.0 indicate colocalization). Data are mean ± SEM; n represents the number of independent biological replicates. $^{*}p < 0.05$; $^{**}p < 0.01$; $^{***}p < 0.001$ vs. vehicle by Student's t test (D, G, and H) or by one-way ANOVA (J).

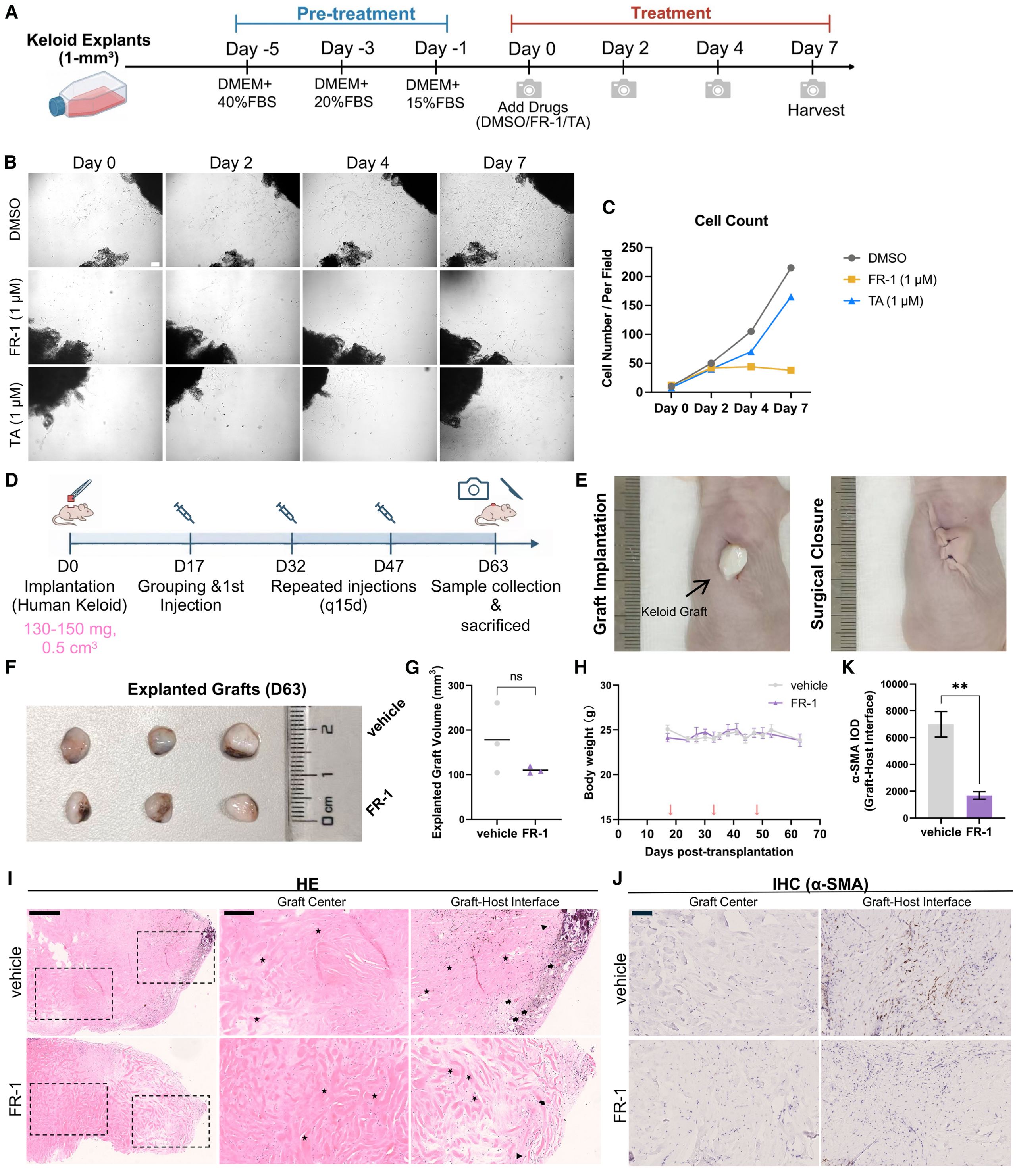

Figure 5. FR-1 suppresses keloid progression in patient-derived *ex vivo* and xenograft models

(A) Keloid tissue explants were pre-cultured and subsequently treated with vehicle (DMSO), 1 μM FR-1, or 1 μM TA (designated as treatment day 0).

(B) Representative micrographs illustrating cell outgrowth from the explants, captured at identical fields of view on treatment days 0, 2, 4, and 7 (Scale bars, 100 μm).

(C) The number of migrated cells was quantified at treatment days 0, 2, 4, and 7 (*n* = 3).

(D) Schematic timeline of the experimental design.

(legend continued on next page)

trichrome staining revealed that the central regions of viable grafts maintained typical keloid architecture, with no discernible difference in dense collagen deposition between the two groups (Figures 5I and S7A). However, at the graft-host interface, FR-1-treated grafts exhibited markedly reduced vascular congestion and inflammatory infiltration compared with vehicle controls (Figure 5I). Consistently, while central regions showed minimal α-SMA, at these active growth margins, FR-1 significantly reduced its expression by approximately 4-fold compared with the vehicle group ($p < 0.01$, Figures 5J and 5K).

Finally, histopathological evaluation of major organs at the experimental endpoint revealed no observable pathological abnormalities, further confirming the systemic safety of FR-1 *in vivo* (Figure S7B).

FR-1-driven fibrosis-reversal activity is independent of typical kinase inhibition, apoptosis, and cellular senescence

Previous reports indicated that the lead compound Rottlerin primarily targets protein kinase C (PKC) family members (Table S6).[47–50] Although this polyphenolic compound has been extensively characterized for its broad pharmacological properties, including anticancer, anti-inflammatory, antioxidant, immunosuppressive, antimicrobial, and antiviral activities,[48,51,52] its potential effects on fibrosis remain incompletely understood. To elucidate whether the antifibrotic activity of Rottlerin and FR-1 operates through PKC inhibition, we employed two structurally distinct PKC inhibitors (GO6983 and Sotrastaurin) with established potency. Notably, these PKC inhibitors failed to elicit statistically significant reductions in mRNA expression levels of key fibrotic markers (*ACTA2*, *COL1A1*, and *COL3A1*) (Figure 6A). These findings conclusively demonstrate that the fibrosis-reversal mechanisms of both Rottlerin and its derivative FR-1 are mechanistically independent of PKC pathway modulation, consistent with reports indicating that Rottlerin has little PKC inhibitory effects.[47]

Comprehensive kinase profiling (419 kinases spanning all major families) revealed FR-1's distinct profile, exhibiting <50% maximal inhibition (peak 43.64% at 1 μM)—below the ≥50% activity threshold defining conventional kinase inhibitors in primary screens.[53–55] This fundamental divergence suggests that kinase inhibition may not dominate its mechanism of fibrosis reversal (Figure 2H).

Published data suggest that Rottlerin upregulates pro-apoptotic executors (BAX, BID) and P21, a central regulator of cellular senescence.[56] This raises a critical question: whether its fibrosis-reversal effect is predominantly mediated by apoptosis or senescence. Flow cytometric analysis (Annexin V/PI) revealed no significant apoptosis in cells treated with FR-1 (3 μM) for 48 h compared with DMSO controls (Figure S8A). Although prolonged exposure (3 μM, 120 h) increased the proportion of apoptotic cells, an optimized lower-dose regimen (1 μM, 120 h) achieved comparable fibrosis-reversal efficacy without inducing significant apoptosis (Figures S8B and S8C). This was corroborated *in vivo*, as TUNEL staining of murine linear excision scars showed no significant apoptosis following FR-1 treatment (Figure S8D). Furthermore, to investigate cellular senescence, we performed SA-β-gal staining. Given that our standard *in vitro* assays utilize early-passage keloid fibroblasts, both the DMSO and FR-1 groups expectedly exhibited negligible staining, whereas late-passage positive controls displayed robust positivity, confirming that FR-1 does not induce premature senescence (Figure S8E). Consistently, RT-qPCR confirmed no concurrent upregulation of senescence markers (*P16*, *P21*) (Figure S8F). Collectively, these results demonstrate that the fibrosis-reversal efficacy of FR-1 is mechanistically independent of both apoptotic and cellular senescence pathways.

FR-1 is a highly potent mitochondrial uncoupler

Building on the established uncoupling capacity of the parent compound Rottlerin,[47] we hypothesized that FR-1 modulates mitochondrial bioenergetics to exert its fibrosis-reversal effects. Initial ratiometric JC-1 assays in 3T3-L1 fibroblasts revealed a reduction in mitochondrial membrane potential ($\Delta\Psi_m$) upon FR-1 treatment, evidenced by a significant decrease in the red/green fluorescence ratio (Figure S9A). Similarly, this decrease in $\Delta\Psi_m$ was also observed in patient-derived keloid fibroblasts, where tetramethylrhodamine ethyl ester (TMRE) staining demonstrated a consistent reduction in fluorescence intensity (Figure 6B).

This bioenergetic stress precipitated significant structural remodeling: mitochondria transitioned from a tubular network to a fragmented, punctate morphology upon FR-1 treatment (Figure 6C). Concurrently, a dose-dependent reduction in the ATP/ADP ratio was observed (Figure 6D), confirming that dissipation of the proton gradient directly impaired ATP synthesis efficacy.

To definitively characterize this uncoupling activity, we performed real-time respirometry (Seahorse XF analysis) on pre-treated cells (Figures 6E and S9B). FR-1 induced a distinct metabolic phenotype characterized by significantly elevated basal respiration. Crucially, this enhanced oxygen consumption was insensitive to the ATP synthase inhibitor oligomycin. This lack of respiratory control signifies that electron transport is no longer coupled to ATP synthesis but is instead driven by proton leak, a hallmark of mitochondrial uncoupling.

(E) Representative images of the surgical implantation and wound closure.
(F) Macroscopic appearance of explanted grafts at day 63.
(G) Scatterplot analysis of explanted graft volume from vehicle- and FR-1 (0.1 μM)-treated groups, measured by digital calipers and calculated as $V = 0.5 \times \text{length} \times \text{width}^2$ ($n = 3$ mice).
(H) Body weight changes of mice during the treatment period. Red arrows indicate injection time points ($n = 3$ mice).
(I) Representative H&E staining of explanted grafts at day 63. Stars indicate hyalinized collagen bundles; arrows indicate microvessels; arrowheads indicate representative inflammatory cells. Scale bars, 500 μm (left) and 200 μm (middle and right).
(J) Representative immunohistochemical staining (scale bars, 100 μm) and (K) quantification of α-SMA at the graft-host interface ($n = 3$ mice). Note: All xenografts were derived from the keloid tissue of a single patient donor. Data represent mean ± SEM; n represents the number of independent biological replicates, except for (C), where n represents technical replicates. $^{**}p < 0.01$; ns, not significant vs. vehicle by Student's t test.

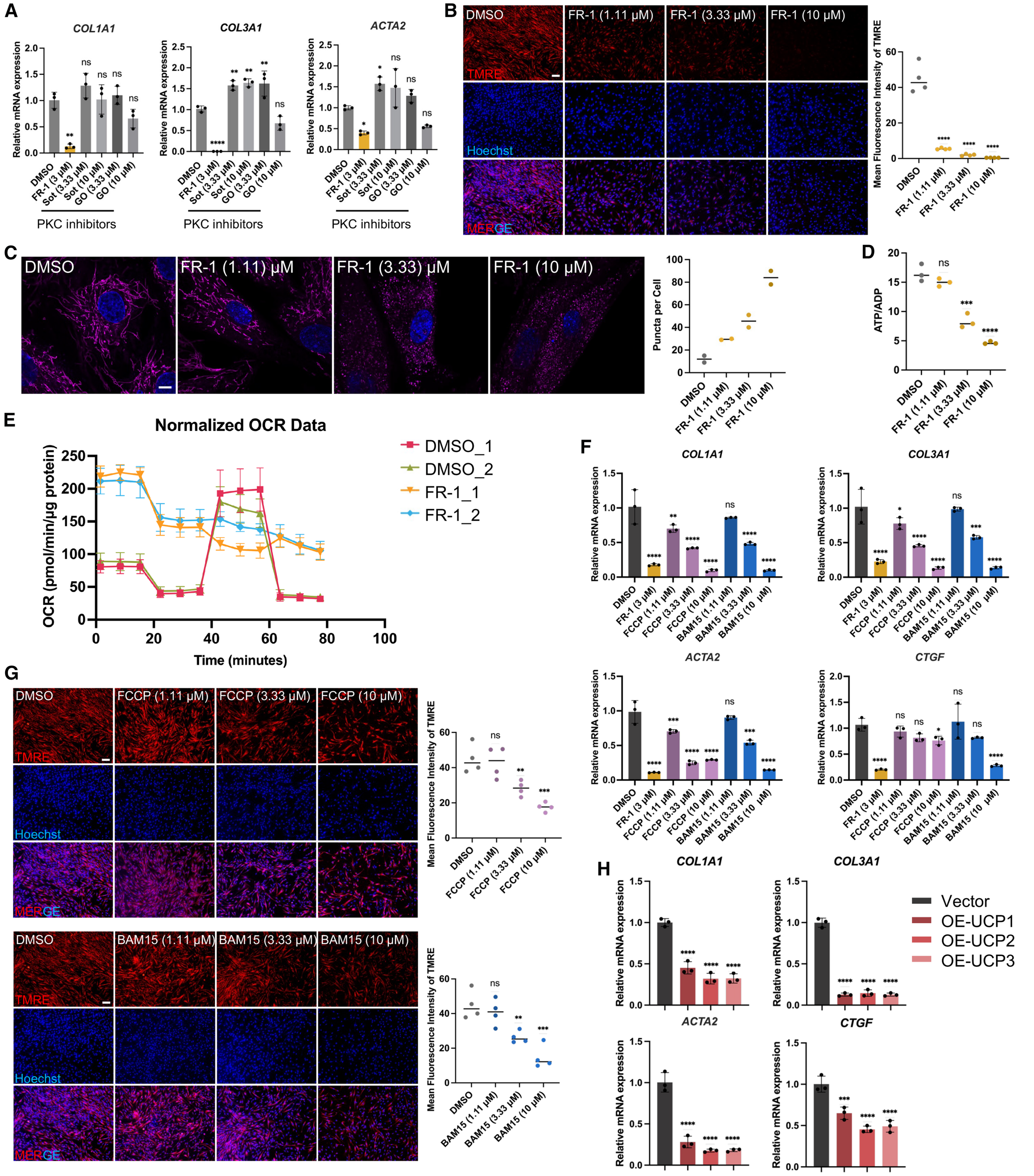

Figure 6. The mode of action for FR-1's fibrosis-reversal effects is mitochondrial uncoupling

(A) RT-qPCR analysis of *COL1A1, COL3A1*, and *ACTA2* in human keloid fibroblasts after treatment with different PKC family inhibitors ($n = 3$).

(B) Mitochondrial membrane potential detection (TMRE) in keloid fibroblasts treated with increasing concentrations of FR-1 (scale bars, 200 μm, $n = 4$).

(C) Mitochondrial uncoupling morphology detection and puncta quantification in human keloid fibroblasts treated with increasing concentrations of FR-1 (scale bars, 10 μm, $n = 2$).

(legend continued on next page)

The mode of action for FR-1's fibrosis-reversal effects is mitochondrial uncoupling

To investigate whether FR-1 reverses fibrosis through mitochondrial uncoupling, we comparatively evaluated two classical uncouplers: carbonyl cyanide 4-(trifluoromethoxy)phenylhydrazone (FCCP) and (2-fluorophenyl){6-[(2-fluorophenyl)amino]-1,2,5-oxadiazolo[3,4-b]pyrazin-5-yl}amine (BAM15).[57,58] RT-qPCR analysis demonstrated dose-dependent downregulation of fibrotic markers (*ACTA2, CTGF, COL1A1, COL3A1*) in FCCP- and BAM15-treated groups (Figure 6F), although their efficacy remained inferior to FR-1 at equimolar doses. To examine whether this disparity correlates with differential uncoupling capacity, we systematically quantified mitochondrial membrane potential ($\Delta\Psi_m$) across concentration gradients. TMRE-based $\Delta\Psi_m$ quantification (Figure 6G) demonstrated reduced uncoupling efficiency in FCCP and BAM15 relative to FR-1, mechanistically accounting for their diminished fibrosis-reversal efficacy.

Building on evidence that FR-1's fibrosis-reversal effects originate from mitochondrial uncoupling (as demonstrated by small-molecule inhibitors), we investigated whether ectopic expression of mitochondrial uncoupling proteins (UCP1/2/3) could recapitulate these therapeutic outcomes. Using a doxycycline-inducible Tet-On system, we confirmed exogenous UCP expression through RNA quantification and mitochondrial membrane potential detection (Figures S10A–S10C). Subsequent investigations demonstrated marked attenuation of fibrotic markers, as evidenced by significant downregulation of *COL1A1, COL3A1*, and *ACTA2* transcripts at the mRNA level (Figure 6H). These results demonstrate that mitochondrial uncoupling, whether chemically induced or achieved through UCP overexpression, effectively attenuates fibrosis progression.

In vivo, to further validate the role of mitochondrial uncoupling, we assessed the fibrosis-attenuating effects of the uncoupler FCCP in the murine linear excisional wound model and murine splinted excisional wound model (following the same protocol as FR-1 in the related model, Figures 7A and 7H). In the linear excisional wound model, intralesional injection of FCCP (1 mM) revealed a reduction in scar area in the FCCP group relative to the vehicle group (0.062 ± 0.001 vs. 0.084 ± 0.004 cm^2; $p < 0.01$, Figures 7B and 7C), along with markedly decreased scar width and collagen deposition (Figures 7D–7G). Consistently, in the splinted excisional wound model, FCCP (1 mM) treatment also resulted in a significant reduction in scar size compared with the vehicle group (0.047 ± 0.004 vs. 0.066 ± 0.006 cm^2; $p < 0.05$, Figures 7I and 7J) and a significant decrease in CVF (Figures 7L and 7M). Although irregular wound contraction in this model limits reliable width quantification, FR-1- and FCCP-treated scars were visibly narrower (Figure 7K). Notably, FCCP requires a 10-fold higher concentration (1 mM) than FR-1 (0.1 mM) to achieve comparable outcomes.

DISCUSSION

Through transcriptome-guided screening and lead optimization, we developed FR-1, a mitochondrial uncoupler that effectively reverses fibrosis via both subcutaneous and topical delivery. In contrast to the first-line therapy TA, which induces pathological skin damage, FR-1 attenuates scarring while preserving skin architecture and appendages. This work demonstrates mitochondrial uncoupling as a mechanistically distinct strategy for fibrosis, presenting FR-1 as a translational therapeutic candidate for near-native skin remodeling and broader multi-organ fibrotic diseases.

FR-1 exhibits significant translational potential by overcoming the critical limitations of current antifibrotic therapies. From a mechanistic perspective, an emerging compound such as verteporfin is largely restricted to prophylactic use because it strictly targets early tension-driven mechanotransduction.[4] Our results demonstrate that FR-1 enables effective intervention during both the acute wound stage and early scar formation (post-re-epithelialization) through mitochondrial uncoupling. Beyond this mechanistic distinction, FR-1 circumvents the practical barriers associated with delivery and safety. Specifically, invasive procedures and adjuvant radiotherapy carry risks of collateral damage and mutagenesis,[6,59] and standard pharmacotherapies compromise skin integrity.[5,8,9] Furthermore, many emerging compounds often face challenges such as narrow therapeutic windows or poor tissue penetration.[20] Agents like dimethyl fumarate (DMF) mitigates systemic sclerosis; however, its evaluation relies exclusively on intraperitoneal injection, leaving its topical viability for cutaneous scarring unexplored.[13] FR-1's optimized topical delivery ensures localized efficacy with minimal off-target toxicity. Preliminary evidence in a murine model of myocardial infarction demonstrates that FR-1 also effectively attenuates myocardial fibrosis (Figure S11). These findings highlight FR-1's broad therapeutic utility and strong clinical translatability for multisystemic fibrotic diseases.

The safety profile of topical FR-1 is substantiated by converging pharmacological evidence. Specifically, IVPT analysis demonstrates that FR-1 forms a localized dermal depot with negligible systemic absorption, indicating a substantial safety margin.[60,61] This pharmacokinetic profile aligns with recent consensus advocating reduced long-term toxicity testing requirements for dermal therapeutics with minimal systemic exposure.[62,63] Our toxicity assessment at the endpoint of *in vivo* study showed no adverse histopathological findings in major organs at therapeutic doses, and no signs of cutaneous irritation were observed over a moderate treatment duration. Furthermore, the lead compound

(D) ATP/ADP mass spectrometry detection following treatment of keloid fibroblasts with increasing concentrations of FR-1 ($n = 3$).
(E) Seahorse XF analysis of real-time oxygen consumption rate (OCR) profiles in fibroblasts treated with FR-1 or DMSO ($n = 4$).
(F) RT-qPCR analysis of fibrosis biomarkers in human keloid fibroblasts after treatment with different mitochondrial uncoupling compounds, FCCP and BAM15 ($n = 3$).
(G) Mitochondrial membrane potential detection in keloid fibroblasts treated with different mitochondrial uncoupling compounds (scale bars, 200 μm, $n = 4$).
(H) RT-qPCR analysis of fibrosis biomarkers in human keloid fibroblasts after overexpression of mitochondrial uncoupling proteins ($n = 3$). Data are mean ± SD; n represents the number of independent biological replicates. *$p < 0.05$; **$p < 0.01$; ***$p < 0.001$; ****$p < 0.0001$; ns, not statistically significant vs. DMSO or vector by one-way ANOVA.

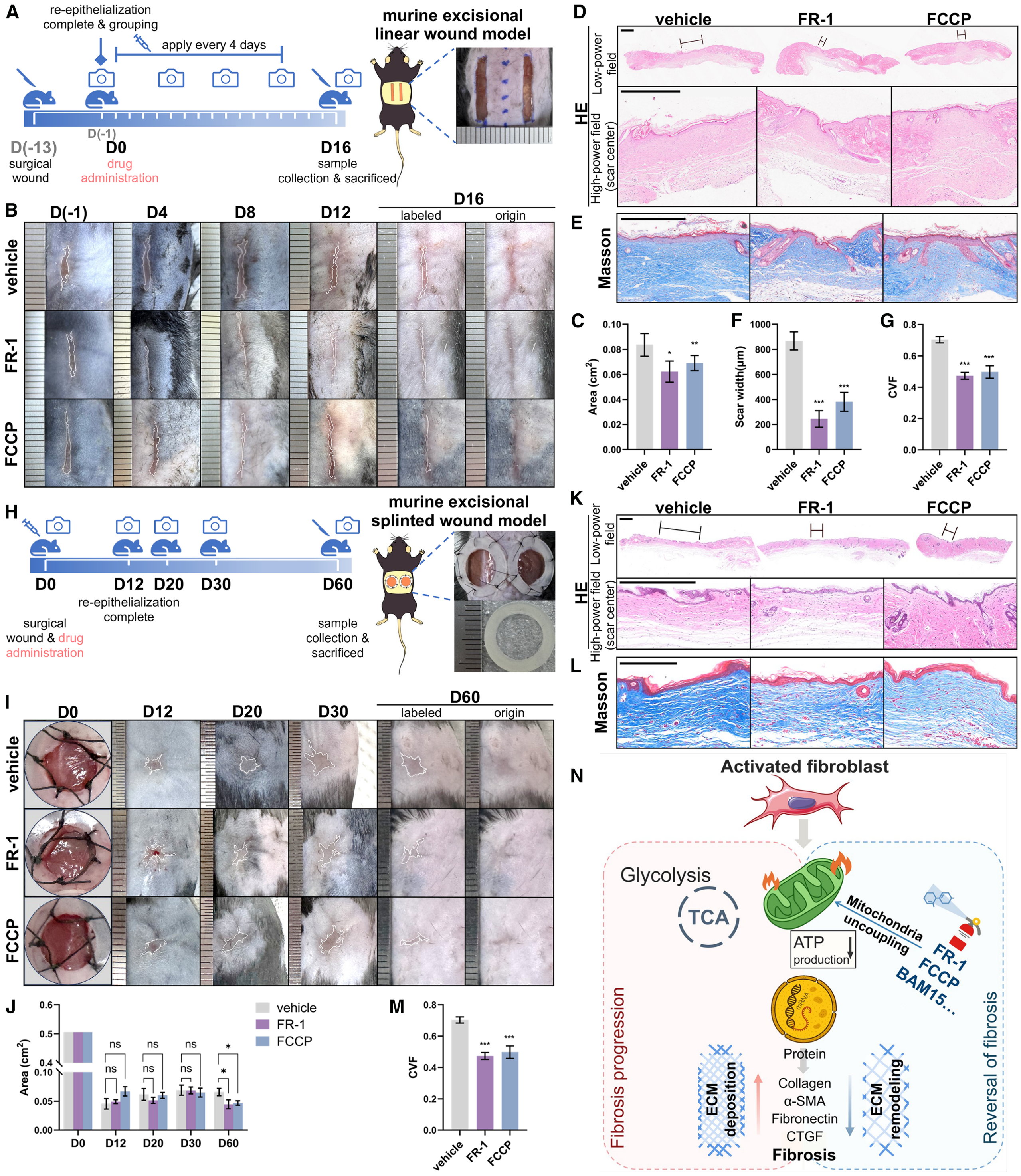

Figure 7. Mitochondrial uncoupling by FCCP also attenuates skin fibrosis

(A) Schematic and timeline for assessing mitochondrial uncoupling in the murine linear excisional wound model.

(B) Representative scar images (white boxes: scar margins) from vehicle-, FR-1-, and FCCP-treated mice at indicated time points.

(C) Scar area quantification on day 16 (n = 10 scars from 5 mice per group). Representative (D) H&E staining (black lines: scar width, scale bars, 500 μm) and (E) Masson's trichrome staining (scale bars, 500 μm) of scar tissues. Quantitative data of (F) scar width (n = 4 mice) and (G) CVF analysis (n = 5 mice).

(H) Schematic and timeline of the murine splinted excisional wound model.

(I) Representative images of wound healing and scar formation progression (D0–D60) with vehicle, FR-1, or FCCP treatment.

(legend continued on next page)

Rottlerin—a constituent of traditional herbal medicine[33]—demonstrated excellent tolerability in murine models, including anti-tumor study (20 mg/kg, 5 days a week, once daily, gavage, for 6 weeks), neuroprotection research (3–20 mg/kg, oral/ intraperitoneal [i.p.], 7 days), and other preclinical investigations, with no reported toxicity.[34,35] FR-1 was developed via phenotype-guided structural optimization of Rottlerin with minimal molecular alterations. Broad-spectrum kinase profiling (419 kinases) revealed FR-1's significantly weaker inhibitory potency versus Rottlerin, demonstrating <50% maximal inhibition (43.64% at 1 μM) across all targets (Figure 2H). This attenuated kinase engagement profile correlates with diminished off-target effects, indicating a reduced propensity for adverse pharmacological responses. Collectively, these orthogonal data streams substantiate topical FR-1's low-risk safety profile for clinical development.

This work identifies mitochondrial uncoupling as a promising target for fibrosis reversal. Our results suggest that FR-1-induced mitochondrial uncoupling drives a decrease in collagen deposition and upregulation of MMP-1 expression. These concerted actions facilitate ECM remodeling, ultimately leading to a phenotype exhibiting characteristic features of fibrosis reversal (Figure 7N). This robust attenuation of the fibrotic phenotype following FR-1 treatment prompts an examination of the potential cellular mechanisms. Theoretically, the reduction in myofibroblast burden could be driven by selective apoptosis. However, our *in vitro* and *in vivo* models did not exhibit overt cell death. Alternatively, FR-1 may induce selective proliferation inhibition of activated myofibroblasts, thereby halting the expansion of the fibrotic niche. A third possibility is the genuine reversion of myofibroblasts toward a quiescent state.[64,65] While our transcriptomic and histological evidence strongly supports a profound shift away from the pathological phenotype, definitively parsing these nuanced cellular fates—specifically the distinction between selective proliferation inhibition and true cellular reversion—requires future *in vivo* genetic lineage-tracing studies.

Regarding future mechanistic investigations, while mitochondrial uncoupling serves as the primary driver of fibrosis reversal, the exact downstream molecular targets and regulatory networks remain to be fully elucidated. For instance, ATP depletion may theoretically impair Brahma-related gene 1 (BRG1)-mediated chromatin remodeling, potentially reducing the accessibility of pro-fibrotic gene loci.[66] Furthermore, we hypothesize that FR-1 may attenuate fibrotic remodeling by influencing established metabolic-sensitive networks. This includes suppression of hypoxia-inducible factor-1α (HIF-1α) signaling, which is typically upregulated in keloids and systemic sclerosis,[67,68] and engagement of the NAD^+-dependent sirtuin (NAD^+-SIRT) axis to fine-tune mitochondrial redox homeostasis.[58,69–71] Collectively, these hypotheses highlight the complex interplay between mitochondrial function, cellular metabolism, and fibrogenesis, providing critical directions for further exploration.

Our work also demonstrates that transcriptome-based phenotypic drug development is an effective pathway for discovering promising agents. Crucial to this approach was our application of DRUG-seq2, a high-throughput transcriptional sequencing method. DRUG-seq2 provided in-depth molecular insights through transcriptome analysis instead of traditional screening assay with limited biomarkers, enabling the efficient screening of compounds with primary human keloid fibroblasts. This method significantly reduced experimental costs and time compared with traditional RNA-seq approaches requiring larger sample sizes, while ensuring robust data quality.[26] This positions transcriptomic analysis by DRUG-seq2 as a powerful tool for phenotypic drug discovery.

Limitations of the study

First, the anatomical discordance between murine and human skin presents a challenge, as rodent wound closure relies heavily on panniculus carnosus contraction. Although splinted and linear models mitigate this, they cannot fully recapitulate the tension and immunology of human keloids.[72] To bridge this gap, we used patient-derived fibroblasts, *ex vivo* explants, and PDX models. While these models showed promising anti-fibrotic trends, the PDX evidence should be interpreted with caution, as no significant degradation of pre-existing keloid collagen occurred within our time frame. FR-1 lacks the direct, acute efficacy of collagenolytic agents such as collagenase, limiting its impact on the mature scar core. However, FR-1-induced MMP-1 upregulation may facilitate gradual collagen remodeling at the active boundary between established and nascent scars over prolonged treatment. Future efficacy studies in large animal models (e.g., porcine skin) remain critical for clinical translation.

Second, our insights into efficacy and mechanisms rely largely on 2D monolayer cultures, which lack the physical stiffness and 3D structure of actual fibrotic tissue. Confirming these findings in human skin organoids or 3D constructs will be necessary to better reflect physiological conditions.[73,74]

Finally, while we provide evidence for FR-1's therapeutic potential in cardiac fibrosis (Figure S11),[57,75] our focus remains on cutaneous fibrosis. Given the systemic metabolic effects of mitochondrial uncoupling, defining the therapeutic window and safety profile in non-rodent species is imperative to rule out off-target toxicities prior to human trials.

RESOURCE AVAILABILITY

Lead contact

Requests for further information and resources should be directed to and will be fulfilled by the lead contact, Yang Zhao (yangzhao@pku.edu.cn).

Materials availability

The small molecule compound FR-1 synthesized in this study, along with the plasmids (pFU-Tet-On-GFP, pFU-Tet-On-UCP1, pFU-Tet-On-UCP2, and pFU-Tet-On-UCP3) and primary human keloid fibroblasts (HKFs) generated

(J) Quantification of wound/scar areas over time (n = 6–10 scars from 3 to 5 mice per group). Representative (K) H&E staining (scale bars, 500 μm; black lines mark scar width) and (L) Masson's trichrome staining (scale bars, 200 μm).
(M) Quantification of CVF (n = 3–4 mice).
(N) Schematic diagram illustrating the mechanism by which FR-1 reverses fibrosis through promoting mitochondrial uncoupling. Data are mean ± SEM; n represents the number of independent biological replicates. *$p < 0.05$; **$p < 0.01$; ***$p < 0.001$; ns, not statistically significant vs. vehicle by one-way ANOVA.

in this work, will be made available upon request, but we may require payment and/or a completed materials transfer agreement (MTA) if there is potential for commercial application.

Data and code availability

- The DRUG-seq2 and bulk RNA-seq data generated in this study have been deposited at the NCBI Gene Expression Omnibus (GEO) under accession numbers GEO: GSE303486, GSE303487. These data are publicly available as of the date of publication, and the accession numbers are also listed in the Key Resources Table.
- The crystallographic data for compound FR-1 generated in this study have been deposited at the Cambridge Crystallographic Data Centre (CCDC) under accession number CCDC: 2498324.
- This paper does not report original code.
- Any additional information required to reanalyze the data reported in this paper is available from the lead contact upon request.

ACKNOWLEDGMENTS

This work was supported by the National Natural Science Foundation of China (grant no. 82172206 to W.-Q.T. and grant no. 32470882 to Y.Z.). We thank Yiying Xu, Yong Wang, Tao Zhang, and Zeming Zhuang (Department of Plastic Surgery, Sir Run Run Shaw Hospital, Zhejiang University School of Medicine) for their assistance with image acquisition and data verification. We are grateful to Zhongya Song (Jiangsu Key Laboratory of Molecular Biology for Skin Diseases and STIs, Institute of Dermatology, Chinese Academy of Medical Sciences and Peking Union Medical College) for providing human keloid and hypertrophic scar tissues. We thank Donghui Wu (State Key Laboratory of Natural and Biomimetic Drugs, MOE Key Laboratory of Cell Proliferation and Differentiation, Beijing Key Laboratory of Cardiometabolic Molecular Medicine, Institute of Molecular Medicine, College of Future Technology, Peking University) for preparing myocardial infarction (MI) mouse models. We thank Xiaoli Hong and Chao Bi (Core Facilities, Zhejiang University School of Medicine) for their technical support.

AUTHOR CONTRIBUTIONS

Conceptualization, Y.Z. and W.-Q.T.; methodology, C.-Y.C., R.X., M.M., Jia W., Y.L., Jin W., and H.F.; investigation, C.-Y.C., R.X., Z.-R.W., X.-C.Z., and Jia W.; formal analysis, C.-Y.C., R.X., M.M., Y.W., J.C., and H.J.; visualization, C.-Y.C. and R.X.; resources, Y.Y., H.B., and X.-Y.L.; data curation, C.-Y.C. and R.X.; funding acquisition, W.-Q.T. and Y.Z.; project administration, Y.Z. and W.-Q.T.; supervision, Y.Z., W.-Q.T., and H.B.; writing – original draft, C.-Y.C. and R.X.; writing – review and editing, C.-Y.C., R.X., Y.Z., and W.-Q.T.

DECLARATION OF INTERESTS

The authors are filing a patent associated with this study. Y.Z. is a shareholder of Plastech, as a founder and CSO of Plastech. M.M., Jia W., J.C., and Y.L. were employees of Plastech.

DECLARATION OF GENERATIVE AI AND AI-ASSISTED TECHNOLOGIES IN THE WRITING PROCESS

During the preparation of this work, the author(s) used Gemini (Google) in order to improve the readability and language of the manuscript. After using this tool or service, the author(s) reviewed and edited the content as needed and take(s) full responsibility for the content of the publication.

STAR★METHODS

Detailed methods are provided in the online version of this paper and include the following:

- KEY RESOURCES TABLE
- EXPERIMENTAL MODEL AND STUDY PARTICIPANT DETAILS
  - Patient samples
  - Experimental animals
  - Cell lines
- METHOD DETAILS
  - Isolation and culture of human keloid fibroblasts (HKFs)
  - Small molecule screen
  - DRUG-seq2 library preparation and data analysis
  - RNA extraction and RT-qPCR
  - Western blotting
  - Structural optimization and SAR strategy
  - Kinase activity and protein interaction assays
  - Single-crystal X-ray diffraction analysis
  - Bulk RNA-seq data analysis
  - Mitochondrial morphology analysis
  - Measurement of mitochondrial membrane potential
  - ATP/ADP mass spectrometry detection
  - Measurement of mitochondrial respiration
  - Flow cytometry detection of cell apoptosis
  - Cell senescence β-galactosidase staining
  - Preparation of FR-1 formulations
  - Transdermal permeability and HPLC analysis
  - Linear excisional wound model
  - Murine splinted excisional wound model
  - *Ex vivo* keloid explant culture and treatment
  - Patient-derived xenograft (PDX) model
  - Standardized photographic protocol
  - Plasmid construction and transfection
  - *In vitro* permeation testing (IVPT) and quantification
  - Establishment of MI mouse model of myocardial infarction
  - Isolation and culture of primary cardiac fibroblasts
- QUANTIFICATION AND STATISTICAL ANALYSIS

SUPPLEMENTAL INFORMATION

Supplemental information can be found online at https://doi.org/10.1016/j.xcrm.2026.102821.

Received: October 15, 2025
Revised: March 10, 2026
Accepted: April 20, 2026
Published: May 19, 2026

REFERENCES

1. Henderson, N.C., Rieder, F., and Wynn, T.A. (2020). Fibrosis: from mechanisms to medicines. Nature *587*, 555–566. https://doi.org/10.1038/s41586-020-2938-9.
2. Gurtner, G.C., Werner, S., Barrandon, Y., and Longaker, M.T. (2008). Wound repair and regeneration. Nature *453*, 314–321. https://doi.org/10.1038/nature07039.
3. Talbott, H.E., Mascharak, S., Griffin, M., Wan, D.C., and Longaker, M.T. (2022). Wound healing, fibroblast heterogeneity, and fibrosis. Cell Stem Cell *29*, 1161–1180. https://doi.org/10.1016/j.stem.2022.07.006.
4. Mascharak, S., desJardins-Park, H.E., Davitt, M.F., Griffin, M., Borrelli, M.R., Moore, A.L., Chen, K., Duoto, B., Chinta, M., Foster, D.S., et al. (2021). Preventing Engrailed-1 activation in fibroblasts yields wound regeneration without scarring. Science *372*, eaba2374. https://doi.org/10.1126/science.aba2374.
5. Jeschke, M.G., Wood, F.M., Middelkoop, E., Bayat, A., Teot, L., Ogawa, R., and Gauglitz, G.G. (2023). Scars. Nat. Rev. Dis. Primers *9*, 64. https://doi.org/10.1038/s41572-023-00474-x.
6. Frech, F.S., Hernandez, L., Urbonas, R., Zaken, G.A., Dreyfuss, I., and Nouri, K. (2023). Hypertrophic Scars and Keloids: Advances in Treatment and Review of Established Therapies. Am. J. Clin. Dermatol. *24*, 225–245. https://doi.org/10.1007/s40257-022-00744-6.

7. Reid, D., Malak, S., Khadka, M., Hanna, R., Pharr, T., Wyant, W.A., and Albers, S. (2025). Keloids and hypertrophic scars in individuals with darker Fitzpatrick skin types: a systematic review of treatment efficacy and quality of life outcomes. Arch. Dermatol. Res. *317*, 795. https://doi.org/10.1007/s00403-025-04292-x.

8. Srivastava, S., Patil, A.N., Prakash, C., and Kumari, H. (2017). Comparison of Intralesional Triamcinolone Acetonide, 5-Fluorouracil, and Their Combination for the Treatment of Keloids. Adv. Wound Care *6*, 393–400. https://doi.org/10.1089/wound.2017.0741.

9. Camarasa, J.G. (2001). Contact allergy to corticosteroids: a challenge for dermatologists. J. Eur. Acad. Dermatol. Venereol. *15*, 295–297.

10. Jabłońska-Trypuć, A., Pankiewicz, W., Wołejko, E., Sokołowska, G., Estévez, J., Sogorb, M.A., and Wydro, U. (2024). Human Skin Fibroblasts as an In Vitro Model Illustrating Changes in Collagen Levels and Skin Cell Migration Under the Influence of Selected Plant Hormones. Bioengineering *11*, 1188. https://doi.org/10.3390/bioengineering11121188.

11. Grigorieva, O., Basalova, N., Dyachkova, U., Novoseletskaya, E., Vigovskii, M., Arbatskiy, M., Kulebyakina, M., and Efimenko, A. (2024). Modeling the profibrotic microenvironment in vitro: Model validation. Biochem. Biophys. Res. Commun. *733*, 150574. https://doi.org/10.1016/j.bbrc.2024.150574.

12. Dupont, S., Morsut, L., Aragona, M., Enzo, E., Giulitti, S., Cordenonsi, M., Zanconato, F., Le Digabel, J., Forcato, M., Bicciato, S., et al. (2011). Role of YAP/TAZ in mechanotransduction. Nature *474*, 179–183. https://doi.org/10.1038/nature10137.

13. Toyama, T., Looney, A.P., Baker, B.M., Stawski, L., Haines, P., Simms, R., Szymaniak, A.D., Varelas, X., and Trojanowska, M. (2018). Therapeutic Targeting of TAZ and YAP by Dimethyl Fumarate in Systemic Sclerosis Fibrosis. J. Invest. Dermatol. *138*, 78–88. https://doi.org/10.1016/j.jid.2017.08.024.

14. Deng, C.C., Hu, Y.F., Zhu, D.H., Cheng, Q., Gu, J.J., Feng, Q.L., Zhang, L.X., Xu, Y.P., Wang, D., Rong, Z., and Yang, B. (2021). Single-cell RNA-seq reveals fibroblast heterogeneity and increased mesenchymal fibroblasts in human fibrotic skin diseases. Nat. Commun. *12*, 3709. https://doi.org/10.1038/s41467-021-24110-y.

15. Younesi, F.S., Miller, A.E., Barker, T.H., Rossi, F.M.V., and Hinz, B. (2024). Fibroblast and myofibroblast activation in normal tissue repair and fibrosis. Nat. Rev. Mol. Cell Biol. *25*, 617–638. https://doi.org/10.1038/s41580-024-00716-0.

16. Rangarajan, S., Bone, N.B., Zmijewska, A.A., Jiang, S., Park, D.W., Bernard, K., Locy, M.L., Ravi, S., Deshane, J., Mannon, R.B., et al. (2018). Metformin reverses established lung fibrosis in a bleomycin model. Nat. Med. *24*, 1121–1127. https://doi.org/10.1038/s41591-018-0087-6.

17. Lv, Y.F., Xie, C.S., Liu, Z.X., Kang, M.D., Liu, Y., Liao, Z.Q., Ji, Y.L., Zhao, R., Li, Y.S., Wei, X.Y., et al. (2024). Sevelamer reverses liver fibrosis by deactivation of hepatic stellate cells. Biochem. Pharmacol. *222*, 116121. https://doi.org/10.1016/j.bcp.2024.116121.

18. Agarwal, P., Huckle, J., Newman, J., and Reid, D.L. (2022). Trends in small molecule drug properties: A developability molecule assessment perspective. Drug Discov. Today *27*, 103366. https://doi.org/10.1016/j.drudis.2022.103366.

19. Bai, Y., Yang, Z., Xu, X., Ding, W., Qi, J., Liu, F., Wang, X., Zhou, B., Zhang, W., Zhuang, X., et al. (2023). Direct chemical induction of hepatocyte-like cells with capacity for liver repopulation. Hepatology *77*, 1550–1565. https://doi.org/10.1002/hep.32686.

20. Luo, W., Gu, Y., Fu, S., Wang, J., Zhang, J., and Wang, Y. (2023). Emerging opportunities to treat idiopathic pulmonary fibrosis: Design, discovery, and optimizations of small-molecule drugs targeting fibrogenic pathways. Eur. J. Med. Chem. *260*, 115762. https://doi.org/10.1016/j.ejmech.2023.115762.

21. Paine, P.T., Nguyen, A., and Ocampo, A. (2024). Partial cellular reprogramming: A deep dive into an emerging rejuvenation technology. Aging Cell *23*, e14039. https://doi.org/10.1111/acel.14039.

22. Avelar, R.A., Palmer, D., Kulaga, A.Y., and Fuellen, G. (2025). Conserved biological processes in partial cellular reprogramming: Relevance to aging and rejuvenation. Ageing Res. Rev. *108*, 102737. https://doi.org/10.1016/j.arr.2025.102737.

23. Vásquez-Pacheco, E., Marega, M., Lingampally, A., Fassy, J., Truchi, M., Goth, K., Trygub, L., Taghizadeh, S., Bartkuhn, M., Alexopoulos, I., et al. (2024). Highlighting fibroblast plasticity in lung fibrosis: the WI-38 cell line as a model for investigating the myofibroblast and lipofibroblast switch. Theranostics *14*, 3603–3622. https://doi.org/10.7150/thno.93519.

24. Plikus, M.V., Guerrero-Juarez, C.F., Ito, M., Li, Y.R., Dedhia, P.H., Zheng, Y., Shao, M., Gay, D.L., Ramos, R., Hsi, T.C., et al. (2017). Regeneration of fat cells from myofibroblasts during wound healing. Science *355*, 748–752. https://doi.org/10.1126/science.aai8792.

25. Jiang, W., Pang, X., Ha, P., Li, C., Chang, G.X., Zhang, Y., Bossong, L.A., Ting, K., Soo, C., and Zheng, Z. (2025). Fibromodulin selectively accelerates myofibroblast apoptosis in cutaneous wounds by enhancing interleukin 1β signaling. Nat. Commun. *16*, 3499. https://doi.org/10.1038/s41467-025-58906-z.

26. Li, J., Bai, Y., Liu, Y., Song, Z., Yang, Y., and Zhao, Y. (2023). Transcriptome-based chemical screens identify CDK8 as a common barrier in multiple cell reprogramming systems. Cell Rep. *42*, 112566. https://doi.org/10.1016/j.celrep.2023.112566.

27. Juhl, P., Bondesen, S., Hawkins, C.L., Karsdal, M.A., Bay-Jensen, A.C., Davies, M.J., and Siebuhr, A.S. (2020). Dermal fibroblasts have different extracellular matrix profiles induced by TGF-β, PDGF and IL-6 in a model for skin fibrosis. Sci. Rep. *10*, 17300. https://doi.org/10.1038/s41598-020-74179-6.

28. Mori, Y., Ishida, W., Bhattacharyya, S., Li, Y., Platanias, L.C., and Varga, J. (2004). Selective inhibition of activin receptor-like kinase 5 signaling blocks profibrotic transforming growth factor beta responses in skin fibroblasts. Arthritis Rheum. *50*, 4008–4021. https://doi.org/10.1002/art.20658.

29. Huang, J., Beyer, C., Palumbo-Zerr, K., Zhang, Y., Ramming, A., Distler, A., Gelse, K., Distler, O., Schett, G., Wollin, L., and Distler, J.H.W. (2016). Nintedanib inhibits fibroblast activation and ameliorates fibrosis in preclinical models of systemic sclerosis. Ann. Rheum. Dis. *75*, 883–890. https://doi.org/10.1136/annrheumdis-2014-207109.

30. Saad, K.M., Salles, É.L., Naeini, S.E., Baban, B., Abdelmageed, M.E., Abdelaziz, R.R., Suddek, G.M., and Elmarakby, A.A. (2024). Reno-protective effect of protocatechuic acid is independent of sex-related differences in murine model of UUO-induced kidney injury. Pharmacol. Rep. *76*, 98–111. https://doi.org/10.1007/s43440-023-00565-2.

31. Wang, W., Qu, M., Xu, L., Wu, X., Gao, Z., Gu, T., Zhang, W., Ding, X., Liu, W., and Chen, Y.L. (2016). Sorafenib exerts an anti-keloid activity by antagonizing TGF-β/Smad and MAPK/ERK signaling pathways. J. Mol. Med. *94*, 1181–1194. https://doi.org/10.1007/s00109-016-1430-3.

32. Lee, W.J., Lee, J.L., Chang, S.E., Lee, M.W., Kang, Y.K., Choi, J.H., Moon, K.C., and Koh, J.K. (2009). Cutaneous adverse effects in patients treated with the multitargeted kinase inhibitors sorafenib and sunitinib. Br. J. Dermatol. *161*, 1045–1051. https://doi.org/10.1111/j.1365-2133.2009.09290.x.

33. Gangwar, M., Goel, R.K., and Nath, G. (2014). Mallotus philippinensis Muell. Arg (Euphorbiaceae): ethnopharmacology and phytochemistry review. Biomed Res. Int. *2014*, 213973. https://doi.org/10.1155/2014/213973.

34. Zhang, D., Anantharam, V., Kanthasamy, A., and Kanthasamy, A.G. (2007). Neuroprotective effect of protein kinase C delta inhibitor rottlerin in cell culture and animal models of Parkinson's disease. J. Pharmacol. Exp. Ther. *322*, 913–922. https://doi.org/10.1124/jpet.107.124669.

35. Huang, M., Tang, S.N., Upadhyay, G., Marsh, J.L., Jackman, C.P., Srivastava, R.K., and Shankar, S. (2014). Rottlerin suppresses growth of human pancreatic tumors in nude mice, and pancreatic cancer cells isolated from Kras(G12D) mice. Cancer Lett. *353*, 32–40. https://doi.org/10.1016/j.canlet.2014.06.021.

36. Hong, K.K.C., Ho, K.K.K., Bhadbhade, M., Ball, G.E., Black, D.S., and Kumar, N. (2019). The Mosaic of Rottlerin: The Sequel. J. Nat. Prod. *82*, 1190–1199. https://doi.org/10.1021/acs.jnatprod.8b00917.

37. Tan, W.Q., Fang, Q.Q., Shen, X.Z., Giani, J.F., Zhao, T.V., Shi, P., Zhang, L.Y., Khan, Z., Li, Y., Li, L., et al. (2018). Angiotensin-converting enzyme inhibitor works as a scar formation inhibitor by down-regulating Smad and TGF-β-activated kinase 1 (TAK1) pathways in mice. Br. J. Pharmacol. *175*, 4239–4252. https://doi.org/10.1111/bph.14489.

38. Walsh, L.A., Wu, E., Pontes, D., Kwan, K.R., Poondru, S., Miller, C.H., and Kundu, R.V. (2023). Keloid treatments: an evidence-based systematic review of recent advances. Syst. Rev. *12*, 42. https://doi.org/10.1186/s13643-023-02192-7.

39. Savitri, C., Ha, S.S., Kwon, J.W., Kim, S.H., Kim, Y.M., Park, H.M., Kwon, H., Ji, M.J., and Park, K. (2024). Human Fibroblast-Derived Matrix Hydrogel Accelerates Regenerative Wound Remodeling Through the Interactions with Macrophages. Adv. Sci. *11*, e2305852. https://doi.org/10.1002/advs.202305852.

40. Zinchuk, V., Zinchuk, O., and Okada, T. (2007). Quantitative colocalization analysis of multicolor confocal immunofluorescence microscopy images: pushing pixels to explore biological phenomena. Acta Histochem. Cytochem. *40*, 101–111. https://doi.org/10.1267/ahc.07002.

41. Lin-Hui, L., Yuan-Yuan, Z., Ming-Yu, L., Xu-Dong, H., Yin-Jia, D., Yue, Z., Yang-Hong-Hong, F., Ai-Fen, C., Xu-Dong, Z., Zheng-Li, C., and Jian, J. (2024). Recombinant Human Collagen Type III Improves Hypertrophic Scarring by Regulating the Ratio of Type I/III Collagen. J. Burn Care Res. *45*, 1269–1273. https://doi.org/10.1093/jbcr/irae040.

42. Gao, J., Guo, Z., Zhang, Y., Liu, Y., Xing, F., Wang, J., Luo, X., Kong, Y., and Zhang, G. (2023). Age-related changes in the ratio of Type I/III collagen and fibril diameter in mouse skin. Regen. Biomater. *10*, rbac110. https://doi.org/10.1093/rb/rbac110.

43. Boudaoud, A., Burian, A., Borowska-Wykręt, D., Uyttewaal, M., Wrzalik, R., Kwiatkowska, D., and Hamant, O. (2014). FibrilTool, an ImageJ plug-in to quantify fibrillar structures in raw microscopy images. Nat. Protoc. *9*, 457–463. https://doi.org/10.1038/nprot.2014.024.

44. Walmsley, G.G., Maan, Z.N., Wong, V.W., Duscher, D., Hu, M.S., Zielins, E.R., Wearda, T., Muhonen, E., McArdle, A., Tevlin, R., et al. (2015). Scarless wound healing: chasing the holy grail. Plast. Reconstr. Surg. *135*, 907–917. https://doi.org/10.1097/prs.0000000000000972.

45. Godin, B., and Touitou, E. (2007). Transdermal skin delivery: predictions for humans from in vivo, ex vivo and animal models. Adv. Drug Deliv. Rev. *59*, 1152–1161. https://doi.org/10.1016/j.addr.2007.07.004.

46. Galiano, R.D., Michaels, J., 5th, Dobryansky, M., Levine, J.P., and Gurtner, G.C. (2004). Quantitative and reproducible murine model of excisional wound healing. Wound Repair Regen. *12*, 485–492. https://doi.org/10.1111/j.1067-1927.2004.12404.x.

47. Soltoff, S.P. (2007). Rottlerin: an inappropriate and ineffective inhibitor of PKCdelta. Trends Pharmacol. Sci. *28*, 453–458. https://doi.org/10.1016/j.tips.2007.07.003.

48. Ojha, D., Winkler, C.W., Leung, J.M., Woods, T.A., Chen, C.Z., Nair, V., Taylor, K., Yeh, C.D., Tawa, G.J., Larson, C.L., et al. (2021). Rottlerin inhibits La Crosse virus-induced encephalitis in mice and blocks release of replicating virus from the Golgi body in neurons. Nat. Microbiol. *6*, 1398–1409. https://doi.org/10.1038/s41564-021-00968-y.

49. Lee, T.H., Chen, J.L., Liu, P.S., Tsai, M.M., Wang, S.J., and Hsieh, H.L. (2020). Rottlerin, a natural polyphenol compound, inhibits upregulation of matrix metalloproteinase-9 and brain astrocytic migration by reducing PKC-δ-dependent ROS signal. J. Neuroinflammation *17*, 177. https://doi.org/10.1186/s12974-020-01859-5.

50. Zhu, J., Wang, Y., Hu, Q., Yuan, R., and Ye, J. (2020). Rottlerin acts as a therapeutic in primary open-angle glaucoma by targeting the trabecular meshwork via activation of Rap1 signaling. Pharmacol. Res. *159*, 104780. https://doi.org/10.1016/j.phrs.2020.104780.

51. Manhas, D., Bhatt, S., Rai, G., Kumar, V., Bharti, S., Dhiman, S., Jain, S.K., Sharma, D.K., Ojha, P.K., Gandhi, S.G., et al. (2023). Rottlerin renders a selective and highly potent CYP2C8 inhibition to impede EET formation for implication in cancer therapy. Chem. Biol. Interact. *380*, 110524. https://doi.org/10.1016/j.cbi.2023.110524.

52. Springael, C., Thomas, S., Rahmouni, S., Vandamme, A., Goldman, M., Willems, F., and Vosters, O. (2007). Rottlerin inhibits human T cell responses. Biochem. Pharmacol. *73*, 515–525. https://doi.org/10.1016/j.bcp.2006.10.034.

53. Anastassiadis, T., Deacon, S.W., Devarajan, K., Ma, H., and Peterson, J.R. (2011). Comprehensive assay of kinase catalytic activity reveals features of kinase inhibitor selectivity. Nat. Biotechnol. *29*, 1039–1045. https://doi.org/10.1038/nbt.2017.

54. Vasta, J.D., Corona, C.R., Wilkinson, J., Zimprich, C.A., Hartnett, J.R., Ingold, M.R., Zimmerman, K., Machleidt, T., Kirkland, T.A., Huwiler, K.G., et al. (2018). Quantitative, Wide-Spectrum Kinase Profiling in Live Cells for Assessing the Effect of Cellular ATP on Target Engagement. Cell Chem. Biol. *25*, 206–214.e11. https://doi.org/10.1016/j.chembiol.2017.10.010.

55. Tavares, C.D.J., Devkota, A.K., Dalby, K.N., and Cho, E.J. (2016). Application of Eukaryotic Elongation Factor-2 Kinase (eEF-2K) for Cancer Therapy: Expression, Purification, and High-Throughput Inhibitor Screening. In Kinase Screening and Profiling: Methods and Protocols, H. Zegzouti and S.A. Goueli, eds. (Springer), pp. 19–33.

56. Ma, J., Hou, Y., Xia, J., Zhu, X., and Wang, Z.P. (2018). Tumor suppressive role of rottlerin in cancer therapy. Am. J. Transl. Res. *10*, 3345–3356.

57. Dantas, W.S., Zunica, E.R.M., Heintz, E.C., Vandanmagsar, B., Floyd, Z.E., Yu, Y., Fujioka, H., Hoppel, C.L., Belmont, K.P., Axelrod, C.L., and Kirwan, J.P. (2022). Mitochondrial uncoupling attenuates sarcopenic obesity by enhancing skeletal muscle mitophagy and quality control. J. Cachexia Sarcopenia Muscle *13*, 1821–1836. https://doi.org/10.1002/jcsm.12982.

58. Demine, S., Renard, P., and Arnould, T. (2019). Mitochondrial Uncoupling: A Key Controller of Biological Processes in Physiology and Diseases. Cells *8*, 795. https://doi.org/10.3390/cells8080795.

59. Walker Wadsworth, E., Duckett, K.A., and Patel, K.G. (2024). High-Grade Pleomorphic Sarcoma in Postauricular Keloid Scar After Surgical Excision and Radiation. Laryngoscope *134*, 3143–3145. https://doi.org/10.1002/lary.31337.

60. Thaçi, D., Gooderham, M., Lovato, P., Madsen, D.E., Soehoel, A., and Bissonnette, R. (2025). Systemic exposure and bioavailability of delgocitinib cream in adults with moderate to severe Chronic Hand Eczema. J. Eur. Acad. Dermatol. Venereol. *39*, 1612–1621. https://doi.org/10.1111/jdv.20777.

61. Jett, J.E., McLaughlin, M., Lee, M.S., Parish, L.C., DuBois, J., Raoof, T.J., Tabolt, G., Wilson, T., Somerville, M.C., DellaMaestra, W., and Piscitelli, S.C. (2022). Tapinarof Cream 1% for Extensive Plaque Psoriasis: A Maximal Use Trial on Safety, Tolerability, and Pharmacokinetics. Am. J. Clin. Dermatol. *23*, 83–91. https://doi.org/10.1007/s40257-021-00641-4.

62. Lou, F., Xu, Z., Bai, J., Zhao, X., Cui, L., Li, Q., and Wang, H. (2024). Identification and pre-clinical investigation of 3-O-cyclohexanecarbonyl-11-keto-β-boswellic acid as a drug for external use to treat psoriasis. Br. J. Pharmacol. *181*, 1290–1307. https://doi.org/10.1111/bph.16253.

63. Veloso, C., Videira, R.A., Andrade, P.B., Cardoso, C., and Vitorino, C. (2022). In vivo methodologies to assist preclinical development of topical fixed-dose combinations for pain management. Int. J. Pharm. *616*, 121530. https://doi.org/10.1016/j.ijpharm.2022.121530.

64. Lin, H., Wang, X., Chung, M., Cai, S., and Pan, Y. (2025). Direct fibroblast reprogramming: an emerging strategy for treating organic fibrosis. J. Transl. Med. *23*, 240. https://doi.org/10.1186/s12967-024-06060-3.

65. Niti, A., Kouzi-Koliakou, K., and Michopoulou, A. (2026). Fibroblast Lineage Switching as the Developmental Origin of Scarring and Target for Regenerative Healing. Biology *15*, 409. https://doi.org/10.3390/biology15050409.

CellPress
OPEN ACCESS

66. Ailiken, G., Kitamura, K., Hoshino, T., Satoh, M., Tanaka, N., Minamoto, T., Rahmutulla, B., Kobayashi, S., Kano, M., Tanaka, T., et al. (2020). Post-transcriptional regulation of BRG1 by FIRΔexon2 in gastric cancer. Oncogenesis *9*, 26. https://doi.org/10.1038/s41389-020-0205-4.

67. Maciejewska, M., Sikora, M., Stec, A., Zaremba, M., Maciejewski, C., Pawlik, K., and Rudnicka, L. (2023). Hypoxia-Inducible Factor-1α (HIF-1α) as a Biomarker for Changes in Microcirculation in Individuals with Systemic Sclerosis. Dermatol. Ther. *13*, 1549–1560. https://doi.org/10.1007/s13555-023-00952-w.

68. Vincent, A.S., Phan, T.T., Mukhopadhyay, A., Lim, H.Y., Halliwell, B., and Wong, K.P. (2008). Human skin keloid fibroblasts display bioenergetics of cancer cells. J. Invest. Dermatol. *128*, 702–709. https://doi.org/10.1038/sj.jid.5701107.

69. Higashi, T., Friedman, S.L., and Hoshida, Y. (2017). Hepatic stellate cells as key target in liver fibrosis. Adv. Drug Deliv. Rev. *121*, 27–42. https://doi.org/10.1016/j.addr.2017.05.007.

70. Cheng, Z., and Ristow, M. (2013). Mitochondria and metabolic homeostasis. Antioxid. Redox Signal. *19*, 240–242. https://doi.org/10.1089/ars.2013.5255.

71. Peng, F., Liao, M., Jin, W., Liu, W., Li, Z., Fan, Z., Zou, L., Chen, S., Zhu, L., Zhao, Q., et al. (2024). 2-APQC, a small-molecule activator of Sirtuin-3 (SIRT3), alleviates myocardial hypertrophy and fibrosis by regulating mitochondrial homeostasis. Signal Transduct. Target. Ther. *9*, 133. https://doi.org/10.1038/s41392-024-01816-1.

72. Mony, M.P., Harmon, K.A., Hess, R., Dorafshar, A.H., and Shafikhani, S.H. (2023). An Updated Review of Hypertrophic Scarring. Cells *12*, 678. https://doi.org/10.3390/cells12050678.

73. Ebner-Peking, P., Krisch, L., Wolf, M., Hochmann, S., Hoog, A., Vári, B., Muigg, K., Poupardin, R., Scharler, C., Schmidhuber, S., et al. (2021). Self-assembly of differentiated progenitor cells facilitates spheroid human skin organoid formation and planar skin regeneration. Theranostics *11*, 8430–8447. https://doi.org/10.7150/thno.59661.

74. Wang, W., Liu, P., Zhu, W., Li, T., Wang, Y., Wang, Y., Li, J., Ma, J., and Leng, L. (2025). Skin organoid transplantation promotes tissue repair with scarless in frostbite. Protein Cell *16*, 240–259. https://doi.org/10.1093/procel/pwae055.

75. Grundlingh, J., Dargan, P.I., El-Zanfaly, M., and Wood, D.M. (2011). 2,4-dinitrophenol (DNP): a weight loss agent with significant acute toxicity and risk of death. J. Med. Toxicol. *7*, 205–212. https://doi.org/10.1007/s13181-011-0162-6.

76. Dobin, A., Davis, C.A., Schlesinger, F., Drenkow, J., Zaleski, C., Jha, S., Batut, P., Chaisson, M., and Gingeras, T.R. (2013). STAR: ultrafast universal RNA-seq aligner. Bioinformatics *29*, 15–21. https://doi.org/10.1093/bioinformatics/bts635.

77. Hänzelmann, S., Castelo, R., and Guinney, J. (2013). GSVA: gene set variation analysis for microarray and RNA-seq data. BMC Bioinformatics *14*, 7. https://doi.org/10.1186/1471-2105-14-7.

78. Liao, Y., Smyth, G.K., and Shi, W. (2014). featureCounts: an efficient general purpose program for assigning sequence reads to genomic features. Bioinformatics *30*, 923–930. https://doi.org/10.1093/bioinformatics/btt656.

79. Love, M.I., Huber, W., and Anders, S. (2014). Moderated estimation of fold change and dispersion for RNA-seq data with DESeq2. Genome Biol. *15*, 550. https://doi.org/10.1186/s13059-014-0550-8.

80. Wu, T., Hu, E., Xu, S., Chen, M., Guo, P., Dai, Z., Feng, T., Zhou, L., Tang, W., Zhan, L., et al. (2021). clusterProfiler 4.0: A universal enrichment tool for interpreting omics data. Innovation (Camb) *2*, 100141. https://doi.org/10.1016/j.xinn.2021.100141.

81. Ashburner, M., Ball, C.A., Blake, J.A., Botstein, D., Butler, H., Cherry, J.M., Davis, A.P., Dolinski, K., Dwight, S.S., Eppig, J.T., et al. (2000). Gene ontology: tool for the unification of biology. The Gene Ontology Consortium. Nat. Genet. *25*, 25–29. https://doi.org/10.1038/75556.

82. Subramanian, A., Tamayo, P., Mootha, V.K., Mukherjee, S., Ebert, B.L., Gillette, M.A., Paulovich, A., Pomeroy, S.L., Golub, T.R., Lander, E.S., and Mesirov, J.P. (2005). Gene set enrichment analysis: a knowledge-based approach for interpreting genome-wide expression profiles. Proc. Natl. Acad. Sci. USA *102*, 15545–15550. https://doi.org/10.1073/pnas.0506580102.

83. Kanehisa, M., and Goto, S. (2000). KEGG: kyoto encyclopedia of genes and genomes. Nucleic Acids Res. *28*, 27–30. https://doi.org/10.1093/nar/28.1.27.

84. Spandidos, A., Wang, X., Wang, H., and Seed, B. (2010). PrimerBank: a resource of human and mouse PCR primer pairs for gene expression detection and quantification. Nucleic Acids Res. *38*, D792–D799. https://doi.org/10.1093/nar/gkp1005.

85. Dolomanov, O., Bourhis, L., Gildea, R., Howard, J., and Puschmann, H. (2009). OLEX2: A complete structure solution, refinement and analysis program. J. Appl. Cryst. J. Appl. Cryst *42*, 339–341.

86. Sheldrick, G.M. (2008). A short history of SHELX. Acta Crystallogr A *64*, 112–122. https://doi.org/10.1107/s0108767307043930.

87. Sheldrick, G.M. (2015). Crystal structure refinement with SHELXL. Acta Crystallogr. C Struct. Chem. *71*, 3–8. https://doi.org/10.1107/s2053229614024218.

STAR★METHODS

KEY RESOURCES TABLE

REAGENT or RESOURCE	SOURCE	IDENTIFIER
**Antibodies**		
Rabbit anti-COL1A1	Abcam	Cat# ab138492; RRID:AB_2861258
Rabbit anti-MMP1	Abcam	Cat# ab134184; RRID:AB_3094798
Mouse anti-GAPDH	Abmart	Cat# M20006S; RRID:AB_2737054
HRP-conjugated Goat Anti-Rabbit IgG	Abmart	Cat# M21002S; RRID:AB_2713951
HRP-conjugated Goat Anti-Mouse IgG	Abmart	Cat# M21001S; RRID:AB_2713950
Rabbit anti-α-SMA (for IHC)	Abcam	Cat# ab5694; RRID:AB_2223021
Rabbit anti-α-SMA (for IF)	Cell Signaling Technology	Cat# 19245; RRID:AB_2734735
Rabbit anti-TGF-β1	Proteintech	Cat# 26155-1-AP; RRID:AB_3085844
Mouse anti-β-catenin	Proteintech	Cat# 66379-1-Ig; RRID:AB_2857358
Biotin-conjugated Goat Anti-Rabbit IgG(H + L)	Proteintech	Cat# SA00004-2; RRID:AB_2890944
Biotin-conjugated Goat Anti-Mouse IgG(H + L)	Proteintech	Cat# SA00004-1; RRID:AB_2890900
CoraLite488-conjugated Goat Anti-Rabbit IgG(H + L)	Proteintech	Cat# SA00013-2; RRID:AB_2797132
CoraLite594-conjugated Goat Anti-Mouse IgG(H + L)	Proteintech	Cat# SA00013-3; RRID:AB_2797133
Acetonitrile (HPLC grade)	FULLTIME	Cat# 6368BU31
Methanol (HPLC grade)	FULLTIME	Cat# 6508CQ03
Trifluoroacetic acid (TFA)	CNW	Cat# X2191C025
**Bacterial and virus strains**		
TransStbl3 Chemically Competent Cell	TransGen Biotech	Cat# CD521-01
**Biological samples**		
Adult human hypertrophic scar/keloid tissues	Chinese Academy of Medical Sciences and Peking Union Medical College and Peking University Third Hospital	N/A
Full-thickness dorsal skin from miniature pigs (Female, 3 months old)	Sundia MediTech (Shanghai) Co., Ltd.	N/A
**Chemicals, peptides, and recombinant proteins**		
Dispase II	Sigma	Cat# D4693-1G
Collagenase I	Gibco	Cat# 17100-017
Collagenase II	Gibco	Cat# 17101015
DNase I	Yeasen	Cat# 10607ES15
Y-27632 (ROCK inhibitor)	Selleck	Cat# S6390
Fetal Bovine Serum (FBS)	VISTECH	Cat# SE100-B
High Glucose DMEM	HyClone	Cat# SH30022.01
Penicillin-Streptomycin	Gibco	Cat# 15140122
ITS (Insulin-Transferrin-Selenium)	Beyotime	Cat# C0341-10mL
Red Blood Cell Lysis Buffer	Solarbio	Cat# R1010
Human bFGF	Dogesce	Cat# TP750002
Sorafenib	TargetMol	Cat# T0093L
Vitamin A	TargetMol	Cat# T1183
Protocatechuic acid	TargetMol	Cat# T0562
SB431542	TargetMol	Cat# T1726

(*Continued on next page*)

CellPress OPEN ACCESS

Continued

REAGENT or RESOURCE	SOURCE	IDENTIFIER
Rosiglitazone	TargetMol	Cat# T0334
Nintedanib	TargetMol	Cat# T1777
L-Ascorbic acid	TargetMol	Cat# T0928
DB1976	TargetMol	Cat# T10964
ICG-001	TargetMol	Cat# T6113
Bosentan	TargetMol	Cat# T6264
GW788388	TargetMol	Cat# T1800
JQ1	TargetMol	Cat# T2110
Repsox	TargetMol	Cat# T6337
Rottlerin	TargetMol	Cat# T16791
Triamcinolone	TargetMol	Cat# T0798
Imatinib	TargetMol	Cat# T6230
CAY10585	TargetMol	Cat# T3494
5-FU	TargetMol	Cat# T0984
SAR-100842	TargetMol	Cat# T4521
MSC2530818	TargetMol	Cat# TQ0266
IBMX (isobutylmethylxanthine)	TargetMol	Cat# T1713
Hydrocortisone (cortisol)	TargetMol	Cat# T1614
Dexamethasone	TargetMol	Cat# T1076
Triiodothyronine Sulfate	TargetMol	Cat# T29011
Agencourt RNA Clean XP beads	Beckman Coulter	Cat# A63987
Exonuclease I (ExoI)	New England Biolabs (NEB)	Cat# M0293S
KAPA HiFi PCR ReadyMix	Roche	Cat# KK2602
Agencourt AMPure XP beads	Beckman Coulter	Cat# A63880
RIPA Lysis Buffer (Strong)	Beyotime	Cat# P0013B
PVDF membrane	Millipore	Cat# HVLP00010
PK Mito Red (Mitochondrial probe)	GenVivo Tech	Cat# PKMR-1
ATP Standard	MCE	Cat# HY-B2176R
ADP Standard	MCE	Cat# HY-W010918R
Magnesium stearate	Macklin (Shanghai, China)	Cat# C12243643
Paraffin liquid	Macklin (Shanghai, China)	Cat# C12625589
Lanolin anhydrous	Macklin (Shanghai, China)	Cat# C15989714
Urea	Macklin (Shanghai, China)	Cat# C12407663
FR-1 (Powder)	This paper	WuXi AppTec
FCCP (Mitochondrial uncoupler)	TargetMol	Cat# T6834
BAM15 (Mitochondrial uncoupler)	TargetMol	Cat# T14497
Dimethyl sulfoxide (DMSO)	Merck	Cat# D2650-100ML
Glucose	Agilent Technologies	Cat# 103577-100
Sodium pyruvate	Agilent Technologies	Cat# 103578-100
Glutamine	Agilent Technologies	Cat# 103579-100
Seahorse XF DMEM	Agilent Technologies	Cat# 103575-100
Seahorse XF Calibrant	Agilent Technologies	Cat# 100840-000
Isoflurane (Anesthetic)	RWD Life Science	Cat# R510-22
Veet Depilatory Cream (Reckitt Benckiser)	Fisher Scientific	Cat# NC0786304
4% Paraformaldehyde (PFA) fixative	Beyotime	Cat# P0099-500mL
DAPI Staining Solution	Beyotime	Cat# C1005
Citrate Antigen Retrieval Solution	Servicebio	Cat# G1202

(*Continued on next page*)

REAGENT or RESOURCE	SOURCE	IDENTIFIER
Continued		
Hoechst 33342	Yeasen	Cat# 40730ES03
Sirius Red Staining Solution	Solarbio	Cat# G1473
Critical commercial assays		
DNA Clean & Concentrator-100 Kit	Zymo Research	Cat# D4029
Qubit^{TM} dsDNA HS Assay Kit	Thermo Fisher Scientific	Cat# Q33240
TruePrep DNA Library Prep Kit V2 for Illumina	Vazyme	Cat# TD502
EasyPure RNA Kit	TransGen Biotech	Cat# ER101-01
HiScript III All-in-one RT SuperMix Perfect for qPCR	Vazyme	Cat# R333-01
ChamQ SYBR qPCR Master Mix	Vazyme	Cat# Q321-03
pEASY-Uni Seamless Cloning and Assembly Kit	TransGen Biotech	Cat# CU101-02
BCA Protein Assay Kit	Thermo Fisher Scientific	Cat# 23227
BeyoECL Plus (Chemiluminescence kit)	Beyotime	Cat# P0018S
ADP-Glo^{TM} Kinase Assay	Promega	Cat# V9101
HTRF® Kinase Assay Reagents (KinEASE^{TM} or similar)	Revvity (Cisbio)	Cat# 62TK0PEB
Mitochondrial Membrane Potential Assay Kit with TMRE	Beyotime	Cat# C2001S
Seahorse XF Cell Mito Stress Test Kit	Agilent Technologies	Cat# 103015-100
Annexin V-FITC Apoptosis Detection Kit	Beyotime	Cat# C1062M
Senescence β-Galactosidase Staining Kit	Beyotime	Cat# C0602
Hematoxylin-Eosin (HE) Stain Kit	Solarbio	Cat# G1120
One Step TUNEL Apoptosis Assay Kit	Beyotime	Cat# C1086
Masson's Trichrome Stain Kit	Solarbio	Cat# G1340
DAB Chromogenic Kit	Servicebio	Cat# G1212
Deposited data		
DRUG-seq2 data	This paper	GEO: GSE303486
Bulk RNA-seq data	This paper	GEO: GSE303487
Single-crystal X-ray diffraction data collection of compound FR-1	The Cambridge Crystallographic Data Center (CCDC)	CCDC: 2498324
Experimental models: Cell lines		
Keloid fibroblasts isolated from the connective tissue of a Black, 35-year-old, female patient's skin with keloid.	ATCC	Cat# CRL-1762; RRID:CVCL_3730
Mouse 3T3-L1 preadipocyte embryonic fibroblast cell line derived from Swiss 3T3 mouse embryos	Shanghai Fuheng Biotechnology	Cat# FH0359; RRID:CVCL_0123
Experimental models: Organisms/strains		
Mouse: C57BL/6 (Male, 7 weeks old)	Shanghai SLAC Laboratory Animal Co., Ltd.	RRID:MGI:2159769
Mouse: BALB/c-Nude (Female, 10 weeks old)	Shanghai SLAC Laboratory Animal Co., Ltd.	RRID:IMSR_RJ:BALB-C-NUDE
Oligonucleotides		
Primers for quantitative PCR (qPCR)	PrimerBank	https://pga.mgh.harvard.edu/primerbank/; RRID:SCR6898
Recombinant DNA		
pFU-Tet-On-POU5F1 (Plasmid backbone)	Addgene	Plasmid #19778
pFU-Tet-On-GFP	This paper	N/A
pFU-Tet-On-UCP1	This paper	N/A

(*Continued on next page*)

Continued

REAGENT or RESOURCE	SOURCE	IDENTIFIER
pFU-Tet-On-UCP2	This paper	N/A
pFU-Tet-On-UCP3	This paper	N/A
Software and algorithms		
Drop-seq tools (Drop-seq analysis protocol)	Broad Institute	https://github.com/broadinstitute/Drop-seq; RRID:SCR_018142
STAR (Spliced Transcripts Alignment to a Reference)	Dobin et al.[76]	https://github.com/alexdobin/STAR; RRID:SCR_004463
Seurat R package	Satija Lab	https://satijalab.org/seurat/; RRID:SCR_022555
GSVA (Gene Set Variation Analysis)	Hänzelmann et al.[77]	https://bioconductor.org/packages/release/bioc/html/GSVA.html; RRID:SCR_021058
R Project for Statistical Computing	R Core Team	https://www.r-project.org/; RRID:SCR_001905
Subread (featureCounts)	Liao et al.[78]	http://subread.sourceforge.net/; RRID:SCR_012919
DESeq2	Love et al.[79]	https://bioconductor.org/packages/release/bioc/html/DESeq2.html; RRID:SCR_015687
ClusterProfiler	Wu et al.[80]	https://bioconductor.org/packages/release/bioc/html/clusterProfiler.html; RRID:016884
GO (Gene Ontology)	Ashburner et al.[81]	http://geneontology.org; RRID:002811
GSEA (Gene Set Enrichment Analysis)	Subramanian et al.[82]	http://www.gsea-msigdb.org/gsea/; RRID:003199
KEGG (Kyoto Encyclopedia of Genes and Genomes)	Kanehisa and Goto[83]	https://www.kegg.jp/; RRID:SCR_012773
PrimerBank (PCR primers database)	Spandidos et al.[84]	https://pga.mgh.harvard.edu/primerbank/; RRID:SCR_006898
FlowJo software	Tree Star (BD Biosciences)	https://www.flowjo.com/; RRID:SCR_008520
GraphPad Prism 9	GraphPad Software	https://www.graphpad.com/; RRID:SCR_002798
GraphPad Prism 10	GraphPad Software	https://www.graphpad.com/; RRID:SCR_002798
ImageJ (v1.54p)	National Institutes of Health (NIH)	https://imagej.net/; RRID:SCR_003070
Olympus Image Viewer software (OlyVIA)	Olympus (Evident)	https://www.olympus-lifescience.com/en/support/downloads/; RRID:SCR_024433
CrysAlisPro	Agilent Technologies Technologies	CrysAlisPro, Version 1.171.37.35
Olex2	O.V. Dolomanov et al.[85]	https://www.olexsys.org/olex2/
SHELXS	Sheldrick[86]	https://journals.iucr.org/a/issues/2008/01/00/sc5010/index.html; RRID:014220
SHELXL	Sheldrick[87]	https://journals.iucr.org/c/issues/2015/01/00/fa3356/index.html; RRID:014220
Other		
InfinityLab Poroshell 120 HILIC-Z (PEEK-lined, 2.1 × 100 mm, 2.7 μm)	Agilent Technologies	675775–924
Franz Diffusion Cell System	Hanson	RDS Phoenix DB-6
HPLC System	Agilent Technologies	1260
Agilent Seahorse XF Pro Analyzer	Agilent Technologies	Seahorse XF Pro

EXPERIMENTAL MODEL AND STUDY PARTICIPANT DETAILS

Patient samples

Adult human hypertrophic scar and keloid tissue samples were collected from patients undergoing surgical excision at Hospital for Skin Diseases (Institute of Dermatology), Chinese Academy of Medical Sciences and Peking Union Medical College, and the Department of Plastic Surgery, Peking University Third Hospital. The study was conducted in accordance with the Declaration of Helsinki and was approved by the Institute of Ethics Committee Review Board at Chinese Academy of Medical Sciences and Peking Union Medical College (Approval No. (2022) LKS-044) and the Medical Science Research Ethics Committee of Peking University Third Hospital (Approval No. (2025) YLS-585-01). Written informed consent was obtained from all donors prior to sample collection. The diagnosis was confirmed by pathological examination. All clinical donors recruited for this study were of Han Chinese ancestry. Primary fibroblasts isolated from these diverse patient samples were utilized for *in vitro* broad-spectrum anti-fibrotic testing (Figures 1F and 2C). Detailed demographic information of the donors, including age and gender, is provided in Table S2. For functional translational studies shown in Figure 5, keloid tissues from a 39-year-old male (chest) and an 81-year-old female (perineal) were utilized for the *ex vivo* explant and PDX models, respectively. In these models, harvested tissues from the donors were sectioned into uniform pieces and randomly allocated to experimental groups to minimize intra-lesional heterogeneity. The influence of gender on the results was not determined due to the limited sample size, but samples were randomly selected for downstream experiments. Detailed information regarding experimental design, grouping, and sample sizes for these studies is provided in the corresponding Figure Legends and method details sections.

Experimental animals

All animal experiments were conducted under the authorization of the Animal Care and Use Committee of Zhejiang University. The experimental protocols were approved by the Ethics Committee for Animal Use of Sir Run Run Shaw Hospital, School of Medicine, Zhejiang University (Approval No. SRRSH202302079) and the Zhejiang University Laboratory Animal Welfare and Ethics Review (Approval No. ZJU20250446). Experiments were performed in accordance with the Guide for the Care and Use of Laboratory Animals. Wild-type male C57BL/6 mice (7 weeks old) and immunodeficient female BALB/c nude mice (10 weeks old) were purchased from Shanghai SLAC Laboratory Animal Co., Ltd. (Shanghai, China). Mice were housed in a specific pathogen-free (SPF) facility under a 12-h light/12-h dark cycle at a controlled temperature and humidity, with *ad libitum* access to standard chow and water. Mice were acclimatized for at least 1 week before experiments. For the murine splinted excisional wound model and the PDX model, animals were randomly assigned to experimental groups; for the linear excisional wound model, animals were stratified based on scar size to ensure baseline consistency. Pre-established exclusion criteria included animal death or severe health issues unrelated to the experimental treatment; otherwise, all data were included in the analysis. Because sex was not a primary variable in the current study, the potential influence of sex-dependent biological differences represents a limitation of our *in vivo* models. Detailed information regarding experimental design, grouping, and sample sizes for these studies is provided in the corresponding Figure Legends and method details sections.

Cell lines

The commercial human keloid fibroblast line (isolated from a 35-year-old Black female) and the mouse 3T3-L1 cell line (derived from Swiss 3T3 mouse embryos) were utilized in this study. Both cell lines were authenticated via Short Tandem Repeat (STR) profiling. Furthermore, all cell lines were routinely tested for mycoplasma contamination and confirmed to be negative. The specific catalog numbers and source details for these cell lines are listed in the key resources table.

METHOD DETAILS

Isolation and culture of human keloid fibroblasts (HKFs)

Adult human keloid tissues were immersed in 70% ethanol for 1 min, washed with PBS supplemented with 2% penicillin-streptomycin, and finely minced. The tissue fragments were suspended in PBS and centrifuged at 1,500 rpm for 3 min. Enzymatic digestion was performed using a solution containing Dispase II (1 mg/mL), Collagenase II (1 mg/mL), DNase I (0.167 U/mL), 2% antibiotics, and 10 μM Y-27632 at 37°C for 3 h. Digestion was quenched by adding 10 mL of DMEM containing 10% FBS and 10 μM Y-27632. The suspension was stirred for a further 2 h, filtered through a 70 μm cell strainer, and centrifuged (1,500 rpm, 10 min). The resulting pellet was resuspended in KF medium (DMEM with 10% FBS, 2% penicillin-streptomycin, 1% ITS, and 20 ng/mL bFGF). Following red blood cell lysis on ice for 15 min, cells were washed, plated, and maintained at 37°C in 5% CO2 with medium changes every 48 h. Cells were subcultured using 0.25% trypsin-EDTA and cryopreserved in liquid nitrogen. Experiments were conducted using early-passage cells (≤P4) treated with specific small molecules for 48 h. Patient-derived keloid fibroblasts used throughout this study were restricted to early passages (≤P4). The sole exception was the use of late-passage cells ($p > 40$) as a positive control for true replicative senescence in the SA-β-gal assay.

Small molecule screen

HKFs were seeded at a density of 5,000 cells per well in a 96-well plate with KF medium. After cell attachment, bioactive agents were added to each well (compound arrangement is detailed in Table S7). Three independent biological replicates (three 96-well plates) were conducted to ensure reproducibility. After 48 h, cells were harvested for DRUG-seq2 library preparation.

DRUG-seq2 library preparation and data analysis

The library construction strictly followed the DRUG-seq2 protocol. Briefly, samples in 96-well plates were lysed, and reverse transcription mix was added. After RT-qPCR, products were pooled and purified using the DNA Clean & Concentrator-100 kit (Zymo Research) and Agencourt RNA Clean XP beads (Beckman Coulter). Exonuclease I was added to digest residual primers at 37°C for 30 min. Digested products were amplified using Kapa HiFi PCR ReadyMix and DRUG-seq2 pre-amplification primers. PCR products were purified, quantified via Qubit 4.0, and analyzed on an Agilent Technologies Fragment Analyzer 5400. The pre-library was processed by fragmentation with Tn5 and 3′-end enrichment using the TruePrep DNA Library Prep Kit V2 (Vazyme). The final library (250–450 bp) was selected using Agencourt AMPure XP beads, quantified, and sequenced on the Illumina HiSeq X Ten platform.

DRUG-seq2 data were processed to obtain an expression matrix according to the Drop-seq analysis protocol. Reads were split by barcodes and mapped to the human transcriptome (GRCh38) using STAR. Unique alignments were demultiplexed based on UMIs. Low-expression genes (log2 CPM <2) and samples with insufficient sequencing depth ($<2^{16}$ reads) were filtered. Dimensionality reduction was performed using the Seurat R package. Transcriptomic scoring methods (ssGSEA and Seurat's AddModuleScore) were applied to assess fibrosis-associated gene signatures from MSigDB and literature.

RNA extraction and RT-qPCR

Total RNA was extracted from cells using the EasyPure RNA Kit (TransGen). 1 μg of total RNA was reverse-transcribed to cDNA using HiScript III All-in-one RT SuperMix (Vazyme). qPCR was performed using ChamQ SYBR qPCR Master Mix (Vazyme) in a KUBO thermocycler system. Primers were obtained from PrimerBank. Experiments were performed in at least three independent biological replicates.

Western blotting

Cells were lysed in RIPA lysis buffer containing protease and phosphatase inhibitors. Lysates were centrifuged at 12,000 rpm for 15 min at 4°C. Protein concentration was determined using a BCA protein assay kit (Thermo Fisher Scientific). Proteins were separated by SDS-PAGE, transferred to PVDF membranes, and blocked with 3% BSA. Membranes were incubated overnight at 4°C with primary antibodies against COL1A1, MMP1, and GAPDH. After washing, membranes were incubated with HRP-conjugated secondary antibodies. Signals were detected using the BeyoECL Plus kit and an enhanced chemiluminescence (ECL) system. Western blotting was repeated at least three times independently.

Structural optimization and SAR strategy

To systematically elucidate the structure-activity relationship (SAR), a focused library of twelve derivatives (FR-1–FR-12) was designed and synthesized based on the lead scaffold. The structural optimization involved the strategic introduction of diverse functional groups, including heterocyclic and alkyl moieties, to probe the specific pharmacophoric requirements. These modifications aimed to explore the impact of steric and electronic variations on biological activity. The resulting compound library was subsequently subjected to biological evaluation.

Kinase activity and protein interaction assays

Kinase activity and protein interactions were assessed using ADP-Glo and HTRF assays, respectively. For the HTRF kinase assay, reagents were prepared with specific buffers containing MgCl2, MnCl2, SEB, and DTT. Compounds were transferred to 384-well plates using an Echo 655 acoustic dispenser. Kinase/metal ion solution was added and incubated, followed by the addition of substrate/ATP solution. Detection was performed using anti-phospho-substrate antibodies labeled with XL665 and Eu-Cryptate. Fluorescence signals at 620 nm and 665 nm were read on a microtiter plate reader. For the ADP-Glo kinase assay, reactions were performed in the presence of HEPES, MgCl2, Brij 35, EGTA, and DTT. ADP-Glo Reagent and Kinase Detection Reagent were added sequentially. Luminescence signals were recorded, and percent inhibition was calculated relative to controls.

Single-crystal X-ray diffraction analysis

Single-crystal X-ray diffraction data collection of compound FR-1 were measured on a ROD, Synergy Custom DW system, HyPix diffractometer equipped with a microfocus Cu Kα X-ray source (1.54184 Å) and a HyPix-6000HE area detector. The sample crystal was cooled to 100K using a cold nitrogen stream (Cobra by Oxford Cryosystems). Data reduction, cell refinement and experimental absorption correction were performed in CrysAlisPro. Using Olex2 program package, the structure was solved with the SHELXS structure solution program using Direct Methods and refined against F^2 with the SHELXL refinement package using full-matrix Least Squares minimisation. Multi-scan method was used for the absorption correction. All non-hydrogen atoms were refined anisotropically. Hydrogen atoms were generated geometrically at idealized position and constrained to ride on their parent. The crystallographic data for this paper were deposited in CCDC database with codes of 2498324.

Bulk RNA-seq data analysis

Bulk RNA-seq data were processed to obtain a gene count matrix using the featureCounts tool of the Subread software. Differential expression analysis was performed with DESeq2, and gene enrichment analysis (including GO and KEGG pathways) was conducted using clusterProfiler. Disease-associated gene sets for GSEA were sourced from DisGeNET.

Mitochondrial morphology analysis

Mitochondrial morphology was visualized using the PK Mito Red kit (GenVivo Tech) following the manufacturer's protocol. Briefly, the dye was diluted 1:1000 in pre-warmed culture medium and added to cells for 15 min at 37°C. After washing twice with fresh medium, images were acquired using Confocal Laser Scanning Microscopy (CLSM).

Measurement of mitochondrial membrane potential

Mitochondrial membrane potential was assessed using the TMRE Assay Kit (Beyotime) according to the manufacturer's instructions. A working solution was prepared by diluting TMRE (1000×) and DAPI (1000×) in the provided detection buffer. Cells were incubated with 1 mL of the working solution for 30 min at 37°C. Following a wash step, fluorescence images were acquired using a fluorescence microscope.

ATP/ADP mass spectrometry detection

Cellular ATP and ADP levels were measured using liquid chromatography-tandem mass spectrometry (LC-MS/MS). Cells were extracted with 80% methanol, vortexed, sonicated, and centrifuged. The supernatant was analyzed using an Agilent Technologies 1260-Ultivo UPLC-MS/MS system equipped with an InfinityLab Poroshell 120 HILIC-Z (PEEK-lined) column. Mobile phases consisted of water and acetonitrile with a gradient elution. ATP was detected using the transition 508.1 > 136.1 m/z, and ADP was detected using 428.1 > 136.1 m/z in positive ion mode.

Measurement of mitochondrial respiration

Mitochondrial respiration was assessed using the Seahorse XF Cell Mito Stress Test Kit (Agilent Technologies) on a Seahorse XFe96 Extracellular Flux Analyzer. Briefly, cells were pre-treated with vehicle (DMSO) or 3 μM FR-1 for 48 h. Following treatment, cells were harvested, seeded into XF96 cell culture microplates at a density of 15,000 cells/well, and allowed to adhere overnight. On the day of the assay, the culture medium was replaced with unbuffered XF assay medium (pH 7.4) supplemented with 10 mM glucose, 1 mM pyruvate, and 2 mM glutamine. The cells were equilibrated in a non-CO2 incubator at 37°C for 1 h. The oxygen consumption rate (OCR) was monitored following sequential injections of oligomycin (1.5 μM), FCCP (1.5 μM), and rotenone/antimycin A (0.5 μM). Post-assay, OCR data were normalized to total protein concentration determined by the BCA assay.

Flow cytometry detection of cell apoptosis

Cells were harvested and washed with PBS. Apoptosis was detected using the Annexin V-FITC Apoptosis Detection Kit (Beyotime). Cells were resuspended in binding buffer and stained with Annexin V-FITC and propidium iodide (PI) for 10–20 min in the dark at room temperature. Analysis was performed within 1 h using a CytoFLEX LX flow cytometer. Data were analyzed using FlowJo software.

Cell senescence β-galactosidase staining

Cells were fixed with 4% paraformaldehyde for 15 min and incubated overnight at 37°C in the dark with staining working solution containing X-gal (Beyotime). Senescent cells (appearing blue) were observed and counted under an optical microscope.

Preparation of FR-1 formulations

For *in vivo* administration, the FR-1 injection solution was prepared using a co-solvent vehicle system consisting of 10% DMSO, 40% PEG300, 5% Tween 80, and 50% saline. Briefly, the FR-1 powder was first completely dissolved in DMSO, followed by the sequential addition of PEG300, Tween 80, and saline with vigorous vortexing to ensure a clear, homogeneous solution. The final formulation was freshly prepared prior to use and sterilized by filtration through a 0.22 μm membrane. For the topical ointment, aqueous and oily phases were prepared separately. The oily phase consisted of magnesium stearate (8.5 g), paraffin liquid (34 g), and lanolin anhydrous (14 g) melted at 70°C. The aqueous phase consisted of distilled water (32 mL) and urea (10 g). FR-1 (0.197 g) was dissolved in the oily phase. The phases were mixed and stirred until cool to yield an ointment with 0.2% (w/w) FR-1.

Transdermal permeability and HPLC analysis

Transdermal permeability was evaluated using a porcine skin model in a diffusion cell system. The donor chamber contained 0.2% FR-1 ointment, and the receptor chamber contained saline. After 48 h at 32°C, retained FR-1 in the skin was extracted with methanol via ultrasonication. FR-1 quantification was performed by HPLC using a Thermo Fisher Scientific U3000 system with a C18 column (150 mm × 4.6 mm, 5 μm). The mobile phase was a methanol/water gradient at 1.0 mL/min, with detection at 230 nm.

Linear excisional wound model

Seven-week-old male C57BL/6 mice were anesthetized with 2% isoflurane. After hair removal and disinfection, two rectangular full-thickness skin wounds (1.5 cm × 0.2 cm) were excised on the dorsum, exposing the underlying muscle. On day 12 post-surgery (D-1), scars were macroscopically examined. Sample size (*n* = 10 scars from 5 mice per group) was determined based on previous studies and preliminary experiments to ensure adequate statistical power. To minimize baseline heterogeneity, mice were stratified based on scar area and then assigned to experimental groups. Starting from D0 (day 13), mice received topical ointment or injections according to the experimental design. On the experimental endpoint, mice were euthanized. Scars were harvested for RNA/protein extraction (one side) and histopathological analysis (the other side).

Murine splinted excisional wound model

Mice were prepared as described above. Two full-thickness wounds (8 mm diameter) were created on the dorsum. Silicone rings were sutured around the wounds to prevent contraction. Mice were randomly assigned to the FR-1 treatment or vehicle control group. 0.1 mL of FR-1 (0.1 mM) or vehicle was injected at the wound base (3–5 mice per group). Wounds were dressed daily. On day 60 post-surgery, mice were euthanized, and tissues were harvested.

Ex vivo keloid explant culture and treatment

Primary keloid tissues, obtained from a single individual donor, were washed twice in phosphate-buffered saline (PBS) supplemented with 1% penicillin-streptomycin, followed by the careful microdissection of the epidermis and subcutaneous fat. The isolated dermal tissues were minced randomly distributed into approximately 1 mm^3 explants and evenly seeded into T25 culture flasks. To facilitate tissue attachment, the explants were cultured in Dulbecco's Modified Eagle Medium (DMEM) using a step-down serum protocol: 1 mL of DMEM with 40% fetal bovine serum (FBS) on Day 1, 2 mL with 20% FBS on Day 3, and 4 mL with 15% FBS on Day 5. On Day 6 (designated as treatment Day 0), the corresponding flasks were randomly assigned to the three experimental groups and treated with vehicle (DMSO), 1 μM FR-1, or 1 μM triamcinolone acetonide (TA). Cultures were maintained at 37°C in a normoxic incubator for 7 days, with culture media and respective treatments replenished every 48 h. To monitor fibroblast outgrowth, fixed-position images were captured on treatment Days 0, 2, 4, and 7. Following the 7-day treatment period, the explants were harvested, sectioned, and processed for hematoxylin and eosin (H&E), Masson's trichrome, and immunohistochemical (IHC) staining.

Patient-derived xenograft (PDX) model

Fresh human keloid tissues, obtained from a single individual donor, were cut into uniform blocks (approximately 0.5 cm^3, 130–150 mg) and implanted subcutaneously into the backs of BALB/c nude mice (female, 10 weeks old). Grafts were allowed to stabilize for 16 days. One mouse exhibiting premature graft atrophy prior to treatment was excluded. On day 17, the remaining mice were randomly allocated into two experimental groups (3 mice per group) with matched baseline graft volumes: the FR-1 treatment group (0.1 mM) and the Vehicle control group. Treatments (0.1 mL) were administered via intralesional injection every 15 days. All grafts were harvested on day 60 for subsequent histological and molecular analyses.

Standardized photographic protocol

To ensure consistency and reproducibility, a standardized photographic protocol was strictly adhered to throughout the study. All macroscopic wound images were acquired using an Apple iPhone 15 Pro (Cupertino, CA, USA), utilizing the main camera system (24 mm equivalent focal length, f/1.78 aperture, 48 MP sensor) in standard photo mode without digital filters or post-processing enhancements. Anesthetized mice were positioned prone on a flat, non-reflective surface, and photographs were taken from a perpendicular angle (90° to the wound plane) at a consistent working distance range of approximately 10–15 cm to ensure the wound area filled the central frame while minimizing perspective distortion. Crucially, to account for minor variations in the working distance, a standardized metric ruler was placed within the frame of every photograph, adjacent to the wound and at the same focal plane. Prior to any morphometric measurement, the scale of each individual image was calibrated using this internal ruler (pixels/mm) in ImageJ, ensuring that the physical distance of the camera did not introduce measurement bias. For image presentation, linear brightness adjustments were applied uniformly to the entire image solely to ensure visual consistency and clarity. These global adjustments did not alter data integrity, and all quantitative morphometric analyses were strictly performed on the original, unprocessed raw images.

Histopathology, Immunohistochemistry (IHC), Immunofluorescence (IF), and Terminal deoxynucleotidyl transferase dUTP nick end labeling (TUNEL) assay.

For *in vivo* tissue analysis, tissue samples were fixed in 4% PFA, embedded in paraffin, and sectioned. **Histological staining:** Sections were stained with Hematoxylin and Eosin (H&E) or Masson's Trichrome using kits from Solarbio. Sirius Red staining was performed using Weigert's iron hematoxylin and Sirius Red solution (Solarbio), followed by observation under polarized light microscopy. **Immunohistochemistry:** Sections underwent antigen retrieval with citrate buffer (Servicebio) and endogenous peroxidase blocking. After blocking with goat serum, sections were incubated overnight with primary antibodies (Rabbit anti-α-SMA, anti-TGF-β1), followed by incubation with appropriate biotin-conjugated secondary antibodies. Signals were developed using a DAB Chromogenic Kit (Servicebio). **Immunofluorescence:** For *in vitro studies,* sections were permeabilized with 0.2% Triton X-100 and blocked with 3% BSA. Sections were incubated with primary antibodies (Rabbit anti-α-SMA, anti-β-catenin) overnight, followed by fluorescently labeled secondary antibodies (CoraLite488/594-conjugated). Nuclei were counterstained with DAPI. **TUNEL**

staining: Apoptosis in paraffin-embedded tissue sections was assessed using the One Step TUNEL Apoptosis Assay Kit (Beyotime) following the manufacturer's protocol. Briefly, tissue sections were deparaffinized in xylene and rehydrated through a graded ethanol series. To permeabilize the tissues, sections were incubated with Proteinase K (20 μg/mL) at 37°C for 20 min. After washing with PBS, the sections were incubated with the TUNEL reaction mixture containing terminal deoxynucleotidyl transferase (TdT) and fluorescein-dUTP at 37°C for 60 min in a humidified chamber in the dark. DNase I-treated sections served as positive controls. Nuclei were counterstained with DAPI. All slides were scanned using an Olympus VS120/VS200 scanner and analyzed using ImageJ software.

Plasmid construction and transfection

For the construction of pFU-Tet-On-GFP, pFU-Tet-On-UCP1, pFU-Tet-On-UCP2, and pFU-Tet-On-UCP3 plasmids, full-length cDNAs encoding GFP, UCP1, UCP2, and UCP3 were amplified by PCR and subcloned into the pFU-Tet-On-POU5F1 backbone using the pEASY-Uni Seamless Cloning and Assembly Kit (TransGen). TransStbl3 Chemically Competent Cells were used for plasmid amplification.

In vitro permeation testing (IVPT) and quantification

IVPT was conducted on excised full-thickness porcine skin using Franz diffusion cells (diffusion area: 1.77 cm^2). A 1.0% (w/w) FR-1 ointment was applied to the donor chamber, while the receptor chamber contained physiological saline with 20% ethanol maintained at 32°C under constant stirring. After 24 h, drug content in separated skin layers (stratum corneum, epidermis, and dermis) and receptor fluid was extracted with methanol and quantified by HPLC. Analysis was performed using a Phenomenex ACE EXCEL SUPER C18 column (4.6 × 150 mm, 5 μm) maintained at 40°C. The mobile phase consisted of 0.1% trifluoroacetic acid (TFA) in water (Solvent A) and acetonitrile (Solvent B) delivered at 1.0 mL/min. A gradient elution was applied: 0–15 min (5%–95% B), 15–20 min (95% B), and 20–25 min (re-equilibration to 5% B). Detection was monitored at 230 nm.

Establishment of MI mouse model of myocardial infarction

Myocardial infarction (MI) was induced in 8-week-old mice by permanent ligation of the left anterior descending artery (LAD). Briefly, mice were anesthetized with isoflurane (2.5% induction, 1.5% maintenance in 97.5–98.5% O2), intubated with a 24 G catheter, and mechanically ventilated (VentElite, Harvard Apparatus; stroke volume: 220 μL; rate: 120 breaths/min). The LAD was ligated using a 6-0 nylon suture. Successful occlusion was confirmed by immediate pallor of the left ventricular anterior wall.

Isolation and culture of primary cardiac fibroblasts

Primary cardiac fibroblasts were isolated from the left ventricular (LV) tissue of wild-type mice post-MI. Briefly, LV tissue was dissected, washed in cold PBS, and minced into fragments (<1 mm3). Digestion was performed using a solution containing 1 mg/mL Collagenase I (Gibco, 17100-017) and 1 mg/mL Dispase II (Sigma, D4693-1G) at 37°C for 1.2–2.0 h. The digest was centrifuged at 500 × g for 5 min, resuspended in red blood cell (RBC) lysis buffer, and filtered through a 70-μm cell strainer. Following a second centrifugation (450 × g, 5 min), cells were resuspended in fibroblast culture medium (High Glucose DMEM supplemented with 20% FBS, 1% NEAA, 1% GlutaMAX, and 1% penicillin/streptomycin) and plated in 24-well plates for adherence and subsequent experiments.

QUANTIFICATION AND STATISTICAL ANALYSIS

Statistical analyses were performed using GraphPad Prism 9.0/10.0 software (GraphPad Software, San Diego, CA, USA). Bioinformatic analyses (DRUG-seq2 and Bulk RNA-seq) were performed using R software packages as described in the specific method sections. Data are presented as the mean ± standard error of the mean (SEM) for *in vivo* experiments and mean ± standard deviation (SD) for *in vitro* experiments. The exact value of n represents the number of independent biological replicates (e.g., number of mice, number of scars, independent cell culture experiments, or high-power fields [HPFs]), unless otherwise specified as technical replicates, and is detailed in the respective figure legends.

Comparisons between two groups were analyzed using two-tailed unpaired Student's t-tests. Comparisons among multiple groups were performed using one-way or two-way analysis of variance (ANOVA) followed by Tukey's or Dunnett's post hoc multiple comparison tests. To verify the assumptions of the statistical approach, the normality of data distribution was assessed using the Shapiro-Wilk test, and homogeneity of variance was confirmed using the Brown-Forsythe test. Statistical significance was defined as $p < 0.05$.

Strategies for randomization and stratification were determined by the experimental design requirements. As detailed in the Experimental Models section, animals in the linear excisional wound model were stratified based on initial scar size to minimize baseline heterogeneity, while animals in the splinted excisional wound model were randomly assigned to experimental groups. Sample sizes were estimated based on previous studies and preliminary experiments to ensure adequate statistical power. Data exclusion criteria were pre-established to exclude only samples from animals that died or exhibited severe health issues unrelated to the experimental treatment; otherwise, all data were included in the analysis. To minimize subjective bias, histological quantification and image analysis were performed using coded samples where the investigator was blinded to the specific treatment allocation.

Cell Reports Medicine, Volume 7

Supplemental information

Transcriptome-guided development of a fibrosis-reversal compound reduces skin scarring and allows regeneration via mitochondrial uncoupling

Chun-Ye Chen, Ruilin Xu, Mingguang Mo, Jiahao Wu, Jun Chi, Zhang-Rui Wu, Yi Wang, Xin-Cao Zhong, Xiao-Ying Lin, Yang Liu, Jingdong Wu, Huaan Fang, Hongli Jia, Hongsen Bi, Yong Yang, Wei-Qiang Tan, and Yang Zhao

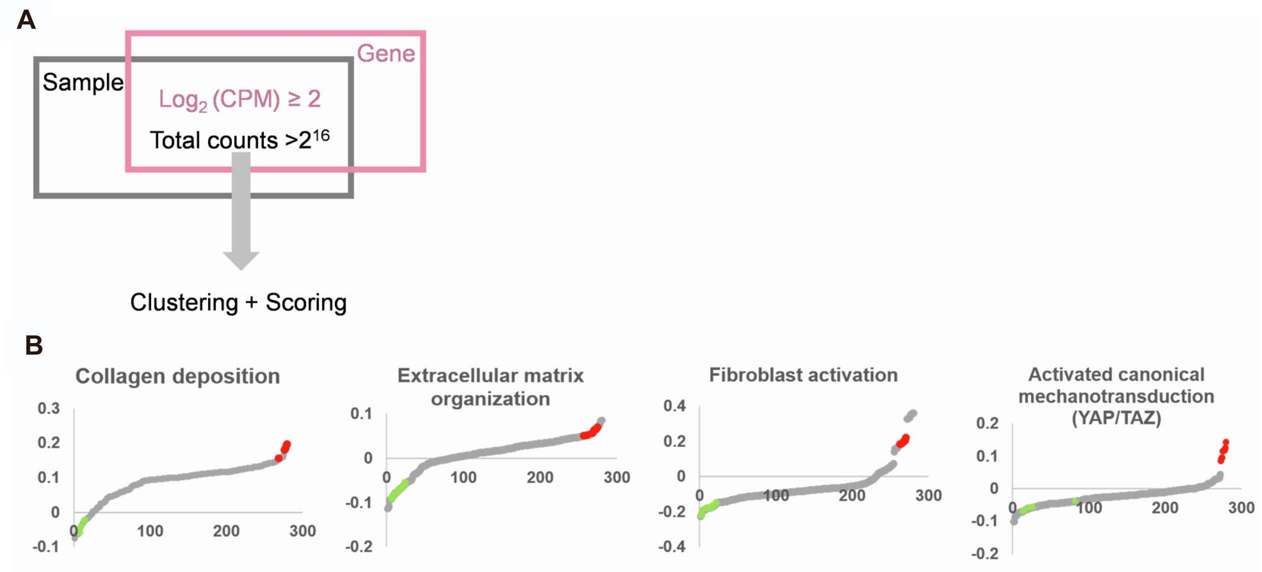

Compound	Rank
Sorafenib	1
L-Ascorbic acid	2
ICG-001	3
Rottlerin	4
bFGF	5

Compound	Rank
Sorafenib	1
L-Ascorbic acid	2
Rottlerin	3
ICG-001	4
SB431542	5

Compound	Rank
Sorafenib	1
Rottlerin	2
L-Ascorbic acid	3
SB431542	4
ICG-001	5

Compound	Rank
Sorafenib	1
SB431542	2
Nintedanib	3
Rottlerin	4
SAR-100842	5

Figure S1. DRUG-seq2 transcriptomic data preprocessing and gene set scoring, related to Figure 1. (A) Specific plan for DRUG-seq2 data preprocessing. (B) Module scores for four fibrosis-associated gene sets were calculated using “addModuleScore” function in Seurat (R package). Dots denote treatment groups: red, TGF-β1; green, Rottlerin. Top five candidates per set identified based on scores.

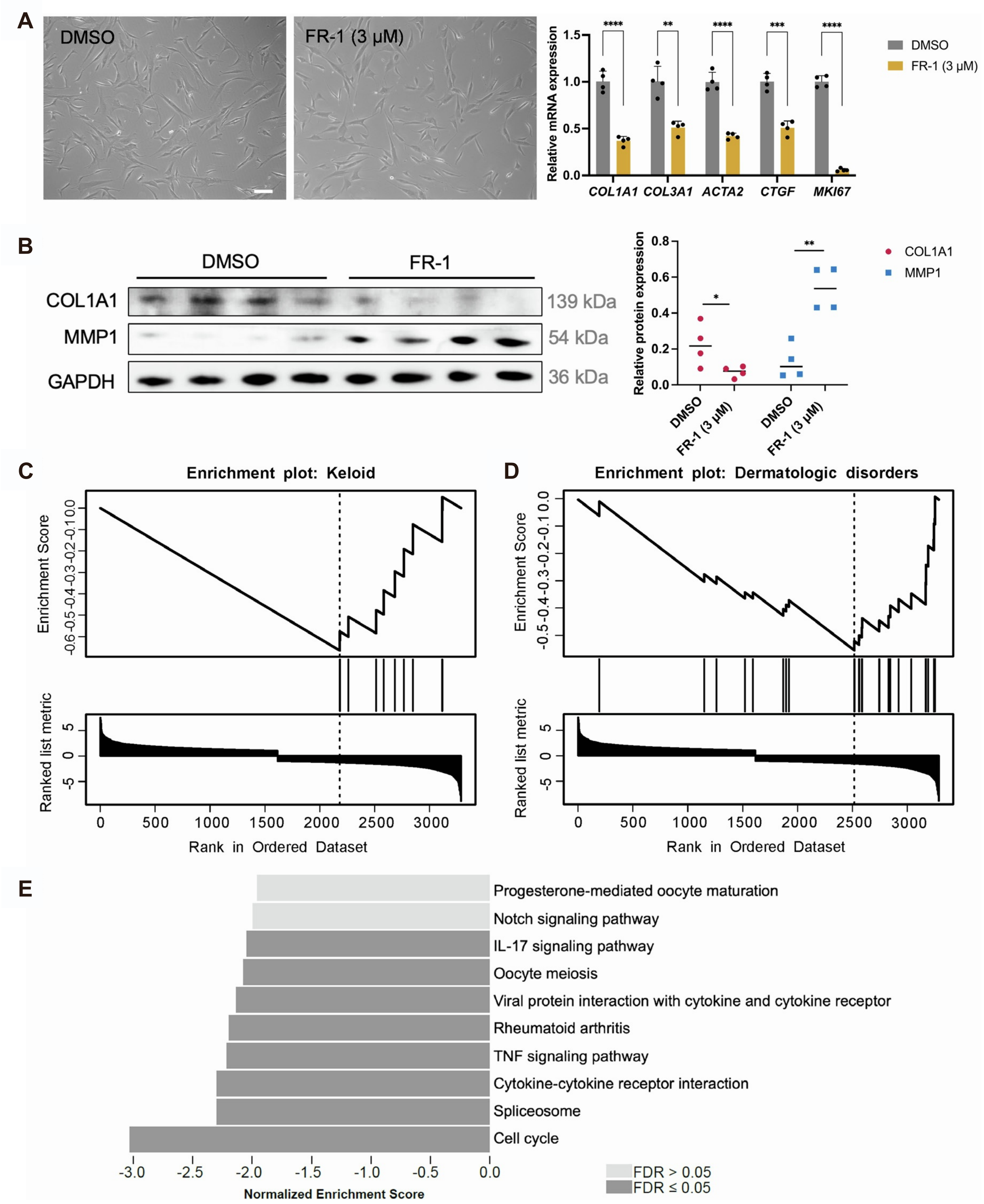

Figure S2. FR-1 reverses fibrotic phenotypes in African-descent keloid fibroblasts and drives superior transcriptomic regulation relative to Rottlerin, related to Figure 2. (A) Representative bright-field images (scale bar: 100 μm) and RT-qPCR analysis of fibrosis-related genes (*COL1A1, COL3A1, ACTA2, CTGF*) and the proliferation marker *MKI67* in ATCC CRL-1762 cells (Black donor) treated with DMSO or 3 μM FR-1. (B) Western blot analysis of COL1A1 and MMP1 protein levels in cells treated as in (A). (C, D) GSEA plots depicting significant downregulation of DisGeNET gene sets (keloid, dermatologic disorders) in FR-1 compared to Rottlerin. Vertical lines represent gene positions in the ranked list; the black curve indicates the enrichment score (ES) trajectory. (E) Top 10 significantly downregulated KEGG pathways in FR-1 versus Rottlerin, ranked by normalized enrichment score. Gray bars denote downregulated pathways; color intensity corresponds to FDR significance. Data are mean ± SD (n=4), n represents the number of independent biological replicates. $*P < 0.05$; $**P < 0.01$; $***P < 0.001$; $****P < 0.0001$ vs. DMSO by Student's t test.

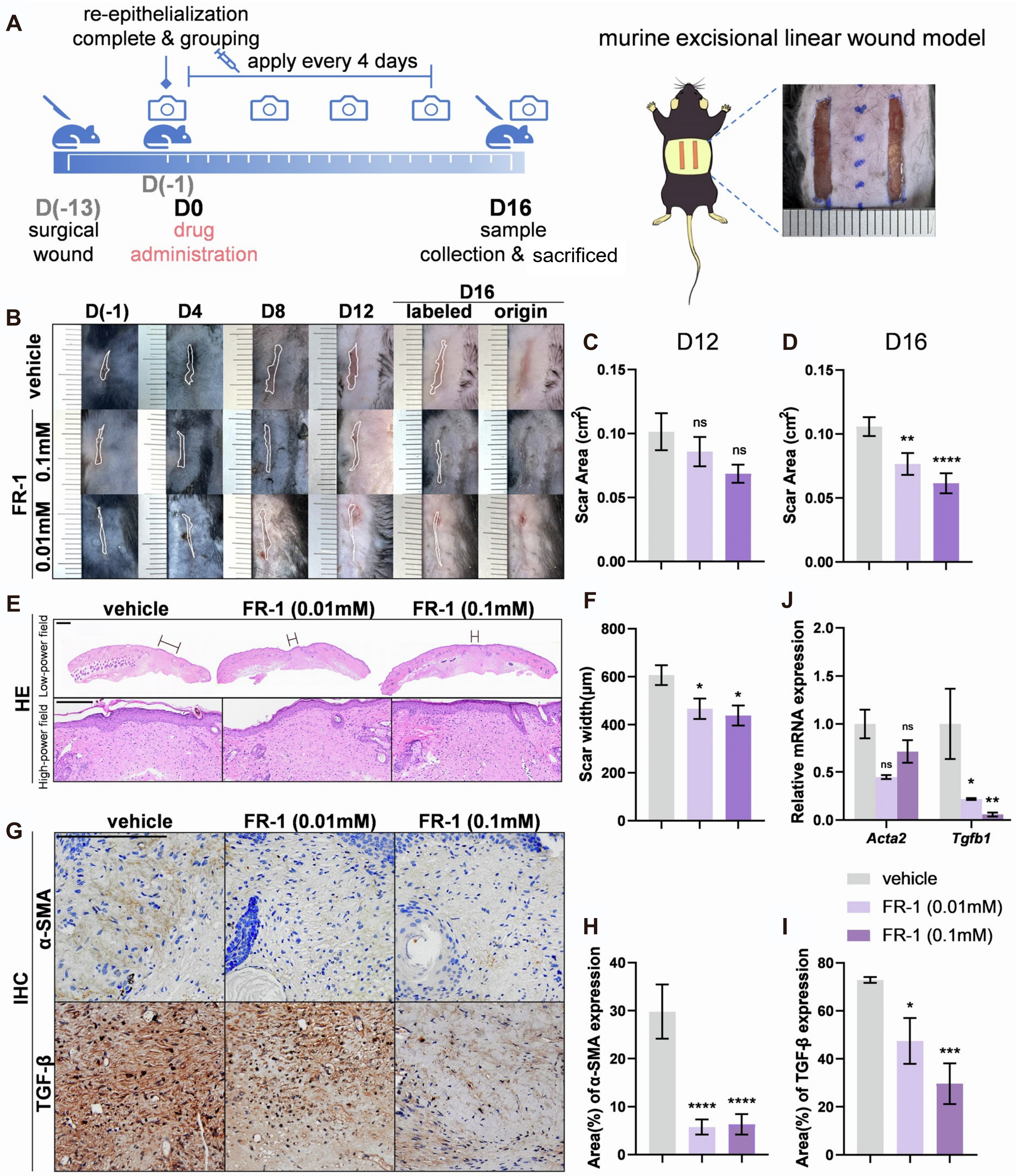

Figure S3. FR-1 injection reduces scar formation in a murine linear excisional wound model, related to Figure 3. (A) Schematic and timeline of the murine linear excisional wound model (1.5 cm × 0.2 cm wounds, 0.4 cm lateral to midline). (B) Representative images of scars in mice treated with vehicle, 0.1 mM FR-1, or 0.01 mM FR-1 at indicated time points (white boxes: scar areas), and quantification of scar area on (C) D12 and (D) D16 (n = 10 scars from 5 mice per group). (E) H&E-stained scar tissues on D16 (black line: scar width; scale bar: 500 μm, high-magnification: 200 μm), and (F) scar width quantification (n=5 mice). (G) Immunohistochemistry for α-SMA and TGF-β1 in D16 scar tissues (scale bar: 200 μm), and quantification of (H) α-SMA and (I) TGF-β1 expression (n = 5 HPFs from 2 mice). (J) RT-qPCR analysis of fibrotic marker mRNA expression (*Acta2, Tgfb1*) in D16 scars (n = 3 mice). Data are mean ± SEM, n represents the number of independent biological replicates. $*P < 0.05$; $**P < 0.01$; $***P < 0.001$; $****P < 0.0001$; ns, not statistically significant vs. vehicle by one-way ANOVA.

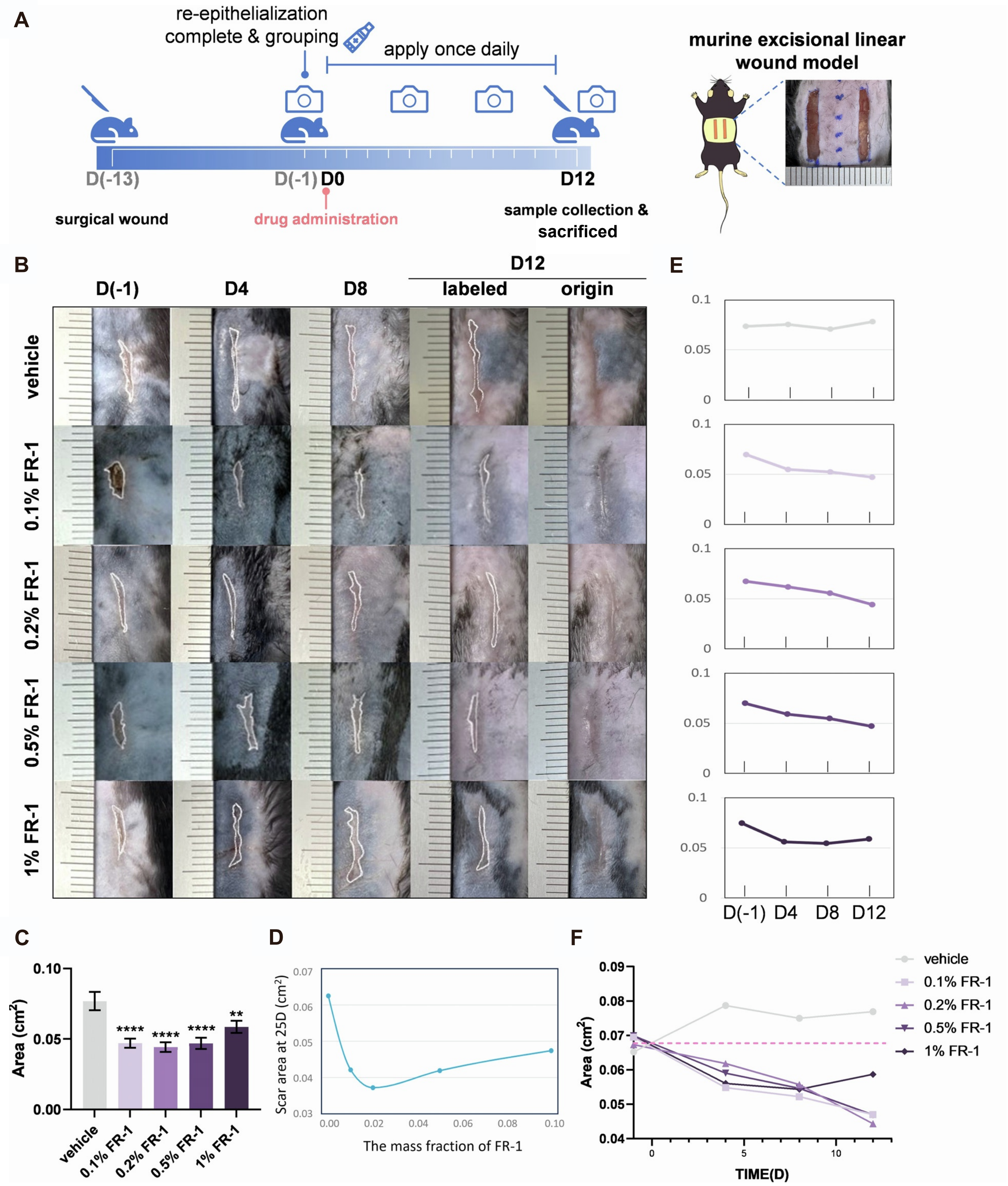

Figure S4. Dose-dependent therapeutic efficacy of FR-1 ointment on scar formation in a murine linear excisional wound model, related to Figure 3. (A) Schematic and timeline of the murine linear excisional wound model (1.5 cm × 0.2 cm wounds, 0.4 cm lateral to midline). (B) Representative images of scar formation in mice treated with vehicle, 0.1%, 0.2%, 0.5% and 1% FR-1 ointment at indicated time points (white boxes: scar areas; n=10 scars from 5 mice per group). (C) Bar graph and (D) line graph showing quantitative data of scar area on D12. (E) Individual-group temporal profiles of scar area. (F) Combined line graph showing temporal changes in scar area for all groups. Data are mean ± SEM, n represents the number of independent biological replicates. $**P < 0.01$; $****P < 0.0001$ vs. vehicle by one-way ANOVA.

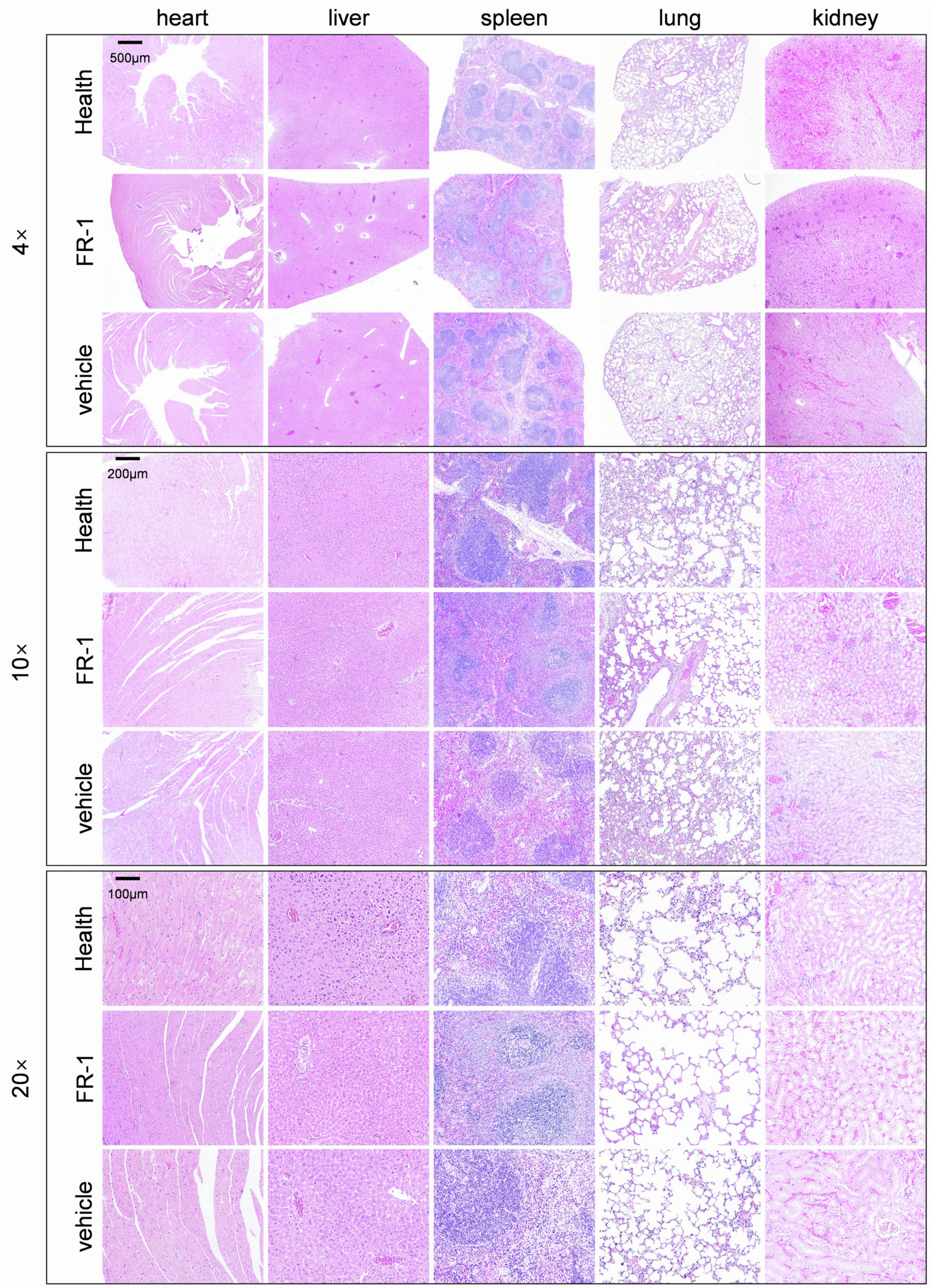

Figure S5. Histopathological analysis of major organs from the murine linear excisional wound model, related to Figure 3. Representative H&E staining images of the heart, liver, spleen, lung, and kidney from healthy mice, topical FR-1-treated mice, and vehicle-treated mice at the experimental endpoint.

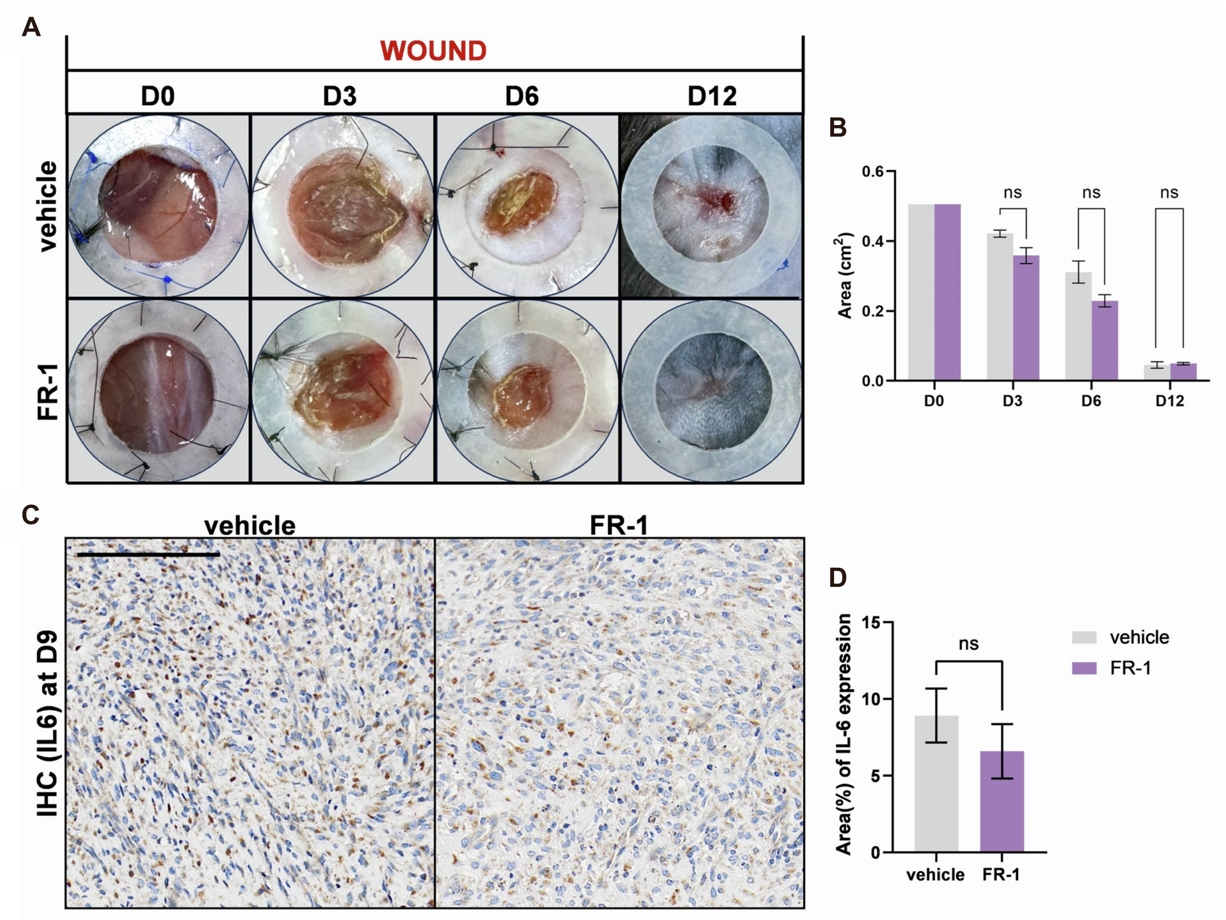

Figure S6. FR-1 did not delay acute wound closure, related to Figure 4. (A) Representative images of wound healing progression (D0-D12) with vehicle or FR-1 treatment. (B) Quantification of wound areas over time (n = 8–10 scars from 4–5 mice per group). (C) Immunohistochemistry for IL-6 in D9 wound tissues (scale bar: 200 μm), and quantification of (D) IL-6 expression (n = 4 HPFs from 2 mice). Data are represented as mean ± SEM, n represents the number of independent biological replicates, ns, not statistically significant by Student's t test.

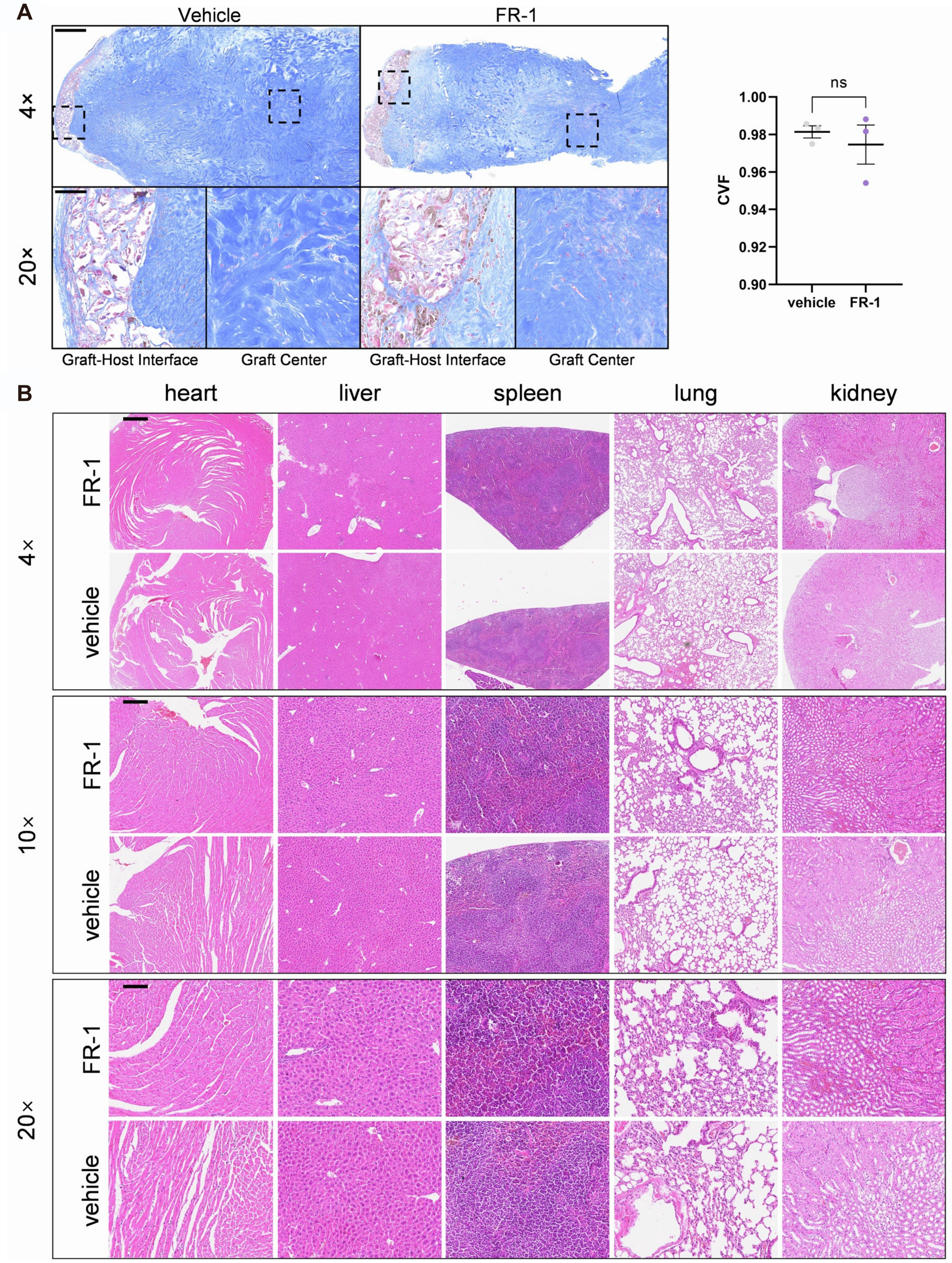

Figure S7. Histopathological analysis of the grafts and major organs from the human keloid patient-derived xenograft (PDX) model, related to Figure 5. (A) Representative Masson's trichrome staining images and corresponding quantification of collagen volume fraction (CVF) of the viable keloid grafts from the vehicle and FR-1 treatment groups at the experimental endpoint (n = 3). Scale bars: 500 μm (4×) and 100 μm (20×). (B) Representative H&E staining images of the heart, liver, spleen, lung, and kidney from healthy mice, topical FR-1-treated mice, and vehicle-treated mice at the experimental endpoint. Scale bars: 500 μm (4×), 100 μm (10×), and 100 μm (20×). Data are represented as mean ± SEM, n represents the number of independent biological replicates, ns, not statistically significant by Student's t test.

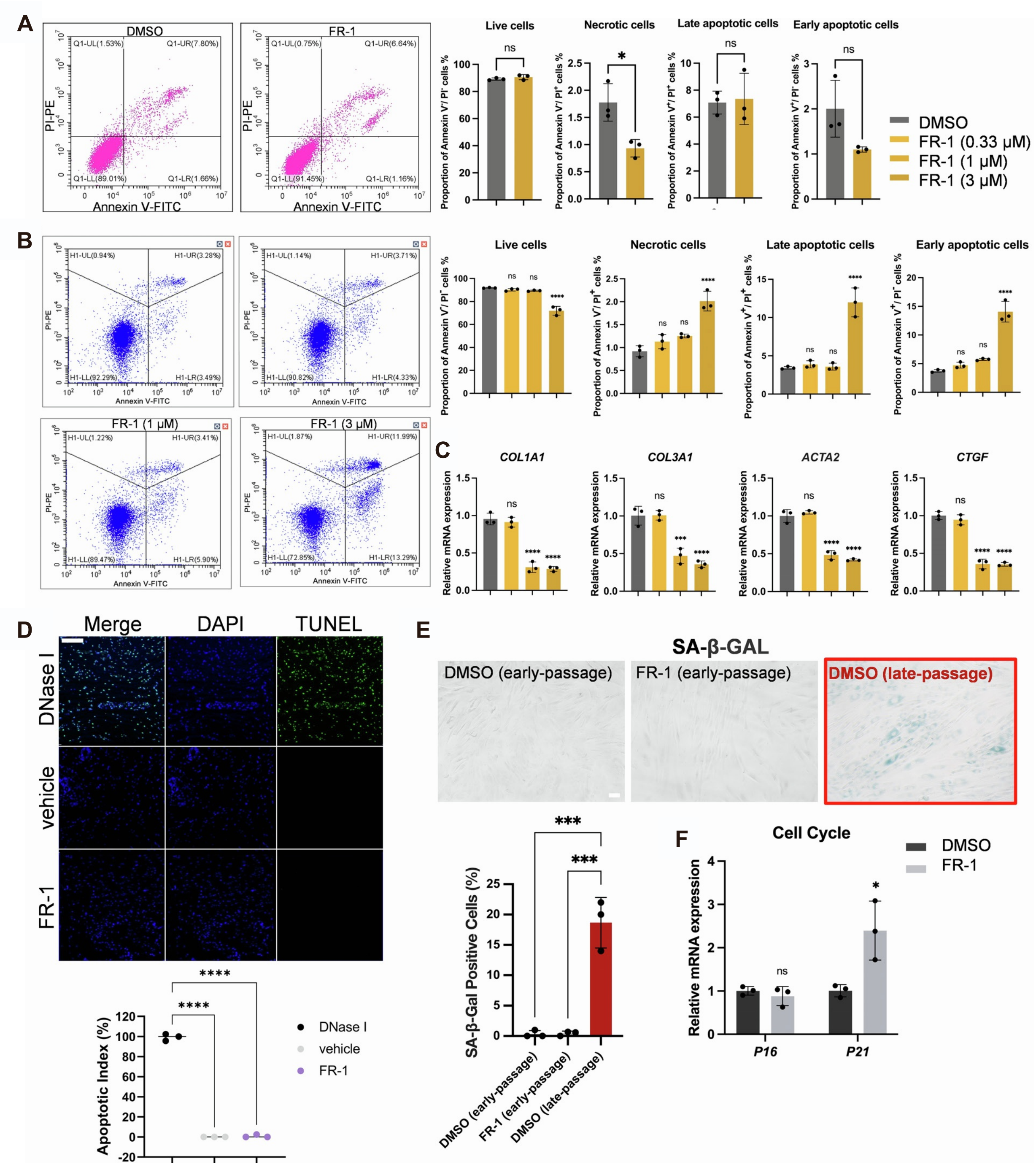

Figure S8. FR-1 reverses fibrosis independent of apoptosis or cellular senescence, related to Figure 6. (A and B) Flow cytometric analysis of apoptosis (Annexin V/PI staining) in keloid fibroblasts treated with DMSO or FR-1 at (A) 3 μM for 48 hours and (B) the indicated concentrations (0.33, 1, and 3 μM) for 120 hours. Representative plots (left) and quantification of apoptotic rates (right) are shown. (C) RT-qPCR quantification of fibrosis-related genes (*COL1A1*, *COL3A1, ACTA2, CTGF*) following 5-day FR-1 treatment. (D) Representative TUNEL staining of scar tissues harvested from the murine linear excision model at the experimental endpoint, following treatment with FR-1 ointment or vehicle. DNase I-treated sections served as a positive control. Scale bar: 100 μm. The apoptotic index is quantified below the representative images. (E) Representative images and quantification of senescence-associated β-galactosidase (SA-β-Gal) staining. Scale bar: 200 μm. (F) RT-qPCR of cell senescence markers (*P16, P21*) in FR-1 and DMSO-treated cells. Data are mean ± SD (n=3), n represents the number of independent biological replicates. ∗P < 0.05; ∗∗*P* < 0.01; ∗∗∗*P* < 0.001; ∗∗∗∗*P* < 0.0001; ns, not statistically significant vs. DMSO by (A, F) Student's t test and (B-E) one-way ANOVA.

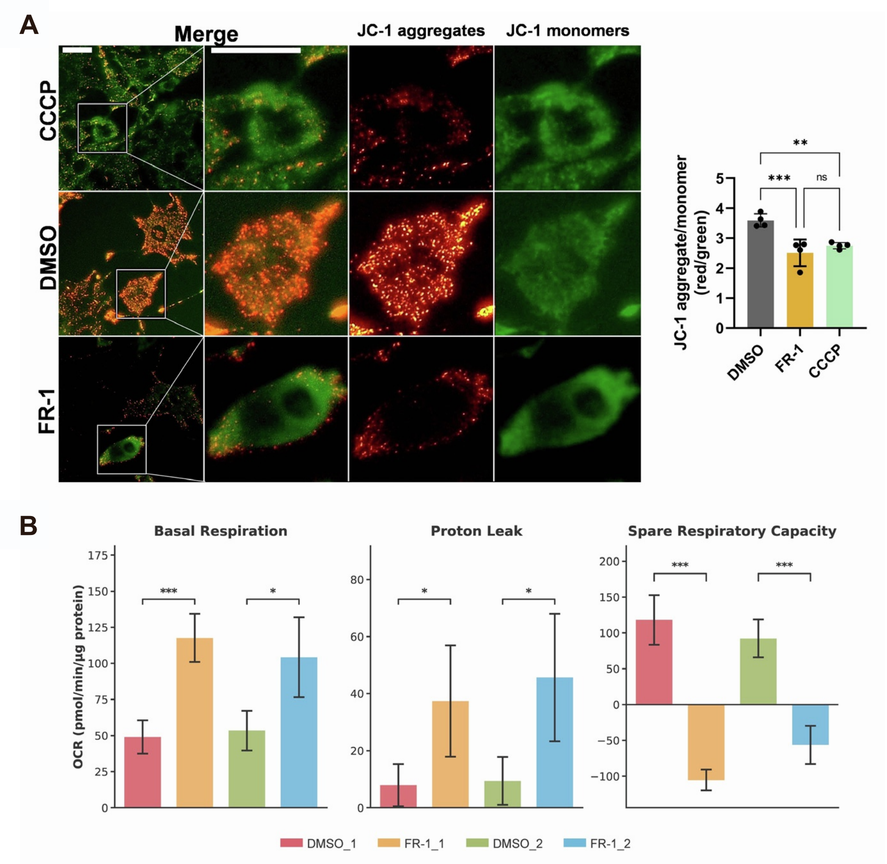

Figure S9. Validation of FR-1 uncoupling activity, related to Figure 6. (A) Representative JC-1 fluorescence images (left) and quantitative analysis of the red/green fluorescence intensity ratio (right) in 3T3-L1 fibroblasts treated with DMSO, FR-1 (1.5 μM), or CCCP (positive control) (n=4). A decrease in the red/green ratio indicates mitochondrial depolarization. Scale bar: 50 μm.
(B) Quantification of proton leak derived from the Seahorse OCR analysis in FR-1- or DMSO-treated fibroblasts (n=4). Data are mean ± SD, n represents the number of independent biological replicates. $**P < 0.01$; $***P < 0.001$; ns, not statistically significant vs. DMSO by one-way ANOVA.

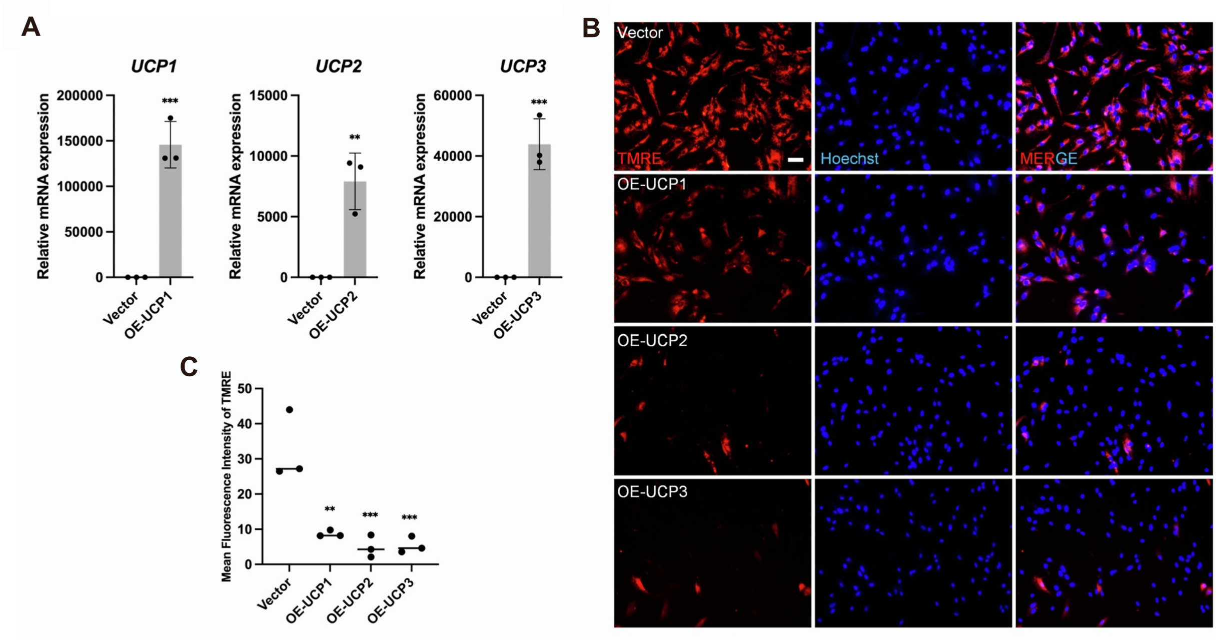

Figure S10. Validation of inducible UCP overexpression and functional mitochondrial depolarization, related to Figure 6. (A) Analysis of *UCP*s overexpression at the mRNA level. (B) Representative image of mitochondrial membrane potential (ΔΨm) changes following UCP overexpression compared to vector control. Scale bar: 100 μm. (C) Quantification of ΔΨm (measured by TMRE) in UCP-overexpressing versus vector control groups. Data are mean ± SD (n=3), n represents the number of independent biological replicates. $*P < 0.05$; $**P < 0.01$; $***P < 0.001$; $****P < 0.000$; ns, not statistically significant vs. vector by one-way ANOVA.

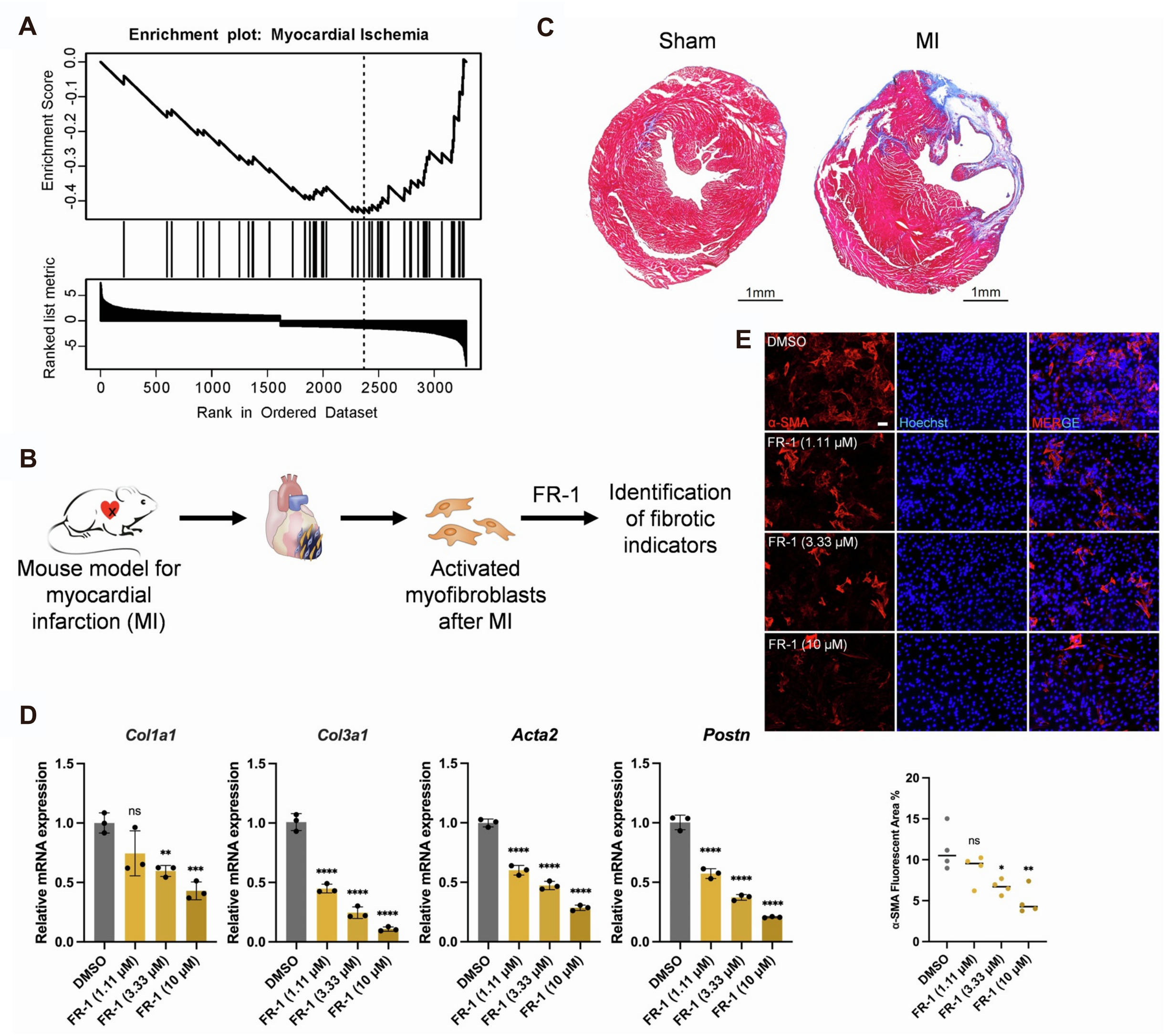

Figure S11. FR-1 attenuates myocardial fibrosis following myocardial infarction (MI), related to Figure 7. (A) GSEA plot depicting downregulation of the DisGeNET "Myocardial Ischemia" gene set in FR-1-treated samples compared to Rottlerin. (B) Schematic illustration of the experimental design. (C) Representative Masson's trichrome staining of cardiac tissue sections from Sham and MI groups, confirming model establishment. Scale bar: 1 mm. (D) RT-qPCR quantification of fibrosis-associated markers in cardiac myofibroblasts treated with FR-1 (n=3). (E) Representative immunofluorescence images (scale bar, 100 μm) and quantification of α-SMA positive area in FR-1-treated myofibroblasts (n=4). Data are mean ± SD, n represents the number of independent biological replicates. $*P < 0.05$; $**P < 0.01$; $***P < 0.001$; $****P < 0.0001$ vs. DMSO by one-way ANOVA.

Table S1. Small molecule list used in chemical screening, related to Figure 1.

Number	Name	Function	Reference
1	Sorafenib	Anti-fibrosis	Hong F et al., 2013[1]
2	Vitamin A	Anti-fibrosis	Murakami K et al., 2011[2]
3	Protocatechuic acid	Anti-fibrosis	Cui B et al., 2021[3]
4	SB431542	Anti-fibrosis	Koh RY et al., 2015[4]
5	Rosiglitazone	Anti-fibrosis	Yu W et al., 2017[5]
6	Nintedanib	Anti-fibrosis	Wuyts WA et al., 2025[6]
7	L-ascorbic acid (Vitamin C)	Anti-fibrosis	Rodrigues da Silva M et al., 2018[7]
8	DB1976	Anti-fibrosis	Wohlfahrt T et al., 2019[8]
9	ICG-001	Anti-fibrosis	Hirakawa T et al., 2019[9]
10	Bosentan	Anti-fibrosis	Clozel M et al., 2005[10]
11	GW788388	Anti-fibrosis	Gellibert F et al., 2006[11]
12	JQ1	Anti-fibrosis	Ijaz et al., 2017[12]
13	Repsox	Anti-fibrosis	Ide M et al., 2017[13]
14	Rottlerin	Anti-fibrosis	Jimenez SA et al., 2001[14]
15	Triamcinolone	Anti-fibrosis	Carroll LA et al., 2002[15]
16	bFGF	Anti-fibrosis	Eto et al., 2012[16]
17	Imatinib	Anti-fibrosis	Distler JH et al., 2007[17]
18	CAY10585	Anti-fibrosis	Kang Y et al., 2020[18]
19	5-FU	Anti-fibrosis	Wendling J et al., 2003[19]
20	SAR-100842	Anti-fibrosis	Ledein L et al., 2020[20]
21	AD*	Adipogenesis	Plikus MV et al., 2017[21]
22	AD*+MSC2530818	Adipogenesis	Li J et al., 2023[22]
23	AD*+Repsox	Adipogenesis	Li J et al., 2023[22]

AD*: Adipocyte Differentiation (AD) medium (DMEM/high glucose, 1% ITS, 0.5mM isobutylmethylxanthine, 0.1 μM cortisol, 1 μM dexamethasone, 0.2 nM triiodothyronine, and 1 μM rosiglitazone)

Table S2. Patient baseline characteristics, related to Figure 1 and 2.

Patient ID	Gender	Age/year	Scar location	Pathological properties
1	male	70	Shoulder	Keloid
2	male	43	Thorax	Keloid
3	female	25	Dorsum	Keloid
4	male	30	Nucha	Hypertrophic scar
5	male	8	Shoulder	Hypertrophic scar
6	female	42	Axilla	Hypertrophic scar
7	male	24	Dorsum	Keloid
8	male	44	Dorsum	Keloid
9	male	47	Dorsum	Keloid
10	male	23	Thorax	Keloid

Table S3. Physicochemical properties and *in vitro* ADME profiles of Rottlerin and FR-1, related to Figure 2.

Pharmaceutical properties		Rottlerin	FR-1
Log*P*	(pH=7.4)	2.11	2.24
Solubility	PBS (pH 7.4, μM)	0.006	1.79
Solubility	Thermodynamic (EtOH, μM)		1973
Caco-2	AB; BA (*1E-6 cm/s); ER	<0.002; 0.03; >20.21	<0.001; 0.02; >24.97
PPB	(m/r/d/c/h bound%)	NA	NA
CYP450 inhibition	3A4–M; 3A4–T; 2C9; 1A2; 2C19; 2D6 (μM)	13.56; 11.06; 2.18; >30; 11.39; >30	6.76; 4.56; 4.22; >30; 9.82; >30
Hepatocyte Cl_{int}	(m/r/d/c/h, μL/min/10e6 cell)	-/-/-/-/101	-/-/-/137/94
Liver microsome stability	(m/r/d/c/h, μL/min/mg protein)	12/<10/<10/<10/<10	66/14/<10/<10/<10

Table S4. List of genes in the IL-17 and TNF signaling pathways significantly downregulated by FR-1 compared to Rottlerin, related to Figure 2.

	gene	baseMean	log2FoldChange	lfcSE	stat	pvalue	padj
IL-17 signaling pathway	S100A7	44.5087239	-7.5862814	1.5548636	-4.8790655	1.07E-06	6.61E-06
	CXCL10	20.9571212	-4.8822574	1.17256935	-4.163726	3.13E-05	0.00014341
	FOSB	633.677965	-4.7532318	0.27664956	-17.181418	3.66E-66	2.41E-63
	CCL20	22.2659731	-4.5358265	1.07069883	-4.2363234	2.27E-05	0.00010718
	FOS	253.288203	-3.5330001	0.3334544	-10.595152	3.14E-26	2.04E-24
	NFKBIA	6072.94225	-3.4625823	0.18491155	-18.725614	3.06E-78	4.03E-75
	IL17RE	61.1220693	-2.4614057	0.56488108	-4.3573875	1.32E-05	6.57E-05
	CXCL3	1377.06352	-1.9858448	0.19380948	-10.246376	1.23E-24	6.85E-23
	CEBPB	12364.5634	-1.718274	0.18412157	-9.3322799	1.04E-20	3.92E-19
	TNFAIP3	2443.40998	-1.7142342	0.18516531	-9.2578581	2.09E-20	7.60E-19
	PTGS2	2852.24613	-1.6127782	0.17094951	-9.4342371	3.94E-21	1.57E-19
	IL1B	107.609993	-1.5725625	0.43873138	-3.5843401	0.00033793	0.00123919
	TRAF4	1048.84018	-1.4927844	0.19901842	-7.5007347	6.35E-14	1.15E-12
	CASP3	3512.12866	-1.3924707	0.16820997	-8.27817	1.25E-16	3.06E-15
	CXCL1	703.631099	-1.2347801	0.23185365	-5.3256877	1.01E-07	7.54E-07
	CXCL5	232.946341	-1.1526656	0.29354807	-3.9266671	8.61E-05	0.00036145
	JUND	4037.33619	-1.1490991	0.20747818	-5.5384092	3.05E-08	2.49E-07
	CXCL2	1711	-1.1199794	0.21335134	-5.24946	1.53E-07	1.11E-06
	NFKB1	2179.80823	-1.1041012	0.18603403	-5.9349419	2.94E-09	2.80E-08
	MMP3	1698.76776	-1.094812	0.23048137	-4.7501106	2.03E-06	1.19E-05
TNF signaling pathway	CXCL10	20.9571212	-4.8822574	1.17256935	-4.163726	3.13E-05	0.00014341
	CCL20	22.2659731	-4.5358265	1.07069883	-4.2363234	2.27E-05	0.00010718
	EDN1	128.624042	-4.194698	0.44802208	-9.3627038	7.77E-21	2.97E-19
	FOS	253.288203	-3.5330001	0.3334544	-10.595152	3.14E-26	2.04E-24
	JUNB	3432.33032	-3.4635734	0.19390125	-17.862564	2.31E-71	1.82E-68
	NFKBIA	6072.94225	-3.4625823	0.18491155	-18.725614	3.06E-78	4.03E-75
	MAP3K8	469.506697	-3.3913276	0.26126905	-12.980212	1.58E-38	2.58E-36
	JAG1	1164.50326	-2.6995474	0.21663598	-12.461214	1.22E-35	1.65E-33
	LIF	621.524807	-2.5766297	0.24118745	-10.6831	1.22E-26	8.38E-25
	NOD2	69.4776428	-2.3287552	0.51244122	-4.5444339	5.51E-06	2.97E-05
	SOCS3	892.957563	-2.231285	0.21093914	-10.577861	3.77E-26	2.44E-24
	ICAM1	1846.37085	-2.2040752	0.1898566	-11.609157	3.70E-31	3.52E-29
	MAP2K3	4384.72628	-2.0354893	0.16926399	-12.025531	2.61E-33	3.05E-31
	CXCL3	1377.06352	-1.9858448	0.19380948	-10.246376	1.23E-24	6.85E-23
	CEBPB	12364.5634	-1.718274	0.18412157	-9.3322799	1.04E-20	3.92E-19
	TNFAIP3	2443.40998	-1.7142342	0.18516531	-9.2578581	2.09E-20	7.60E-19
	BCL3	611.346371	-1.7034416	0.22676915	-7.511787	5.83E-14	1.07E-12
	DAB2IP	1613.26437	-1.6270082	0.2220823	-7.3261498	2.37E-13	3.98E-12
	PTGS2	2852.24613	-1.6127782	0.17094951	-9.4342371	3.94E-21	1.57E-19
	BIRC3	1516.32705	-1.579756	0.20057545	-7.8761183	3.38E-15	7.23E-14
	IL1B	107.609993	-1.5725625	0.43873138	-3.5843401	0.00033793	0.00123919
	PIK3R3	350.413681	-1.567037	0.2987538	-5.2452453	1.56E-07	1.13E-06

IRF1	3903.84613	-1.4963101	0.17043036	-8.7795983	1.64E-18	4.95E-17
RHBDF2	352.498134	-1.4194575	0.27703578	-5.1237335	3.00E-07	2.06E-06
CASP3	3512.12866	-1.3924707	0.16820997	-8.27817	1.25E-16	3.06E-15
CXCL1	703.631099	-1.2347801	0.23185365	-5.3256877	1.01E-07	7.54E-07
MAP2K1	3713.39438	-1.2217254	0.1690846	-7.2255278	4.99E-13	8.15E-12
CXCL5	232.946341	-1.1526656	0.29354807	-3.9266671	8.61E-05	0.00036145
CREB3L1	4410.53481	-1.1458371	0.1715795	-6.6781702	2.42E-11	3.13E-10
CXCL2	1711	-1.1199794	0.21335134	-5.24946	1.53E-07	1.11E-06
NFKB1	2179.80823	-1.1041012	0.18603403	-5.9349419	2.94E-09	2.80E-08
MMP3	1698.76776	-1.094812	0.23048137	-4.7501106	2.03E-06	1.19E-05
MLKL	196.794846	-1.0930032	0.31203793	-3.5027896	0.00046041	0.00163512
MMP14	94307.5748	-1.0929128	0.17930697	-6.0952055	1.09E-09	1.10E-08

Table S5. Mass balance and cutaneous biodistribution of FR-1, related to Figure 3.

Compartment	Drug Content (μg) [Mean ± SD]	Percentage of Dose (%)
Surface Residue	3126.5 ± 142.3	98.4 ± 0.5
Stratum Corneum	1.02 ± 0.33	0.03 ± 0.01
Epidermis	1.30 ± 0.49	0.04 ± 0.01
Dermis	1.81 ± 0.13*	0.06 ± 0.01
Receptor Fluid	N.D.	0
Total Recovery	3130.6 ± 145.2	101.4 ± 2.1

Notes:
Data are presented as mean ± SD (n=3), n represents the number of independent skin donors (biological replicates).
N.D.: Not Detected.
*The tissue concentration in the dermis was calculated to be 2.88 ± 0.32 μg/g.

Table S6. Inhibitory activities of PKC inhibitors, related to Figure 6.

Small Molecules	Protein Kinase C Family	IC50	Reference
Rottlerin	PKCα	30-42 μM	Gschwendt M et al., 1994[23]. María Rosa López-Huertas et al., 2011[24].
	PKCβ	30-42 μM	
	PKCγ	30-42 μM	
	PKCδ	3-6 μM	
	PKCη	80-100 μM	
	PKCε	80-100 μM	
	PKCζ	80-100 μM	
GO6983	PKCα	7 nM	Gschwendt M et al., 1996[25].
	PKCβ	7 nM	
	PKCγ	6 nM	
	PKCδ	10 nM	
	PKCη	/	
	PKCε	/	
	PKCζ	60 nM	
Sotrastaurin	PKCα	0.95 nM	Evenou JP et al., 2009[26].
	PKCβ	0.64 nM	
	PKCγ	/	
	PKCδ	2.1 nM	
	PKCη	1.8 nM	
	PKCε	3.2 nM	
	PKCζ	/	

Table S7. Specific arrangement of small molecules and corresponding barcodes, related to METHOD DETAILS.

Plate	1	2	3	4	5	6	7	8	9	10	11	12
A	DMSO	DB1976	Imatinib	Nintedanib	DMSO	DB1976	Imatinib	Nintedanib	DMSO	DB1976	Imatinib	Nintedanib
B	DMSO	JQ1	Rosiglitazone	TGFβ1	DMSO	JQ1	Rosiglitazone	TGFβ1	DMSO	JQ1	Rosiglitazone	TGFβ1
C	DMSO	Repsox	CAY10585	AD	DMSO	Repsox	CAY10585	AD	DMSO	Repsox	CAY10585	AD
D	DMSO	SB431542	ICG-001	AD+MSC2530818	DMSO	SB431542	ICG-001	AD+MSC2530818	DMSO	SB431542	ICG-001	AD+MSC2530818
E	DMSO	Rottlerin	5-FU	AD+Repsox	DMSO	Rottlerin	5-FU	AD+Repsox	DMSO	Rottlerin	5-FU	AD+Repsox
F	DMSO	Sorafenib	SAR-100842	L-ascorbic acid	DMSO	Sorafenib	SAR-100842	L-ascorbic acid	DMSO	Sorafenib	SAR-100842	L-ascorbic acid
G	DMSO	Triamcinolone	Bosentan	Vitamin A (retinol)	DMSO	Triamcinolone	Bosentan	Vitamin A (retinol)	DMSO	Triamcinolone	Bosentan	Vitamin A (retinol)
H	DMSO	bFGF	GW788388	Protocatechuic acid	DMSO	bFGF	GW788388	Protocatechuic acid	DMSO	bFGF	GW788388	Protocatechuic acid

well_position	Barcode	well_position	Barcode	well_position	Barcode
A1	AACAAGGTAC	C9	AATCCTACCA	F5	ACGCCTTCGT
A2	AACAATCAGG	C10	AATCGTCCGC	F6	ACGCTGGATA
A3	AACATGGAGA	C11	AATGAGAGCA	F7	ACGTGCTGAT
A4	AACATTACCG	C12	AATGTCAGTG	F8	ACTCCAAGCC
A5	AACCGCGACT	D1	ACAACAGTCG	F9	ACTTAACTGC
A6	AACCGGAAGG	D2	ACAACCATAC	F10	ACTTCATCAC
A7	AACCTCATAG	D3	ACACAATCTC	F11	ACTTGAGGAA
A8	AACGTAAGCT	D4	ACACAGTGAA	F12	ACTTGTAAGG
A9	AAGACGGATT	D5	ACACGGTCCT	G1	AGACCGTTAT
A10	AAGATCGGCG	D6	ACACTTGCTG	G2	AGACTAGCAT
A11	AAGCGATGTT	D7	ACCAGGACCA	G3	AGAGTGTAAC
A12	AAGCGTTCAG	D8	ACCATAACAC	G4	AGAGTTCTGC
B1	AAGGTCTGGA	D9	ACCGGTACAG	G5	AGCATGTCAT
B2	AAGTTAGCGC	D10	ACCGTACTTC	G6	AGCCACTAGC
B3	AATAGCCACA	D11	ACCTGTCCGA	G7	AGCGATAACG

B4	AATCACGCGA	D12	ACCTTATGTG	G8	AGCGTACAAT
B5	AACACCTAGT	E1	AATGAACACG	G9	ACGGTCCGTT
B6	AACAGGCAAT	E2	AATGACCTTC	G10	ACGTAGGCAC
B7	AACCAGCCAG	E3	AATTAGGCCG	G11	ACTGGCGCAT
B8	AACCAGTTGA	E4	AATTGCGATG	G12	ACTGGCTTCC
B9	AACCGGCGTA	E5	ACAACGGAGC	H1	ACTTCGTTGA
B10	AACCTAGTCC	E6	ACAAGCGCGA	H2	ACTTCTCCTG
B11	AACTCTACAC	E7	ACACCGAATT	H3	AGAACCACGG
B12	AACTGTGTCA	E8	ACACGCAGTA	H4	AGAAGCAATC
C1	AAGATGTCCA	E9	ACAGTGCCAA	H5	AGAGATGCAG
C2	AAGCATATGG	E10	ACATGTGTGC	H6	AGAGCTTACA
C3	AAGCTCACCT	E11	ACCGAACCGT	H7	AGATAGTGCT
C4	AAGGCATGCG	E12	ACCGAGAGTC	H8	AGCAATGCGC
C5	AAGTTCCTTG	F1	ACCTCCGACA	H9	AGCCAGAATA
C6	AATACCGGTA	F2	ACCTCTCTCC	H10	AGCCAGCTCT
C7	AATCCATCTG	F3	ACGAATGACA	H11	AGCTATTCCA
C8	AATCCGCTCC	F4	ACGCCTCAAC	H12	AGCTCCTCAG

SUPPLEMENTAL REFERENCES

1. Hong, F., Chou, H., Fiel, M.I., and Friedman, S.L. (2013). Antifibrotic activity of sorafenib in experimental hepatic fibrosis: refinement of inhibitory targets, dosing, and window of efficacy in vivo. Dig Dis Sci *58*, 257-264. 10.1007/s10620-012-2325-y.
2. Murakami, K., Kaji, T., Shimono, R., Hayashida, Y., Matsufuji, H., Tsuyama, S., Maezono, R., Kosai, K., and Takamatsu, H. (2011). Therapeutic effects of vitamin A on experimental cholestatic rats with hepatic fibrosis. Pediatr Surg Int *27*, 863-870. 10.1007/s00383-011-2853-0.
3. Cui, B., Yang, Z., Wang, S., Guo, M., Li, Q., Zhang, Q., and Bi, X. (2021). The protective role of protocatechuic acid against chemically induced liver fibrosis in vitro and in vivo. Pharmazie *76*, 232-238. 10.1691/ph.2021.0909.
4. Koh, R.Y., Lim, C.L., Uhal, B.D., Abdullah, M., Vidyadaran, S., Ho, C.C., and Seow, H.F. (2015). Inhibition of transforming growth factor-β via the activin receptor-like kinase-5 inhibitor attenuates pulmonary fibrosis. Mol Med Rep *11*, 3808-3813. 10.3892/mmr.2015.3193.
5. Yu, W., Mi, L., and Long, T. (2017). Efficacies of rosiglitazone and retinoin on bleomycin-induced pulmonary fibrosis in rats. Exp Ther Med *14*, 609-615. 10.3892/etm.2017.4555.
6. Wuyts, W.A., Bonella, F., Chaudhuri, N., Varone, F., Antin-Ozerkis, D., Song, J.W., Miede, C., Dumistracel, M., Coeck, C., and Cottin, V. (2025). Continued Treatment with Nintedanib in Patients with Progressive Pulmonary Fibrosis: Data from INBUILD-ON. Lung *203*, 25. 10.1007/s00408-024-00778-z.
7. Rodrigues da Silva, M., Schapochnik, A., Peres Leal, M., Esteves, J., Bichels Hebeda, C., Sandri, S., Pavani, C., Ratto Tempestini Horliana, A.C., Farsky, S.H.P., and Lino-Dos-Santos-Franco, A. (2018). Beneficial effects of ascorbic acid to treat lung fibrosis induced by paraquat. PLoS One *13*, e0205535. 10.1371/journal.pone.0205535.
8. Wohlfahrt, T., Rauber, S., Uebe, S., Luber, M., Soare, A., Ekici, A., Weber, S., Matei, A.E., Chen, C.W., Maier, C., et al. (2019). PU.1 controls fibroblast polarization and tissue fibrosis. Nature *566*, 344-349. 10.1038/s41586-019-0896-x.
9. Hirakawa, T., Nasu, K., Miyabe, S., Kouji, H., Katoh, A., Uemura, N., and Narahara, H. (2019). β-catenin signaling inhibitors ICG-001 and C-82 improve fibrosis in preclinical models of endometriosis. Sci Rep *9*, 20056. 10.1038/s41598-019-56302-4.
10. Clozel, M., and Salloukh, H. (2005). Role of endothelin in fibrosis and anti-fibrotic potential of bosentan. Ann Med *37*, 2-12. 10.1080/07853890410018925.
11. Gellibert, F., de Gouville, A.C., Woolven, J., Mathews, N., Nguyen, V.L., Bertho-Ruault, C., Patikis, A., Grygielko, E.T., Laping, N.J., and Huet, S. (2006). Discovery of 4-{4-[3-(pyridin-2-yl)-1H-pyrazol-4-yl]pyridin-2-yl}-N-(tetrahydro-2H- pyran-4-yl)benzamide (GW788388): a potent, selective, and orally active transforming growth factor-beta type I receptor inhibitor. J Med Chem *49*, 2210-2221. 10.1021/jm0509905.
12. Ijaz, T., Jamaluddin, M., Zhao, Y., Zhang, Y., Jay, J., Finnerty, C.C., Herndon, D.N., Tilton, R.G., and Brasier, A.R. (2017). Coordinate activities of BRD4 and CDK9 in the transcriptional elongation complex are required for TGFbeta-induced Nox4 expression and myofibroblast transdifferentiation. Cell Death Dis *8*, e2606. 10.1038/cddis.2016.434.
13. Ide, M., Jinnin, M., Tomizawa, Y., Wang, Z., Kajihara, I., Fukushima, S., Hashizume, Y., Asano, Y., and Ihn, H. (2017). Transforming growth factor β-inhibitor Repsox downregulates collagen expression of scleroderma dermal fibroblasts and prevents bleomycin-induced mice skin fibrosis. Exp Dermatol *26*, 1139-1143. 10.1111/exd.13366.
14. Jimenez, S.A., Gaidarova, S., Saitta, B., Sandorfi, N., Herrich, D.J., Rosenbloom, J.C., Kucich, U., Abrams, W.R., and Rosenbloom, J. (2001). Role of protein kinase C-delta in the regulation of collagen gene expression in scleroderma fibroblasts. J Clin Invest *108*, 1395-1403. 10.1172/jci12347.
15. Carroll, L.A., Hanasono, M.M., Mikulec, A.A., Kita, M., and Koch, R.J. (2002). Triamcinolone stimulates bFGF production and inhibits TGF-beta1 production by human dermal fibroblasts. Dermatol Surg *28*, 704-709. 10.1046/j.1524-4725.2002.02012.x.
16. Eto, H., Suga, H., Aoi, N., Kato, H., Doi, K., Kuno, S., Tabata, Y., and Yoshimura, K. (2012). Therapeutic potential of fibroblast growth factor-2 for hypertrophic scars: upregulation of MMP-1 and HGF expression. Lab Invest *92*, 214-223. 10.1038/labinvest.2011.127.
17. Distler, J.H., Jüngel, A., Huber, L.C., Schulze-Horsel, U., Zwerina, J., Gay, R.E., Michel, B.A., Hauser, T., Schett, G., Gay, S., and Distler, O. (2007). Imatinib mesylate reduces production of extracellular matrix and prevents development of experimental dermal fibrosis. Arthritis Rheum *56*, 311-322. 10.1002/art.22314.

18. Kang, Y., Roh, M.R., Rajadurai, S., Rajadurai, A., Kumar, R., Njauw, C.N., Zheng, Z., and Tsao, H. (2020). Hypoxia and HIF-1α Regulate Collagen Production in Keloids. J Invest Dermatol *140*, 2157-2165. 10.1016/j.jid.2020.01.036.
19. Wendling, J., Marchand, A., Mauviel, A., and Verrecchia, F. (2003). 5-fluorouracil blocks transforming growth factor-beta-induced alpha 2 type I collagen gene (COL1A2) expression in human fibroblasts via c-Jun NH2-terminal kinase/activator protein-1 activation. Mol Pharmacol *64*, 707-713. 10.1124/mol.64.3.707.
20. Ledein, L., Léger, B., Dees, C., Beyer, C., Distler, A., Vettori, S., Boukaiba, R., Bidouard, J.P., Schaefer, M., Pernerstorfer, J., et al. (2020). Translational engagement of lysophosphatidic acid receptor 1 in skin fibrosis: from dermal fibroblasts of patients with scleroderma to tight skin 1 mouse. Br J Pharmacol *177*, 4296-4309. 10.1111/bph.15190.
21. Plikus, M.V., Guerrero-Juarez, C.F., Ito, M., Li, Y.R., Dedhia, P.H., Zheng, Y., Shao, M., Gay, D.L., Ramos, R., Hsi, T.C., et al. (2017). Regeneration of fat cells from myofibroblasts during wound healing. Science *355*, 748-752. 10.1126/science.aai8792.
22. Li, J., Bai, Y., Liu, Y., Song, Z., Yang, Y., and Zhao, Y. (2023). Transcriptome-based chemical screens identify CDK8 as a common barrier in multiple cell reprogramming systems. Cell Rep *42*, 112566. 10.1016/j.celrep.2023.112566.
23. Gschwendt, M., Müller, H.J., Kielbassa, K., Zang, R., Kittstein, W., Rincke, G., and Marks, F. (1994). Rottlerin, a novel protein kinase inhibitor. Biochem Biophys Res Commun *199*, 93-98. 10.1006/bbrc.1994.1199.
24. López-Huertas, M.R., Mateos, E., Díaz-Gil, G., Gómez-Esquer, F., Sánchez del Cojo, M., Alcamí, J., and Coiras, M. (2011). Protein kinase Ctheta is a specific target for inhibition of the HIV type 1 replication in CD4+ T lymphocytes. J Biol Chem *286*, 27363-27377. 10.1074/jbc.M110.210443.
25. Gschwendt, M., Dieterich, S., Rennecke, J., Kittstein, W., Mueller, H.J., and Johannes, F.J. (1996). Inhibition of protein kinase C mu by various inhibitors. Differentiation from protein kinase c isoenzymes. FEBS Lett *392*, 77-80. 10.1016/0014-5793(96)00785-5.
26. Evenou, J.P., Wagner, J., Zenke, G., Brinkmann, V., Wagner, K., Kovarik, J., Welzenbach, K.A., Weitz-Schmidt, G., Guntermann, C., Towbin, H., et al. (2009). The potent protein kinase C-selective inhibitor AEB071 (sotrastaurin) represents a new class of immunosuppressive agents affecting early T-cell activation. J Pharmacol Exp Ther *330*, 792-801. 10.1124/jpet.109.153205.
